# Red cabbage anthocyanin-rich natural colorant: Optimization of ultrasound-assisted extraction, microencapsulation, bioaccessibility, and functional yogurt development

**DOI:** 10.1016/j.ultsonch.2025.107702

**Published:** 2025-12-04

**Authors:** Sumandeep Kaur, Amisha Rani, Reshma Thakur, Abhilasha Sharma, Kritika Kuksal, Aman Sharma, Arti Shivraj Nile, Shivraj Hariram Nile

**Affiliations:** a Division of Food and Nutritional Biotechnology, BRIC-National Agri-Food and Biomanufacturing Institute (Formerly National Agri-Food Biotechnology Institute), Sector-81, Mohali 140306 Punjab, India; b Department of Food Science and Technology, School of Health Sciences, Amity University, Sector-82A, Sahibzada Ajit Singh Nagar, 140306 Punjab, India

**Keywords:** Anthocyanins, Microencapsulation, Ultrasound, Stability, Bioaccessibility, Red cabbage

## Abstract

Anthocyanins from red cabbage (*Brassica oleracea* var. *capitata* f. *rubra*) are promising natural pigments with notable health benefits, but their thermal and gastrointestinal instability limits functional food applications. This study employed ultrasound-assisted extraction (UAE) and spray-drying microencapsulation to enhance their stability, bioaccessibility, and functionality. Optimization using a Box–Behnken design (pH 3.0–5.0, 30–60 °C, 20–90  min, 0.025–0.05  g/mL solid–liquid ratio) identified optimal UAE conditions (pH 3.5, 45 °C, 55  min, 0.0375  g/mL), yielding 125.72 ± 0.83  mg/L anthocyanins. Freeze drying retained the highest anthocyanin content (129.02 ± 3.36  mg/L), while spray-drying with 10 % maltodextrin achieved excellent encapsulation efficiency (96.36 ± 0.80 %) and yield (99.40 %). FTIR confirmed the presence of phenolic groups, and SEM revealed spherical microcapsules (∼10.27  µm) with smooth surfaces. Encapsulation significantly improved thermal stability at 70 °C, reducing the degradation rate constant (0.00043  min^−1^) and increasing half-life (1919.02  min), compared to non-encapsulated samples. During simulated digestion, bioaccessibility of encapsulated anthocyanins was lower in the oral (53.28 %) and intestinal (25.37 %) phases than free forms, but stability was enhanced. Antioxidant activity (DPPH: 66.90 %, ABTS: 71.76 %) and enzyme inhibition (α-amylase IC_50_: 46.00  µg/mL, α-glucosidase IC_50_: 51.51  µg/mL) indicated potent bioactivity. Cytotoxicity testing on HEK-293 cells showed > 90 % viability, confirming safety. Yogurt fortified with 3 % encapsulated powder received high sensory scores (8.30 ± 0.86), especially for color and taste. These findings validate the integrated UAE and encapsulation approach as a scalable method to stabilize red cabbage anthocyanins for functional food applications.

## Graphical abstract

**Figure:**
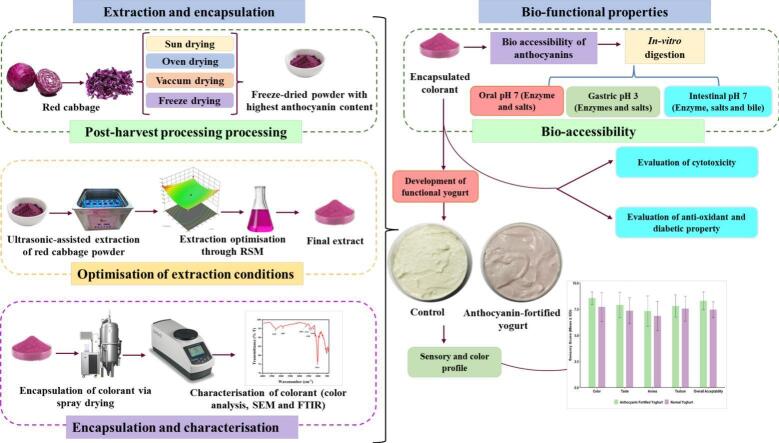

## 1 Introduction

The increasing global demand for safer, health-promoting, and sustainably produced food ingredients has intensified an unprecedented interest in bioactive phytochemicals, with particular emphasis on plant-derived pigments. Among plant-derived pigments, anthocyanins have emerged as multifunctional compounds of considerable industrial and scientific significance [1]. These water-soluble flavonoid pigments not only provide vibrant red, blue, and purple hues to plant tissues but also exhibit a range of physiological functions, including free radical scavenging, anti-inflammatory effects, and chemopreventive actions against chronic diseases such as cardiovascular disorders, obesity, and cancer [2], [3], [4]. The shift toward natural pigments is primarily driven by increasing awareness of the potential health hazards linked to synthetic colorants [5]. Artificial dyes, valued for their vibrant colors and stability in food and beverages, raise safety concerns like allergenicity, genotoxicity, and potential carcinogenicity, alongside environmental issues during production and degradation. Anthocyanins, natural plant-based colorants, offer health benefits and vivid hues but face challenges due to their instability against temperature, pH, oxygen, light, and enzymes, limiting their use in functional foods [6]. Red cabbage (*Brassica oleracea* var. capitata f. rubra) is a promising anthocyanin source, rich in acylated cyanidin derivatives that resist degradation due to steric protection from hydroxycinnamic acids [7]. However, their extraction and application are hindered by degradation during processing. Traditional solvent extraction methods are inefficient, time-consuming, and degrade thermolabile anthocyanins while co-extracting impurities [8]. Ultrasound-assisted extraction (UAE) offers a sustainable alternative, using cavitation to disrupt plant cell walls, enhance anthocyanin release, and minimize thermal degradation. UAE’s compatibility with green solvents and lower temperatures preserves anthocyanin bioactivity, making it a promising method for efficient, eco-friendly extraction [9].

Optimizing UAE requires precise calibration of factors such as solvent composition, solid–liquid ratio, sonication time, amplitude, temperature, and pH. Response surface methodology (RSM), using designs like central composite or Box–Behnken, enables systematic evaluation of interactions and predictive optimization of extraction yield [10], [11]. Efficient extraction alone cannot ensure anthocyanin stability, as these compounds are highly susceptible to degradation during processing and storage. Encapsulation offers protection by isolating anthocyanins from environmental stress, enhancing stability, dispersibility, and controlled release. Among available methods, spray drying is the most economical and widely used, converting liquid extracts into stable powders through rapid drying under controlled conditions [12], [13]. Spray-drying efficiency largely depends on the choice of encapsulating agent. Maltodextrin, a partially hydrolyzed starch, is widely preferred for its low viscosity at high solids, excellent film-forming ability, high solubility, and regulatory acceptance in food applications [13], [14].

Beyond improving storage stability, encapsulation aids in controlling anthocyanin release and transformation in the gastrointestinal tract. Although rich in antioxidant potential, anthocyanins exhibit low bioavailability due to instability at neutral pH, enzymatic degradation, and poor intestinal absorption. Simulated digestion studies confirm their substantial structural breakdown during digestion [15], [16], resulting in lowered bioaccessibility and, ultimately, reduced physiological efficacy [17]. Encapsulation improves anthocyanin stability and bioaccessibility by protecting them from degradation in the upper gastrointestinal tract and enabling controlled intestinal release. In vitro digestion models, such as INFOGEST, simulate oral-to-intestinal conditions to assess stability, structural changes, and bioavailability. Cytotoxicity assays using human cell lines further ensure biosafety of encapsulated formulations. While generally recognized as safe, comprehensive toxicological evaluation remains essential for regulatory approval. Moreover, red cabbage anthocyanins exhibit potent antioxidant activity, effectively scavenging reactive oxygen species and chelating metal ions [18], and modulate enzymatic pathways involved in oxidative stress responses. Additionally, anthocyanins inhibit key carbohydrate-hydrolyzing enzymes, such as α-amylase and α-glucosidase [19], which are critical in regulating postprandial glucose metabolism. By slowing carbohydrate hydrolysis and glucose absorption, anthocyanins may contribute to glycemic control, offering potential benefits for managing or preventing type II diabetes and related metabolic disorders. Despite advancements in anthocyanin extraction, stabilization, and characterization, substantial gaps in translational and mechanistic understanding remain, necessitating standardized processes to ensure batch-to-batch reproducibility, effective integration of encapsulated anthocyanins into complex food matrices with validated sensory and nutritional outcomes, and detailed elucidation of structure–function relationships for specific anthocyanin species and their metabolites. In light of these considerations, the present study integrates ultrasound-assisted extraction, RSM-based optimization, and maltodextrin spray-drying encapsulation to enhance the recovery and stability of red cabbage anthocyanins. Comprehensive analyses of antioxidant potential, enzymatic inhibition, digestion behavior, and cytotoxicity establish their suitability for functional food and nutraceutical applications.

## 2 Materials and methods

### 2.1 Plant material and chemicals

Fresh red cabbage (*Brassica oleracea* L. var. capitata f. rubra) was procured from the fruit and vegetable market in Sector 26, Chandigarh, India. All chemicals used in the study were of analytical and HPLC grade obtained from Himedia Laboratories Pvt. Ltd. (Mumbai, India). These included ethanol (≥99.9 % purity), Folin–Ciocalteu reagent, sodium carbonate (Na_2_CO), potassium chloride (KCl), sodium acetate (CH_3_COONa), aluminum chloride (AlCl_3_), 2,2-diphenyl-1-picrylhydrazyl (DPPH), gallic acid, and quercetin. MTT, potassium chloride, sodium chloride, monopotassium phosphate, PBS, FBS (fetal bovine serum), DPPH (2,2-diphenyl-1-picrylhydrazyl), magnesium chloride hexahydrate, α-amylase, α-glucosidase from Saccharomyces cerevisiae, HPLC grade distilled water, methanol, potassium acetate, aluminium chloride, DMSO, sodium biocarbonate, ABTS (2,2′-azino-bis(3-ethylbenzothiazoline-6-sulfonic acid), Folin–Ciocalteu reagent, maltodextrin, acarbose, citric acid, 4-Nitrophenyl β-D-glucopyranoside (pNPG), salivary amylase from human saliva, pepsin from porcine gastric mucosa, bile from bovine, pancreatin from porcine pancreas, were purchased from Sigma Aldrich (St. Louis, USA). Human embryonic kidney cells (HEK) cell line was obtained from National Centre for Cell Science, Pune, India. DMEM and antibiotic (penistrep) were purchased from Gibco.

### 2.2 Processing of red cabbage

The freshly procured red cabbage was manually trimmed to remove outer leaves, washed under running tap water, and chopped into uniform pieces. The chopped cabbage was subjected to distinct drying treatments to assess the impact on anthocyanin recovery. This comparative evaluation of drying methods was included as a pre-processing optimization step to determine the most suitable technique for preserving anthocyanin stability prior to ultrasound-assisted extraction (UAE). For sun drying, samples were exposed to natural sunlight for 72  h (until constant dry weight achieved). Oven drying was conducted at 50 °C in a hot-air oven for 24  h until constant weight. Vacuum drying was performed in a vacuum dryer at 40 °C under 500  mmHg pressure for 20  h. For freeze drying, samples were pre-frozen at − 80 °C for 12  h and then lyophilized for 36  h using a lyophilizer. All dried samples were ground using a laboratory blender and sieved through a 60-mesh stainless-steel sieve to obtain a homogenous powder. The powdered materials were stored in airtight, opaque polyethylene containers at 4 °C until further analysis.

### 2.3 Estimation of total anthocyanin content

Total anthocyanin content (TAC) in the extracts obtained from differently dried red cabbage powders was quantified using the pH differential method [20], with minor modifications. Each extract was diluted separately with 0.025  M potassium chloride buffer (pH 1.0) and 0.4  M sodium acetate buffer (pH 4.5) in a 1:5 ratio. The solutions were incubated in the dark for 15  min, and absorbance was recorded at 520  nm and 700  nm using a UV–Visible spectrophotometer (Shimadzu UV-1800, Japan). The anthocyanin concentration was calculated using the following formula and expressed as mg cyanidin-3-glucoside equivalents per gram of dry weight (mg C3GE/g d.w.)

(1) $$\mathrm{Totalmonomericanthocyanins}\left(mgCy3G/L\right)\;=\;\frac{A\;x\;MW\;x\;DF\;x\;1000}{\varepsilon \;x\;l}$$

where

A = (A_520_ – A_700_) pH 1.0 − (A_520_ – A_700_) pH 4.5,

MW = 449.2  g/mol (molecular weight of cyanidin-3-glucoside),

DF = dilution factor,

ε = 26,900 L·mol^−1^·cm^−1^ (molar absorptivity), and.

l = path length (1  cm).

Among the drying methods tested, the extract exhibited the highest anthocyanin content and was therefore selected for further optimization of extraction parameters using response surface methodology.

### 2.4 Optimization of ultrasound-assisted extraction parameters

Optimization of anthocyanin extraction from freeze-dried red cabbage was carried out using Response Surface Methodology (RSM) based on a Box–Behnken design (BBD). Four independent variables were selected for the study: extraction pH (X_1_, 3.0–5.0), extraction time (X_2_, 20–90  min), extraction temperature (X_3_, 30–60 °C), and sample-to-solvent ratio (X_4_, 0.025–0.05  g/mL). Each variable was tested at three levels (−1, 0, +1), and a total of 29 randomized experimental runs, including five center points, were generated using Design-Expert software (Version 12, Stat-Ease Inc., USA). The total anthocyanin content served as the response variable to evaluate the efficiency of each parameter combination. Experimental data were fitted to a second-order polynomial equation, and the adequacy of the model was assessed by analysis of variance (ANOVA). Response surface plots were generated to visualize the interactions among variables, and numerical optimization was performed to determine the combination of factors yielding maximum anthocyanin recovery.

### 2.5 Microencapsulation of optimized anthocyanin extract by spray drying

The anthocyanin-rich extract obtained under optimized ultrasound-assisted extraction conditions was subjected to spray-drying microencapsulation using the conditions standardized in our previous study [21]. Maltodextrin (DE 10–12) was used as the encapsulating wall material at a concentration of 10 % (w/v), prepared by dissolving in the anthocyanin extract under constant stirring. The mixture was homogenized at 10,000  rpm for 10  min using a high-speed homogenizer (Ultra-Turrax T25, IKA, Germany) to obtain a uniform feed solution. Spray drying was carried out using a laboratory-scale spray dryer (BUCHI B-290, Switzerland) equipped with a two-fluid nozzle. The feed solution was introduced at a flow rate of 5  mL/min. The inlet and outlet temperatures were maintained at 150 ± 2 °C and 90 ± 2 °C, respectively. Compressed air was supplied at a pressure of 0.5  bar, and the atomization air flow rate was set at 600 L/h. The resulting encapsulated anthocyanin were collected in the cyclone separator, sealed in moisture-proof containers, and stored at 4 °C in the dark until further characterization.

### 2.6 Untargeted LC-MS analysis of colorant

LC–MS analysis was performed to compare the anthocyanin composition of samples extracted with ultrasound (S2) and without ultrasound (S1) to evaluate the cavitation-induced degradation of anthocyanin and polyphenolic monomers. Chromatographic separation was carried out on a Waters ACQUITY UPLC H-Class System equipped with an ACQUITY BEH C18 column (2.1 × 100 mm, 1.7 µm). The mobile phases consisted of Solvent A (0.1 % formic acid in water) and Solvent B (0.1 % formic acid in acetonitrile), using the following gradient program: 0–5 min, 95 % A; 5–30 min, linear shift to 10 % A; 30–35 min, 10 % A; returning to initial conditions at 36 min and equilibrating until 45 min. The flow rate was 0.2 mL/min with a 5 µL injection volume, and the total run time was 45 min.

Mass spectrometric detection was conducted on a Waters SYNAPT XS HDMS system operated in ESI positive mode under the following conditions: capillary voltage 3.22 kV, cone voltage 50 V, source temperature 120 °C, desolvation temperature 550 °C, desolvation gas flow 950 L/h, cone gas flow 50 L/h, and collision energy 4 eV. Data were acquired in MRM mode using MassLynx v4.2 software. Anthocyanins and phenolic derivatives were identified by comparison of precursor and product ion transitions, retention times, and literature-reported fragmentation patterns.

### 2.7 Characterization of colorants

#### 2.7.1 Surface morphology (SEM)

The surface morphology and structural integrity of encapsulated and non-encapsulated red cabbage colorants were analyzed using a field emission scanning electron microscope (FE-SEM, Hitachi SU8010, Krefeld, Germany). Powder samples were mounted on copper stubs using double-sided carbon tape and sputter-coated with a thin layer of gold under vacuum to improve electrical conductivity. Micrographs were captured at various magnifications under an accelerating voltage of 20  kV. The images were evaluated for particle size, shape, surface uniformity, and morphological features.

#### 2.7.2 Fourier transform infrared Spectroscopy (FTIR)

FTIR analysis was conducted to identify the functional groups present in the encapsulated and non-encapsulated anthocyanin-rich colorants and to confirm successful molecular interaction with the wall matrix. Spectra were recorded using an FTIR spectrometer equipped with an attenuated total reflectance (ATR) accessory (PerkinElmer, Waltham, MA, USA). The infrared spectra were acquired over a wavelength range of 4000–600  cm^−1^ at a resolution of 4  cm^−1^, with 32 scans averaged per sample at room temperature.

#### 2.7.3 Colorimetric analysis

Colorimetric properties of encapsulated and non-encapsulated red cabbage extracts were determined using a spectrocolorimeter (ColorFlex EZ, HunterLab, Virginia, USA) based on the CIE L*a*b* color space. Triplicate readings were obtained for each sample. L* indicates lightness (0 = black, 100 = white), a* denotes the red-green axis (+a* = red), and b* represents the yellow-blue axis (+b* = yellow). Additionally, chroma (C* = √(a*^2^ + b*^2^)) and hue angle (h° = arctangent (b*/a*)) were calculated to further characterize color saturation and tone.

### 2.8 Thermal stability analysis

Thermal stability of the encapsulated and non-encapsulated anthocyanin-based colorants was evaluated by subjecting the samples to controlled heating at three different temperatures: 70 °C, 90 °C, and 120 °C. Aliquots of each sample were placed in sealed glass vials and heated in a thermostatic water bath (Julabo SW22, Germany) for two-time intervals: 30 and 60 min. Immediately after thermal treatment, the samples were cooled in an ice bath. The residual anthocyanin content after thermal exposure was quantified using the pH differential method described in Section 2.4. Thermal degradation kinetics were modeled assuming first-order reaction behavior. The degradation rate constant (k) was calculated using the following equation:

(2) $$ln\left(\frac{C_{t}}{C_{\text{0}}}\right)=\;-k.t$$

Where C_0_ represents the initial concentration of anthocyanins and Ct represents the concentration at time t, k is the degradation rate constant, t is the thermal exposure duration (30 or 60 min). The thermal half-life ($t_{1/2})$ was determined using:

(3) $$t_{1/2}=\frac{\ln(2)}{k}$$

All experiments were conducted in triplicate, and anthocyanin concentrations were used to determine the extent of degradation as a function of temperature and time.

### 2.9 Determination of antioxidant activity

The antioxidant potential of CPP extracts was evaluated using DPPH, ABTS, and FRAP assays [22]. In the DPPH assay, 0.3 mL of extract was mixed with 3 mL of 0.1 mM DPPH solution, incubated at 25 °C for 30 min in the dark, and absorbance measured at 517 nm. The ABTS assay involved generating ABTS by reacting 7.4 mM ABTS with 2.6 mM potassium persulfate, diluting to an absorbance of 0.700 ± 0.02 at 734 nm, and mixing 0.2 mL of extract with 0.8 mL of ABTS solution; absorbance was recorded after 6 min at 734 nm. The FRAP assay measured reducing power by reacting 10 μL of extract with 300 μL of FRAP reagent, incubating at 37 °C for 15 min, and measuring absorbance at 593 nm. Results from all assays were expressed as percentage inhibition or micromoles of Fe^2+^ equivalents per mL of extract.

### 2.10 Colorant application and functional evaluation

#### 2.10.1 Cytotoxicity assessment by MTT assay

The cytocompatibility of digested anthocyanin-based colorants (encapsulated and non-encapsulated) was evaluated using the MTT assay on human embryonic kidney (HEK-293) cells. Cells were cultured in Dulbecco’s Modified Eagle Medium (DMEM) supplemented with 10 % fetal bovine serum (FBS) and 1 % penicillin–streptomycin, and maintained at 37 °C in a humidified incubator containing 5 % CO_2_. HEK-293 cells were seeded into 96-well plates at a density of 5 × 104 cells/mL and incubated overnight to allow adhesion. The cells were then treated with varying concentrations of digested extract samples for 48  h. Following incubation, 10  µL of MTT solution (5  mg/mL) was added to each well and incubated for an additional 4  h. Formazan crystals formed were dissolved in 100  µL of DMSO, and absorbance was measured at 570  nm using a microplate reader (SpectraMax M5e, Molecular Devices, USA). Cell viability was expressed as a percentage relative to untreated controls.

#### 2.10.2 In-vitro antidiabetic activity

The antidiabetic potential of the anthocyanin-based colorants was assessed through in vitro α-amylase and α-glucosidase inhibition assays [23]. For α-amylase activity, 50  µL of extract was incubated with 50  µL of α-amylase enzyme (1 U/mL) in phosphate buffer (pH 6.9) at 37 °C for 10  min, followed by addition of 100  µL soluble starch solution (1 % w/v). After further incubation, the reaction was terminated by adding 100  µL of iodine reagent, and absorbance was recorded at 630  nm. For α-glucosidase inhibition, 50  µL of extract was pre-incubated with 50  µL of α-glucosidase enzyme (1 U/mL) in phosphate buffer (pH 6.8) for 10  min at 37 °C, followed by addition of 100  µL of 1  mM p-nitrophenyl-α-D-glucopyranoside (pNPG). The reaction was stopped after 15  min with 100  µL of 0.1  M Na_2_CO_3_, and absorbance was measured at 405  nm. Acarbose was used as a positive control. Percentage inhibition was calculated using:

(4) $$\alpha -amylase\;inhibition\;\left(\%\right)\;=\;\left(1-\frac{Abs2-Abs1}{Abs4-Abs3}\right)\times 100$$

Absorbance 1 corresponds to the mixture of starch, amylase and sample. Absorbance 2 corresponds to the mixture of starch and sample. Absorbance 3 represents the mixture of amylase and starch, and Absorbance 4 associated with solution containing starch.

(5) $$\alpha -glucosidase\;inhibition\;\left(\%\right)\;=1\;\left[\frac{\mathrm{Blank}-corrected\;absorbance\;of\;test\;well}{\mathrm{Absorbance}\;of\;test\;well\;blank\;-corrected\;absorbance\;of\;negative\;control}\right]\times 100$$

#### 2.10.3 Preparation of anthocyanin-fortified yoghurt and sensory evaluation

To evaluate the practical use of the colorant, anthocyanin-enriched yogurt was prepared and tested for consumer acceptance. Thick, creamy hung curd was made by straining fresh curd through a muslin cloth to remove excess whey. The finished product was divided evenly into two portions. Both were sweetened with 12 % (w/w) xylitol as a sugar substitute. The control sample (Sample A) contained no anthocyanin, while the test sample (Sample B) was fortified with 3 % (w/w) encapsulated anthocyanin powder. The yogurt samples were chilled at 4 °C for 12 h to stabilize their texture and flavor before sensory analysis. Sensory evaluation was performed by a panel of twenty semi-trained individuals (10 males and 10 females, aged 22–29) from the Department of Food and Nutrition Biotechnology familiar with dairy product assessment. Panelists received two training sessions on identifying sensory attributes of anthocyanin-fortified yogurts using a 9-point hedonic scale. Evaluations were carried out in a sensory laboratory maintained at 25 ± 2 °C under white fluorescent lighting. Samples, coded with random three-digit numbers, were served in randomized order, and panelists rinsed their palate with water between evaluations. Panelists rated five sensory attributes, color, taste, aroma, texture, and overall acceptability using a 9-point hedonic scale (1 = dislike extremely; 9 = like extremely). Anthocyanin-fortified and control yogurts were stored at 4 ± 1 °C for 7 days, during which total anthocyanin content (TAC), *color parameters (L, a*, b*)**, and pH were measured at 24-hour intervals to evaluate pigment stability, visual quality, and fermentation behavior under refrigerated conditions.

### 2.11 Statistical analysis

All experiments were conducted in triplicate, and results were expressed as mean ± standard deviation (SD). Data were analyzed using one-way analysis of variance (ANOVA) followed by Tukey’s Honest Significant Difference (HSD) post hoc test to assess significant differences among treatment means at a confidence level of 95 % (p < 0.05). Experimental design modeling and regression analysis for extraction optimization were performed using Design-Expert software (Version 12, Stat-Ease Inc., Minneapolis, USA). Graphical representations and additional statistical computations were carried out using R software (Version 4.4.2) and Microsoft Excel.

## 3 Results and discussion

### 3.1 Effect of drying method on total anthocyanin content

**Fig. 1:**
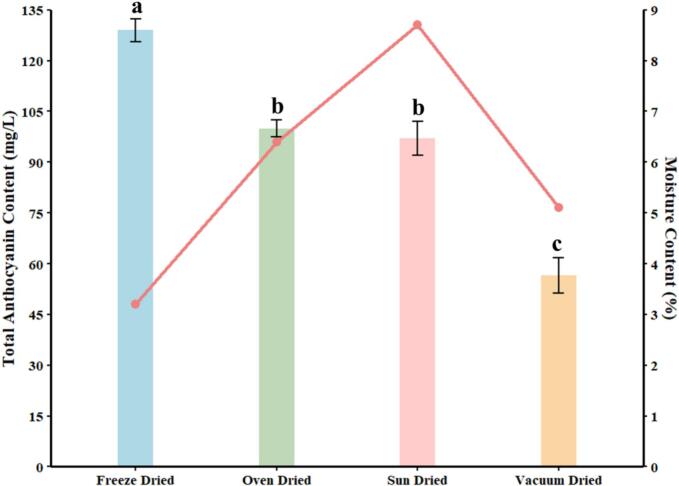
Effect of drying methods on total anthocyanin content and residual moisture in red cabbage. Bar graph represents the total anthocyanin content (mg/L) in red cabbage samples subjected to four different drying methods: Freeze drying, Oven drying, Sun drying, and Vacuum drying. Error bars indicate standard deviation (n = 3). The superimposed red line with circular markers corresponds to the residual moisture content (%) of the respective dried samples, plotted on the secondary Y-axis (right).

Drying is a crucial post-harvest step that directly affects the stability of thermolabile phytochemicals like anthocyanins [24]. The comparative evaluation of drying methods was conducted as a preliminary optimization step to identify the most suitable pre-processing approach for preserving anthocyanins prior to ultrasound-assisted extraction. As drying can significantly influence the pigment integrity and extraction efficiency of thermolabile compounds, this assessment was intended to ensure that the selected drying method provides a stable and reproducible substrate for subsequent UAE experiments. Among the drying techniques evaluated, freeze drying (FD) exhibited the highest anthocyanin retention (129.02 ± 3.36 mg L^−1^), while vacuum drying showed the lowest (56.46 ± 5.18 mg L^−1^), with oven and sun drying yielding intermediate values (Fig. 1), consistent with McCullum et al. (2024), who reported FD retained 402.58 µg C3G/g DW in Illawarra plum due to sublimation under low temperature and vacuum, minimizing thermal degradation and oxidative stress[25]. This process prevents anthocyanin breakdown by avoiding liquid-phase reactions and enzymatic oxidation, preserving pigment integrity and bioactivity. The non-thermal nature of FD limits Maillard reactions and polymerization, which are key pathways for anthocyanin loss during dehydration [25], [26]. In line with these findings, Liu et al. (2024) reported that hot air drying significantly reduced the anthocyanin yield from *Lycium ruthenicum*, whereas freeze drying preserved structural integrity and enhanced recovery of key anthocyanins such as cyanidin-3-galactoside and delphinidin [27]. Conversely, vacuum drying resulted in the lowest retention (56.46 ± 5.18  mg/L), due to moderate heat exposure combined with potential oxygen infiltration, which accelerates anthocyanin breakdown. The absence of rapid vitrification and immediate matrix solidification in vacuum drying increases pigment mobility and oxygen diffusion, promoting oxidation and polymerization processes that diminish anthocyanin retention [28]. Oven drying (99.96 ± 2.57  mg/L) and sun drying (97.09 ± 4.99  mg/L) showed intermediate values and were statistically similar (group ‘b’), although greater variability in sun drying may stem from uncontrolled environmental conditions (Fig. 1). One-way ANOVA confirmed a highly significant effect of drying method on TAC (F(3,8) = 148.6, p = 2.36 × 10^−7^), and Tukey’s HSD test classified the methods into three distinct groups: freeze drying (a), oven/sun drying (b), and vacuum drying (c). Several studies have reported a temperature-dependent decline in anthocyanin content, underscoring their thermal sensitivity. Liu et al. (2018) [29] demonstrated that increasing the temperature to 60 °C triggered chalcone formation, resulting in noticeable color fading and pigment degradation. Jampani and Raghavarao [30] reported a 23 % reduction in red cabbage anthocyanins at 80 °C, highlighting significant thermal degradation at elevated temperatures. Similarly, Vega-Galvez reported consistent degradation in anthocyanins of red cabbage during hot-air drying across 50–90 °C, with the lowest loss (∼15 %) at 50 °C (p < 0.05), and approximately 40 % degradation at 70, 80, and 90 °C, with no significant differences among these higher temperatures (p > 0.05)[31]. These results demonstrated that choosing non-thermal or low-temperature dehydration techniques for pigment-sensitive materials like red cabbage is essential for maintaining their functional and colorant properties.

### 3.2 Optimization of extraction parameters using response surface methodology

**Fig. 2:**
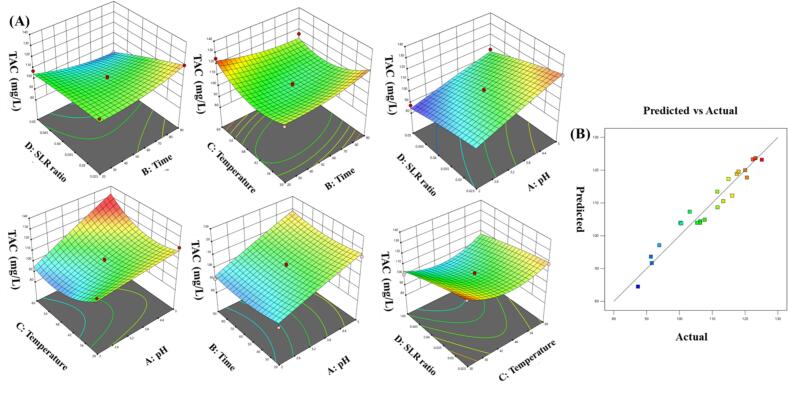
Optimization of ultrasound-assisted extraction parameters for total anthocyanin content using response surface methodology (RSM). **(A)** Response surface plots showing the interactive effects of pH, extraction time, temperature, and solid-to-liquid ratio on total anthocyanin content (TAC) during ultrasound-assisted extraction. **(B)** Predicted vs. actual values plot demonstrating the accuracy of the RSM model.

Efficient recovery of anthocyanins from plant matrices relies on the precise interaction of physicochemical factors that influence solubility and thermal stability. To maximize anthocyanin yield from freeze-dried red cabbage, a Box–Behnken design (BBD) was used with Response Surface Methodology (RSM) to evaluate four key variables: pH (3.0–5.0), temperature (30–60 °C), time (20–90  min), and sample-to-solvent ratio (0.025–0.05  g/mL). The quadratic model, derived from 29 experimental runs, was statistically significant (p < 0.001) with high R2 and adjusted R2 values, and showed no significant lack-of-fit, indicating the model is reliable (Fig. 2A & B). Among the variables, extraction pH and temperature had the most significant linear and quadratic effects, and an important interaction between pH and temperature was also observed. Response surface plots showed that moderate acidity and intermediate temperatures improved anthocyanin recovery, likely because of better pigment solubility at low pH and reduced thermal degradation at moderate heat (Fig. 2A). Numerical optimization indicated that maximum TAC (125.10  mg/L) would be achieved at pH 3.5, 45 °C, 55  min, and a sample-to-solvent ratio of 0.0375  g/mL. This condition was experimentally confirmed with consistent TAC results, validating the model's accuracy (Fig. 2B). Results demonstrated the effectiveness of RSM in optimizing extraction processes for heat-sensitive pigments and emphasize the importance of acid conditions in preserving anthocyanins during water-based extraction. Based on the regression analysis of the Box–Behnken design, a second-order polynomial equation was developed to model the effect of extraction pH (X_1_), time (X_2_), temperature (X_3_), and solid-to-liquid ratio (SLR, X_4_) on total anthocyanin content (TAC, mg/L). The model equation in terms of actual (coded) factor levels is as follows:

(6) $$\mathrm{TAC}=208.41-4.81\cdot pH+0.56\cdot Time-5.31\cdot Temperature+392.03\cdot SLR+0.024\cdot \left(pH\times Time\right)+0.345\cdot \left(pH\times Temperature\right)-75.32\cdot \left(pH\times SLR\right)-0.0095\cdot \left(Time\times Temperature\right)-14.01\cdot \left(Time\times SLR\right)+10.25\cdot \left(Temperature\times SLR\right)-0.233\cdot {\mathrm{pH}}^{2}+0.0027\cdot {\mathrm{Time}}^{2}+0.047\cdot {\mathrm{Temperature}}^{2}-6152.55\cdot {\mathrm{SLR}}^{2}$$

### 3.3 Microencapsulation of anthocyanin

Spray drying microencapsulation achieved high encapsulation efficiency (EE) of 96.36 ± 0.80 % and encapsulation yield of 99.40 %. This is attributed from optimized process kinetics (precise temperature control and atomization), minimizing thermal degradation and core diffusion, coupled with inherent molecular compatibility between anthocyanins and the maltodextrin matrix. Maltodextrin facilitates effective entrapment through hydrogen bonding with phenolic groups and rapid vitrification during drying, forming a protective amorphous barrier that limits anthocyanin exposure to oxygen, heat, and pH fluctuations. The obtained encapsulation efficiency (96.36 ± 0.80 %) lies within the upper range reported for optimized spray-dried anthocyanin systems, where efficiencies typically range from 70 to 95 % and seldom exceed 95 % even with binary carrier matrices [21], [32], [33], [34], [35]. The high yield further reflects negligible operational losses and efficient mass recovery. These results align with the literature [36], [37], [38] on spray-dried anthocyanins and underscore the dual role of carrier-material selection (exploiting maltodextrin’s film-forming, low-viscosity, and oxygen-barrier properties) and process optimization in achieving robust encapsulation for labile phytochemicals. The storage stability of the encapsulated red cabbage colorant was evaluated under different environmental conditions, including temperature (4 °C, 25 °C, and 37 °C), light exposure (amber and non-amber), and oxygen availability (Supplementary file S1, Fig. S1). Total anthocyanin content (TAC) was monitored over eight weeks, revealing gradual pigment degradation influenced by temperature and light intensity. Samples stored at 4 °C under amber light exhibited the highest retention of TAC, while those exposed to elevated temperatures and light showed comparatively higher degradation rates. Overall, the results confirm that the encapsulated matrix provided substantial protection against thermal, photo-oxidative, and environmental degradation, highlighting its effectiveness in enhancing the shelf-life stability of the colorant.

### 3.4 Untargeted LC–MS profiling of red cabbage extracts

Untargeted LC–MS analysis was performed to compare the phenolic and anthocyanin composition of extracts obtained by conventional maceration (S1) and ultrasound-assisted extraction (S2). Compound annotations were performed via KEGG and library matching (Supplementary Tables S2 & S3). In total, 28 metabolites were detected in S1 and 35 in S2, with 20 shared across both extracts. Major molecular classes included flavonoids (e.g., rutin, quercetin derivatives), anthocyanidins (cyanidin, peonidin forms), phenylpropanoids (p-coumaric and ferulic acid derivatives), and coumarin-type compounds. Several glycosylated and conjugated phenolics (e.g., isoorientin rhamnoside, (+)-epicatechin-3-O-gallate, glucuronolactone) were uniquely detected in S2, indicating enrichment of phenolic diversity under ultrasound. Crucially, the acylated anthocyanin markers characteristic of red cabbage, including cyanidin-3-(sinapoyl/feruloyl)-glucosides, exhibited comparable *m*/*z* and retention patterns in both S1 and S2, demonstrating that no structural degradation or deacylation occurred during sonication. This suggests that the moderate ultrasound conditions applied enhanced cell disruption and mass transfer without inducing cavitation-driven oxidative breakdown, which is known to occur only under high intensities or prolonged exposure. The broader phenolic profile in S2 therefore reflects greater extraction of bound glycosides and phenylpropanoid conjugates, consistent with ultrasound-mediated microstructural rupture rather than chemical alteration. Overall, the LC–MS data confirm that ultrasound-assisted extraction increases phenolic diversity and extraction efficiency while preserving the structural integrity of acylated anthocyanins, supporting its suitability for food-grade pigment recovery.

### 3.5 Surface morphology of colorants (encapsulated and non-encapsulated)

**Fig. 3:**
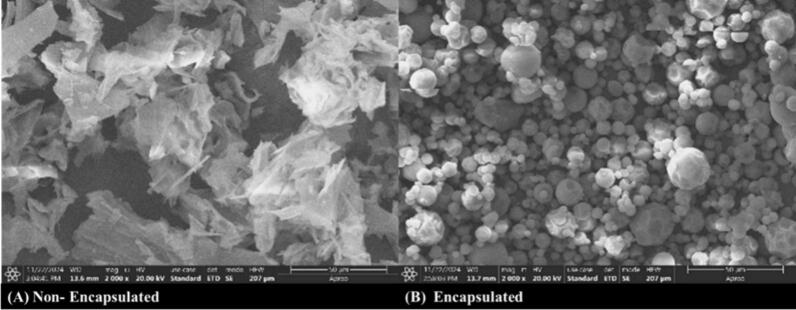
Scanning electron microscopy (SEM) images of powder colorant illustrating the surface morphology and structural characteristics of the particles **(A)** Non-encapsulated **(B)** Encapsulated.

Surface morphological analysis of the anthocyanin-loaded microcapsules revealed predominantly spherical particles with a mean diameter of 10.27 ± 2.8  µm, indicating a consistent size distribution and atomization during spray drying (Fig. 3A & B). The smooth and uniform surface of most microparticles, absence of surface cracks, reflects effective encapsulation and structural integrity, favorable traits for improving stability and shelf life of anthocyanin-rich ingredients. A few particles exhibited surface depressions, folds, or irregular shapes, which are commonly associated with rapid moisture evaporation and matrix shrinkage during the drying and cooling phases. Such morphological patterns are consistent with previous observations in polysaccharide-based microencapsulation systems, where surface wrinkling and dimpling are attributed to the viscoelastic properties and drying kinetics of wall materials [39]. Additionally, the variability in particle shape influenced by the molecular weight and bulk density of maltodextrin, which affects film formation and collapse behavior during drying [40]. The structural uniformity and absence of porosity or rupture further highlight the protective efficacy of maltodextrin, supporting its continued use as a GRAS (Generally Recognized As Safe) wall material for phenolic encapsulation in functional food applications.

### 3.6 Structural characterization by fourier transform infrared spectroscopy (FTIR)

**Fig. 4:**
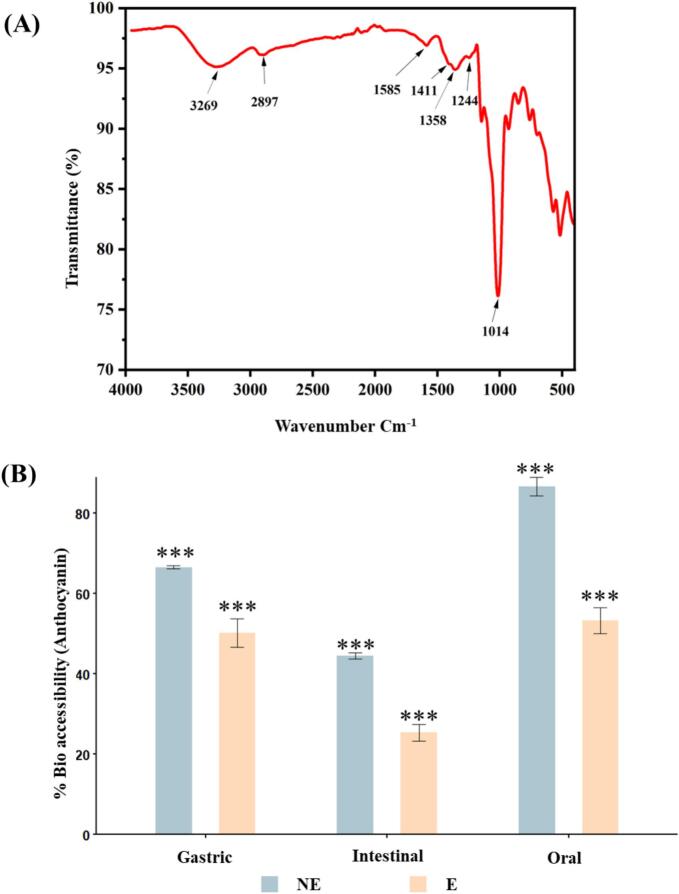
**(A)** FTIR spectrum of encapsulated anthocyanin showing characteristic peaks associated with functional groups and wall material interaction. **(B)** Bioaccessibility of anthocyanins in non-encapsulated (NE) and encapsulated (E) forms across simulated gastric, intestinal, and oral digestion phases.

The FTIR spectra of the encapsulated anthocyanin-rich red cabbage powder confirmed the structural presence and integrity of key functional groups associated with both the anthocyanin pigments and the maltodextrin matrix. A broad absorption band centered around 3269  cm^−1^ was attributed to O–H stretching vibrations, indicative of hydrogen-bonded hydroxyl groups in phenolic compounds, water molecules, and alcohols (fig 4A). It aligns with the spectra reported by Pereira et al. (2015) in anthocyanin-rich extracts and reflects the hydrophilic nature of both the core and wall components [41]. A distinct band observed at 2897  cm^−^1 corresponds to C–H stretching, commonly associated with methyl and methylene groups, further confirming the presence of organic compounds within the encapsulated matrix. The band at 1585  cm^−^1 reflects C=C stretching of aromatic rings, characteristic of the anthocyanin chromophore, while the region between 1411–1358  cm^−^1 was associated with aromatic skeletal vibrations, reinforcing the presence of polyphenolic structures. Comparative analysis of free colorant (NEN) and encapsulant colorant (EN) spectra revealed shifts and intensity variations indicative of non-covalent molecular interactions (e.g., hydrogen bonding) during encapsulation (Table 3, Fig. 4 overlaid). The O-H stretching band shifted from 3240 cm^−^1 in NEN to 3269 cm^−^1 (and broader extension to 3455 cm^−^1) in EN, suggesting disruption of self-hydrogen bonding in free anthocyanins and formation of new bonds with maltodextrin hydroxyls. Aliphatic C-H stretching emerged prominently at 2935 cm^−^1 in EN (absent/weak in NEN), attributable to maltodextrin's CH_2_/CH_3_ groups. Aromatic C=C stretching shifted from 1518 cm^−^1 (NEN) to 1562–1585 cm^−^1 (EN), implying ring distortion or conjugation changes due to steric/electronic effects from the matrix. The C-O region showed marked intensification in EN (95–97 %T at 1030 cm^−^1 vs ∼ 99 %T in NEN), highlighting enhanced polysaccharide contributions and potential new ether-like linkages at the core-wall interface (Supplementary File S1, Fig. S2). The spectral fingerprint obtained in this study closely resembles previously reported anthocyanin FTIR profiles [39], substantiating the structural retention of anthocyanins post-encapsulation.

### 3.7 Color analysis

The colorimetric analysis of the encapsulated anthocyanin-based colorant revealed a visually appealing profile characterized by moderate brightness and a distinct purplish-red hue (Table 1). The L* value of 46.45 ± 0.02 indicates intermediate lightness, while the a* value of 14.25 ± 0.19 confirms a strong reddish component. A negative b* value of –7.88 ± 0.04 suggests the presence of blue undertones, collectively contributing to a purplish appearance. The hue angle (h*) of 298.24° corresponds to a characteristic purplish-red hue typical of anthocyanin pigments. Furthermore, the chroma (C*ab) value of 16.28 denotes moderate color intensity, while the saturation index (S*ab) of 33.08 % reflects a moderately saturated color influenced by the overall lightness. Comparative studies confirm similar values for L*, a*, and b* in encapsulated systems from other sources, with spray drying and maltodextrin consistently improving color stability and application potential [42], [43], [44]. However, all methods show reduced vividness and color retention after light exposure, marking a limitation[44]. Overall, the current method demonstrates competitive advantages in color saturation and thermal stability, but modest resistance to photodegradation. These parameters indicate that the encapsulated colorant retains the desirable visual qualities of anthocyanins, making it suitable for application in food and nutraceutical formulations where color appeal is critical.

**Table 1:** Color analysis of encapsulated red cabbage powder.

**Parameter**	**Value**	**Interpretation**
L* (Lightness)	46.45 ± 0.02	Moderate brightness.
a* (Red-Green)	14.25 ± 0.19	Positive value indicates a reddish hue.
b* (Yellow-Blue)	−7.88 ± 0.04	Negative value indicates a bluish tone.
Hue Angle (h*)	298.24°	Corresponds to a purplish-red hue.
Chroma (C*ab)	16.28	Moderate color intensity.
Saturation Index (S*ab)	33.08 %	Indicates moderate saturation, influenced by lightness.

### 3.8 Impact of microencapsulation on phytochemical retention and antioxidant capacity

The encapsulation of anthocyanin-rich red cabbage extract was found to significantly affect the retention of phytochemicals and their antioxidant capacity (Supplementary file S1, Table S1). Quantitative analysis showed that the non-encapsulated extract had notably higher levels of total anthocyanins (125.72 ± 3.79  mg/L), total phenolics (437.56 ± 49.52  mg GAE/g), flavonoids (5.16 ± 0.38  mg RE/g), and flavonols (4.44 ± 0.52  mg RE/g) compared to the encapsulated version, which retained 83.74 ± 0.89  mg/L, 213.44 ± 18.78  mg GAE/g, 2.84 ± 0.06  mg RE/g, and 2.14 ± 1.58  mg RE/g, respectively (Table 2). A similar decrease was seen in antioxidant activity, with DPPH scavenging capacity dropping from 84.00 ± 0.87 % in the free extract to 66.90 ± 4.30 % after encapsulation. These differences can stem from process-induced degradation and the barrier effect of the encapsulation matrix. Thermal and oxidative stress during spray drying partially degrade sensitive polyphenolics [45], [46], [47], while the encapsulation matrix responsible for protection and controlled release, can limit solvent access during in vitro tests, leading to lower measured content. Despite this decrease, the encapsulated extract still retained over 66 % of its original antioxidant activity and a significant portion of its phytochemicals, highlighting the effectiveness of maltodextrin as a protective carrier. Additionally, the observed reduction should be viewed within the context of intended functional stability rather than immediate extractability. Encapsulation not only preserves anthocyanins during processing and storage but also facilitates targeted delivery under physiological conditions. Therefore, the reported retention rates demonstrate a good balance between stability and bioactivity, supporting the potential use of the encapsulated powder as a functional ingredient in health-focused formulations where shelf life and controlled release are critical.

**Table 2:** Phytochemical analysis and antioxidant potential of Non-Encapsulated (NE) and Encapsulated Red cabbage (RC) powder.

**Parameters**	**RC (NE)**	**RC(E)**
**Total Anthocyanin Content (CGE mg/L)**	125.72 ± 3.79	83.74 ± 0.89
**Total Phenolic Content (mg GAE eq/g)**	437.56 ± 49.52	213.44 ± 18.78
**Total Flavonoid Content (mg Rutin eq/g)**	5.16 ± 0.38	2.84 ± 0.06
**Total Flavonols Content (mg Rutin eq/g)**	4.44 ± 0.52	2.14 ± 1.58
**Antioxidant by DPPH (%)**	84 ± 0.87	66.90 ± 4.30
**FRAP (eq µM Fe + 2/L of sample)**	4.69 ± 1.22	4.59 ± 0.45
**ABTS (% Inhibition)**	95.44 ± 0.30	71.76 ± 0.44 %

The values are the means ± standard deviations (SD) of triplicate repeated sets.

### 3.9 Thermal stability and degradation kinetics of encapsulated anthocyanins

Thermal degradation is a major challenge for anthocyanin-based colorants, as elevated temperatures often compromise pigment stability and visual appeal. Three temperature levels (70 °C, 90 °C, and 120 °C) were selected to represent typical food-processing conditions where anthocyanin-based colorants may be applied. Specifically, 70 °C corresponds to mild pasteurization used in yogurt formulation, 90 °C reflects moderate heating during beverage or sauce processing, and 120 °C simulates high-temperature treatments such as extrusion or brief baking. Non-encapsulated extracts showed pronounced thermal sensitivity, with retention declining from 93.24 % at 70 °C (30  min) to 50.21 % at 120 °C (60  min). In contrast, encapsulated samples retained 98.72 % at 70 °C and 65.77 % at 120 °C under identical conditions, indicating substantial protection afforded by the maltodextrin matrix (Table 3). Kinetic analysis confirmed that anthocyanin degradation followed a first-order reaction model, with degradation rate constants (*k*) increasing and half-lives (*t*_1_*/*_2_) decreasing at elevated temperatures. Non-encapsulated samples showed rapid degradation, with *k* values ranging from 0.00233 to 0.01150  min^−1^ and corresponding *t*_1_*/*_2_ values between 300.03 and 60.58  min. In comparison, encapsulated samples demonstrated significantly lower *k* values (0.00043–0.00698  min^−1^) and longer *t*_1_*/*_2_ values (1919.02–99.25  min), emphasizing the stabilizing effect of encapsulation (Table 3). These findings align with prior studies suggesting that polysaccharide-based matrices reduce anthocyanin degradation by limiting thermal diffusion and oxidative exposure [48]. The protective mechanism may further benefit from internal co-pigmentation, flavonoid–metal ion complexation, and acidic pH-induced stabilization of the flavylium cation [49]. Although extreme thermal stress can trigger hydrolytic cleavage and ester bond degradation [50], encapsulated systems demonstrated resilience under moderate heat. This is further supported by Sendri et al. (2023), who reported partial anthocyanin retention even at 160 °C [51]. Overall, the results substantiate encapsulation as an effective strategy to mitigate thermal degradation kinetics, thereby enhancing the functional lifespan of anthocyanin-rich formulations in heat-processed applications.

**Table 3:** Anthocyanin degradation rate constant (k value) and half-life estimation with elevated temperature for thermal stability test.

**Sample**	**Temp (°C)**	**k(30 min)**	**t(1/2)**	**k(60 min)**	**t(1/2)**
**Non-Encapsulated**	70	0.0023	300.0322	0.0040	181.0617
	80	0.0027	253.0931	0.0057	122.0096
	90	0.0030	233.0772	0.0069	101.3344
	100	0.0031	220.3671	0.0071	98.4861
	110	0.0036	191.8985	0.0090	77.3343
	120	0.0060	116.8733	0.0115	60.5778
**Encapsulated**	70	0.0004	1919.0160	0.0036	190.7183
	80	0.0008	933.3109	0.0044	158.5232
	90	0.0016	450.6237	0.0049	142.3406
	100	0.0027	256.9647	0.0059	117.2586
	110	0.0033	210.6888	0.0063	109.7106
	120	0.0052	132.8175	0.0070	99.2544

### 3.10 In vitro bioaccessibility of encapsulated and non-encapsulated anthocyanins

Due to their structural instability under physiological conditions, anthocyanins are widely recognized for their limited bioavailability. In this study, simulated gastrointestinal digestion revealed clear differences in release and retention patterns between non-encapsulated and encapsulated red cabbage anthocyanins. During the oral phase, non-encapsulated extracts showed significantly higher bioaccessibility (86.67 ± 2.27 %) than encapsulated ones (53.28 ± 3.19 %), indicating rapid pigment release into the buccal environment (fig 4B). This immediate release likely results from direct solubilization in aqueous saliva without a carrier matrix. However, as digestion continued, encapsulated anthocyanins demonstrated greater stability and controlled release. In the gastric phase, while bioaccessibility of non-encapsulated anthocyanins dropped to 66.53 ± 0.35 %, encapsulated forms maintained 50.15 ± 3.55 %, highlighting maltodextrin’s protective effect in acidic environments. A significant decline occurred in the intestinal phase for both samples, due to anthocyanin instability at neutral to alkaline pH, where they transform into non-colored chalcone, quinoidal base, and hemiketal forms, as documented [52], [53]. Despite this, encapsulated anthocyanins still retained 25.37 ± 2.09 %, lower than non-encapsulated samples (44.50 ± 0.77 %), indicating delayed release rather than breakdown. These results align with previous research [48], [54] which showed that maltodextrin-based microencapsulation protects against enzymatic degradation and pH-triggered structural changes during digestion. While non-encapsulated extracts offer higher initial bioaccessibility, encapsulation allows for gradual, pH-responsive release, supporting the development of targeted delivery systems with extended functionality. This dual behavior highlights the trade-off between immediate bioavailability and digestive resilience, emphasizing the strategic importance of encapsulation in stabilizing and delivering anthocyanins.

### 3.11 Anti-diabetic activity

Inhibition of carbohydrate-hydrolyzing enzymes such as α-amylase and α-glucosidase represents a key therapeutic target for attenuating postprandial glucose spikes in type-II diabetes. The present study assessed the inhibitory potential of red cabbage extracts in both encapsulated (RC-E) and non-encapsulated (RC-NE) forms before and after simulated gastrointestinal digestion. The non-encapsulated extract demonstrated superior α-amylase inhibition, with an IC_50_ of 21.87 ± 0.03  μg/mL, compared to 46.00 ± 5.43  μg/mL for RC-E. Post-digestion, RC-E showed a modest reduction in activity (*IC*_50_ *= 49.65 ±* 3.30  μ*g/mL*), whereas the pharmaceutical reference standard acarbose exhibited an IC_50_ of 44.26 ± 2.71  μg/mL, situating the encapsulated extract within a comparable range. In contrast, α-glucosidase inhibition patterns differed, with RC-E showing stronger inhibition before digestion (*IC*_50_ *= 51.51 ±* 2.07  μ*g/mL*) than RC-NE (*74.38 ±* 3.24  μ*g/mL*), suggesting that encapsulation initially enhanced the extract’s enzyme-targeting efficacy. However, following digestion, RC-E exhibited a substantial loss in inhibitory capacity (*IC*_50_ *= 154.71 ±* 27.91  μ*g/mL*), indicating possible structural degradation or bioactive sequestration during gastrointestinal transit. These trends may reflect the differential stability of anthocyanins and other polyphenolics during digestion, and their interactions with digestive enzymes or the encapsulation matrix. The enzyme inhibition results are consistent with previous findings showing anthocyanin subclasses such as delphinidin and cyanidin glucosides exhibit notable α-glucosidase and α-amylase inhibitory activity [55], [56]. Additionally, synergistic effects among polyphenols, including flavonols and phenolic acids, have been reported to modulate carbohydrate metabolism enzymes[57]. The observed reduction in activity post-digestion emphasizes the need for optimized encapsulation systems to sustain bioactivity across gastrointestinal conditions. These results position red cabbage extract, particularly in its non-encapsulated state, as a promising natural enzyme inhibitor while underscoring the importance of delivery strategies to maintain efficacy under physiological stress.

### 3.12 Cytotoxicity evaluation of colorant

**Fig. 5:**
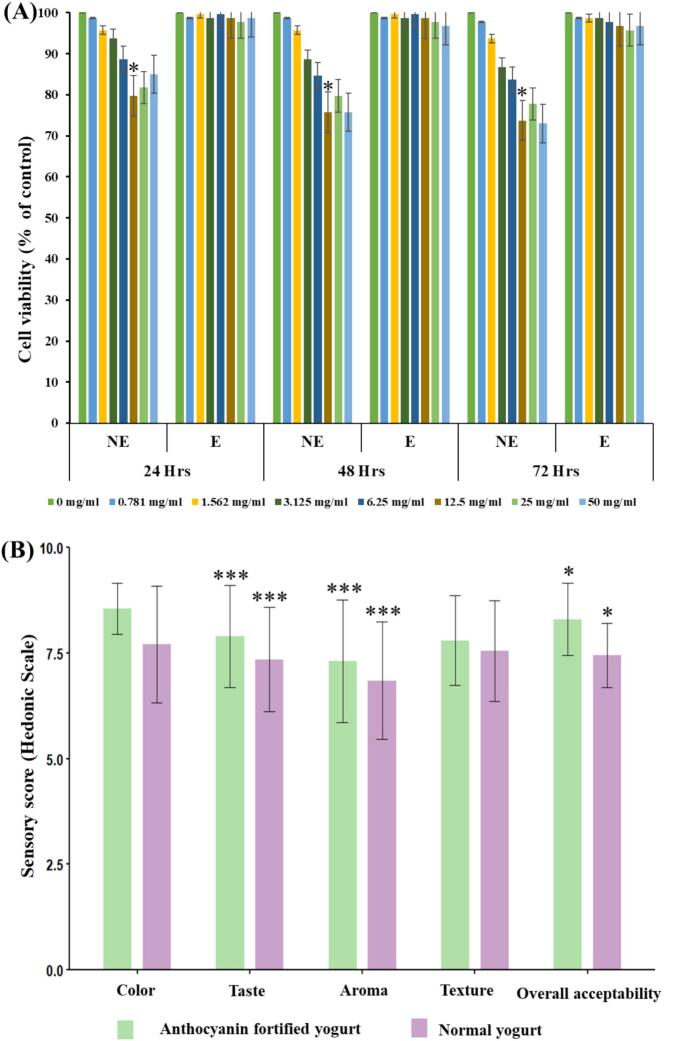
**(A)** Cytotoxicity of encapsulated and non-encapsulated anthocyanin formulations assessed in HEK-293 cells at various concentrations (10–100 µg/mL) using MTT assay. Results are expressed as percentage cell viability (mean ± SD, n = 3), indicating > 90 % viability for both forms across all doses. **(B)** Sensory analysis of normal and anthocyanin fortified yogurt.

The cytotoxicity evaluation of colorants using the MTT assay on HEK-293 cells revealed a dose-dependent response in cell viability across different formulations. As shown in the fig. 5A, all tested concentrations (ranging from 25 to 200 µg/mL) of both encapsulated and non-encapsulated colorants-maintained cell viability above 75 %, indicating good cytocompatibility. Notably, encapsulated samples consistently exhibited higher viability than their non-encapsulated counterparts, especially at elevated concentrations, suggesting that encapsulation mitigates potential cytotoxic effects. The encapsulated colorant showed the highest biocompatibility among the samples, maintaining cell viability above 90 % across all tested doses. These results support the safety of the anthocyanin-based colorants for potential application in food and biomedical products in consistent with previous report of no potential cytotoxic effect of red cabbage extract [31].

### 3.13 Sensory evaluation of anthocyanin-fortified yoghurt

Anthocyanin-fortified yoghurt exhibited superior sensory performance compared to the control across all evaluated attributes. Anthocyanin-fortified yoghurt exhibited superior sensory attributes compared to the control, with significant improvements in taste (F = 15.41, p < 0.001), aroma (F = 13.42, p < 0.001), and overall acceptability (F = 7.27, p = 0.010). The enhancement in color score (8.55 ± 0.60 vs. 7.70 ± 1.38) further contributed to visual appeal and consumer preference (Supplementary file S1, Table S2), although the difference was not statistically significant (p > 0.05). Texture remained non-significant, indicating that anthocyanin incorporation did not adversely influence the structural integrity or mouthfeel of the product. Despite the close numerical values across treatments, the statistical outcomes confirmed perceptible and consistent sensory improvements, emphasizing the functional compatibility of encapsulated anthocyanins in fermented dairy matrices and their potential to enhance consumer acceptance in functional food applications (Fig. 5B).

### 3.14 Storage stability of anthocyanin-fortified yoghurt

**Fig. 6:**
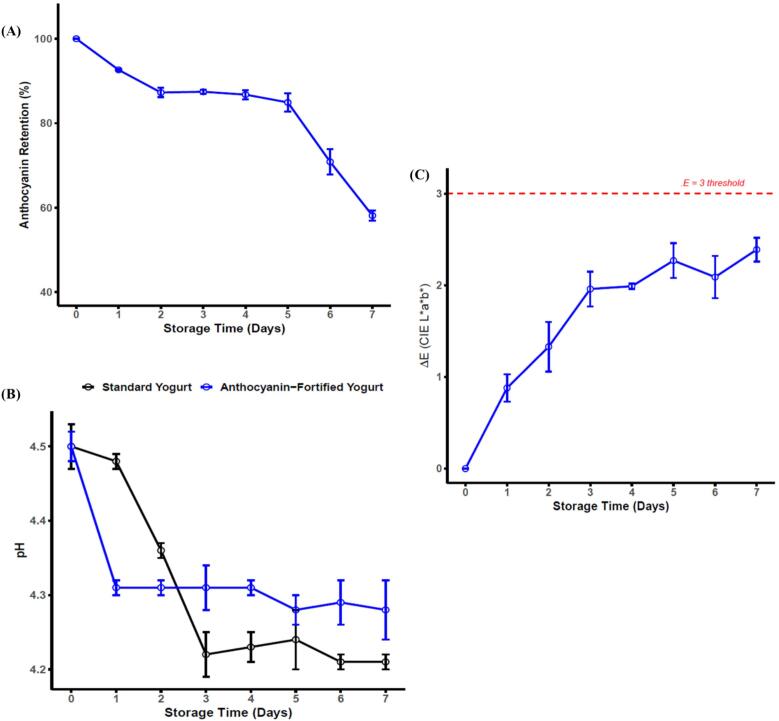
Stability and physicochemical properties of anthocyanin-fortified yogurt during refrigerated storage (4 °C) for 7 days. **(A)** Anthocyanin retention (%) showing gradual pigment degradation over time. **(B)** Variation in pH of standard and anthocyanin-fortified yogurts. **(C)** Total color difference (ΔE, CIEL*a*b*) illustrating perceptible color change relative to day 0; the dashed red line represents the ΔE = 3 threshold for visual detectability.

The storage study demonstrated that the anthocyanin-fortified yogurt maintained chemical and visual stability over the typical consumer shelf-life period (one week). The gradual decrease in TAC from Day 0 to Day 5 (∼15 % loss) followed by a steeper decline after Day 6 aligns with the known biphasic anthocyanin degradation kinetics in protein-fermentate systems (Fig. 6A), where early stability reflects physical entrapment within the casein-whey protein network, while later-stage decline is driven by progressive acid-mediated ring-opening and subsequent oxidative cleavage[58]. The maintenance of ΔE values below the sensory threshold (ΔE < 3) through Day 5 indicates that this molecular loss did not translate to perceptible discoloration, confirming that encapsulation delayed chromophore damage and preserved the flavylium cation–dominated absorbance profile responsible for red–purple pigmentation.

Simultaneously, the pH of the fortified yogurt followed a nearly identical trajectory to the control, decreasing from ∼ 4.50 to ∼ 4.28 during storage (Fig. 6B). This demonstrates that anthocyanin incorporation did not interfere with lactic acid fermentation or post-acidification, indicating no metabolic inhibition of starter cultures nor buffering effects attributable to the pigment matrix. The small, gradual decline in pH is consistent with controlled residual fermentation and moisture equilibrium typical of commercial yogurt systems, confirming matrix compatibility and fermentation stability. Taken together, the convergence of high TAC retention through Day 5, sub-threshold ΔE values indicating stable visual quality, and unchanged acidification behavior provides strong evidence that microencapsulated anthocyanins are structurally and functionally stable in fermented dairy environments (Fig. 6C). These results directly support the technological feasibility of deploying spray-dried encapsulated anthocyanins as natural colorants in refrigerated dairy products, without compromising fermentation performance, visual appeal and short-term storage quality.

## 4 Conclusion

This study presents a comprehensive, process-driven approach to enhancing the functional utility of red cabbage anthocyanins through ultrasound-assisted extraction and spray-drying microencapsulation, positioning them as promising candidates for next-generation functional food colorants. Optimization using response surface methodology yielded a high anthocyanin recovery of 125.72 ± 0.83  mg/L under eco-friendly, green extraction conditions. Microencapsulation with maltodextrin achieved exceptional encapsulation efficiency (96.36 ± 0.80 %) and significantly improved thermal stability, with extended half-lives up to 1919.02  min and reduced degradation rates (k = 0.00043–0.0070  min^−1^). Encapsulated formulations retained > 90 % cell viability in cytotoxicity assays, exhibited robust antioxidant capacity (DPPH: 66.9 %; ABTS: 71.76 %), and showed enzyme inhibitory activities relevant to glycemic control (α-glucosidase IC_50_: 51.51  µg/mL). Simulated digestion studies confirmed enhanced gastric stability and a controlled-release profile, supporting improved bioaccessibility and physiological relevance. These outcomes highlight the synergistic role of encapsulation in preserving anthocyanin bioactivity, structural integrity, and functional performance under gastrointestinal and processing stresses. Future research should focus on in vivo validation of metabolic and antioxidative benefits, matrix compatibility in real food systems, and regulatory standardization to enable commercial translation of red cabbage anthocyanins as stable, safe, and health-promoting bioactive ingredients in functional food and nutraceutical applications.

## CRediT authorship contribution statement

**Sumandeep Kaur:** Writing – original draft, Data curation. **Amisha Rani:** Writing – review & editing, Investigation, Formal analysis. **Reshma Thakur:** Data curation. **Abhilasha Sharma:** Data curation. **Kritika Kuksal:** Data curation. **Aman Sharma:** Data curation. **Arti Shivraj Nile:** Validation, Supervision. **Shivraj Hariram Nile:** .

## Declaration of competing interest

The authors declare that they have no known competing financial interests or personal relationships that could have appeared to influence the work reported in this paper.

## References

[1] Belwal T., Singh G., Jeandet P., Pandey A., Giri L., Ramola S., Bhatt I.D., Venskutonis P.R., Georgiev M.I., Clément C., Luo Z. (2020). Anthocyanins, multi-functional natural products of industrial relevance: recent biotechnological advances. Biotechnol. Adv..

[2] Khalafi N., Gharachorloo M., Ganjloo A., Yousefi S. (2024). Release kinetics, color stability and antioxidant activity of red cabbage anthocyanins encapsulated in zein electrospun nanoribbons. J. Food Meas. Charact..

[3] Oliveira J., Benvenutti L., Albuquerque B.R., Finimundy T.C., Mandim F., Pires T.C.S.P., Pereira C., Corrêa R.C.G., Barros L., Zielinski A.A.F. (2025). Green extraction of anthocyanin from red cabbage waste using acid whey as a promising bio-based solvent. Innovative Food Sci. Emerg. Technol..

[4] Hwangbo H., Cha H.-J., Park C., Jeong H.J., Moon S.-K., Yun S.J., Kim W.-J., Noh J.S., Kim H.-S., Shim J.-H., Kim G.-Y., Choi Y.H. (2025). Anthocyanins extracted from Vitis coignetiae pulliat fruits induce reactive oxygen species-dependent growth arrest and apoptosis in PC3 human prostate carcinoma cells. Biotechnol. Bioprocess Eng..

[5] Gürses A., Açıkyıldız M., Güneş K., Gürses M.S. (2016:). Colorants in Health and Environmental Aspects. In.

[6] Kaur D., Yousuf B., Qadri O.S. (2024). Syzygium cumini anthocyanins: recent advances in biological activities, extraction, stability, characterisation and utilisation in food systems. Food Prod. Process. Nutr..

[7] Fenger J.-A., Moloney M., Robbins R.J., Collins T.M., Dangles O. (2019). The influence of acylation, metal binding and natural antioxidants on the thermal stability of red cabbage anthocyanins in neutral solution. Food Funct..

[8] Liu Y., Zhang Y., Zhou Y., Feng X. (2024). Anthocyanins in Different Food Matrices: recent Updates on Extraction, Purification and Analysis Techniques. Crit. Rev. Anal. Chem..

[9] Córdova A., Catalán S., Carrasco V., Farias F.O., Trentin J., López J., Salazar F., Mussagy C.U. (2025). Sustainable assessment of ultrasound-assisted extraction of anthocyanins with bio-based solvents for upgrading grape pomace Cabernet Sauvignon derived from a winemaking process. Ultrason. Sonochem..

[10] Ahmed T., Rana M.R., Hossain M.A., Ullah S., Suzauddula M. (2024). Optimization of ultrasound-assisted extraction using response surface methodology for total anthocyanin content, total phenolic content, and antioxidant activities of Roselle (Hibiscus sabdariffa L.) calyces and comparison with conventional Soxhlet extraction. Biomass Convers. Biorefin..

[11] Nurkhasanah A., Fardad T., Carrera C., Setyaningsih W., Palma M. (2023). Ultrasound-Assisted Anthocyanins Extraction from Pigmented Corn: Optimization using Response Surface Methodology. Methods Protoc.

[12] Catalkaya G., Guldiken B., Capanoglu E. (2022). Encapsulation of anthocyanin-rich extract from black chokeberry (*Aronia melanocarpa*) pomace by spray drying using different coating materials. Food Funct..

[13] J.T. do P. Silva, M.H. Borges, C.A.C. de Souza, C.S. Fávaro-Trindade, P.J. do A. Sobral, A.L. de Oliveira, M. Martelli-Tosi, (2024). Grape Pomace Rich-Phenolics and Anthocyanins Extract: production by Pressurized Liquid Extraction in Intermittent Process and Encapsulation by Spray-Drying. Foods.

[14] Sakulnarmrat K., Sittiwong W., Konczak I. (2022). Encapsulation of mangosteen pericarp anthocyanin‐rich extract by spray drying. Int. J. Food Sci. Technol..

[15] Jafari S., Jafari S.M., Ebrahimi M., Kijpatanasilp I., Assatarakul K. (2023). A decade overview and prospect of spray drying encapsulation of bioactives from fruit products: Characterization, food application and in vitro gastrointestinal digestion. Food Hydrocoll..

[16] Soiklom S., Siri-anusornsak W., Petchpoung K., Kansandee W. (2024). Development of Anthocyanin-Rich Gel Beads from Colored Rice for Encapsulation and In Vitro Gastrointestinal Digestion. Molecules.

[17] Wu Y., Han Y., Tao Y., Li D., Xie G., Show P.L., Lee S.Y. (2020). In vitro gastrointestinal digestion and fecal fermentation reveal the effect of different encapsulation materials on the release, degradation and modulation of gut microbiota of blueberry anthocyanin extract. Food Res. Int..

[18] Liang Y., Li Y., Zhang L., Liu X. (2019). Phytochemicals and antioxidant activity in four varieties of head cabbages commonly consumed in China. Food Prod. Process. Nutr..

[19] Podsędek A., Majewska I., Kucharska A.Z. (2017). Inhibitory potential of Red Cabbage against Digestive Enzymes Linked to Obesity and Type 2 Diabetes. J. Agric. Food Chem..

[20] Mercedes B.-F.-A., Santos G.-S.-J., Nydia C.-B.-O., Isabel S.-M.-D., Jaime L.-C., Karina B.-R.-A. (2022). Validation of a micro-assay based on the pH differential method to quantify total monomeric anthocyanins in red cabbage (Brassica oleracea var. capitata f rubra). J. Food Meas. Charact..

[21] Mehta D., Kuksal K., Yadav K., Kumar Yadav S., Zhang Y., S. (2024). Hariram Nile, Ultrasound-assisted extraction and encapsulation of betalain from prickly pear: Process optimization, in-vitro digestive stability, and development of functional gummies. Ultrason Sonochem 108.

[22] Mehta D., Kuksal K., Sharma A., Soni N., Kumari S., Nile S.H. (2025). Postharvest integration of prickly pear betalain-enriched gummies with different sugar substitutes for decoding diabetes type-II and skin resilience - in vitro and in silico study. Food Chem..

[23] Yu M., Zhu S., Huang D., Tao X., Li Y. (2024). Inhibition of starch digestion by phenolic acids with a cinnamic acid backbone: Structural requirements for the inhibition of α-amylase and α-glucosidase. Food Chem..

[24] Mondol M.S.A., Akbar U., Mandal O., Rani A., Dar A.H., Chatterjee A., Abdi G. (2025). Advances in agronomic practices, postharvest technologies, and medicinal potential of dragon fruit (Hylocereus spp.): a comprehensive updated review. J Agric Food Res.

[25] McCullum R., Saifullah M., Bowyer M., Vuong Q.V. (2024). The impact of drying method and temperature on the colour and functional quality of Illawarra plum (Podocarpus elatus). Appl. Food Res..

[26] Mejías N., Vega-Galvez A., Gomez-Perez L.S., Pasten A., Uribe E., Cortés A., Valenzuela-Barra G., Camus J., Delporte C., Bernal G. (2024). Health-Promoting Properties of Processed Red Cabbage (Brassica oleracea var. capitata f. rubra): Effects of Drying Methods on Bio-compound Retention. Foods.

[27] Liu Y., Deng Y., Yang Y., Dong H., Li L., Chen G. (2024). Comparison of different drying pretreatment combined with ultrasonic-assisted enzymolysis extraction of anthocyanins from Lycium ruthenicum Murr. Ultrason Sonochem 107.

[28] Kuck L.S., Noreña C.P.Z. (2016). Microencapsulation of grape (Vitis labrusca var. Bordo) skin phenolic extract using gum Arabic, polydextrose, and partially hydrolyzed guar gum as encapsulating agents. Food Chem..

[29] Liu Y., Tikunov Y., Schouten R.E., Marcelis L.F.M., Visser R.G.F., Bovy A. (2018). Anthocyanin Biosynthesis and Degradation Mechanisms in Solanaceous vegetables: a Review. Front. Chem..

[30] Jampani C., Raghavarao K.S.M.S. (2015). Process integration for purification and concentration of red cabbage (Brassica oleracea L.) anthocyanins. Sep Purif Technol 141.

[31] Vega-Galvez A., Gomez-Perez L.S., Zepeda F., Vidal R.L., Grunenwald F., Mejías N., Pasten A., Araya M., Ah-Hen K.S. (2023). Assessment of Bio-Compounds Content, Antioxidant activity, and Neuroprotective effect of Red Cabbage (Brassica oleracea var. Capitata Rubra) Processed by Convective Drying at Different Temperatures, Antioxidants.

[32] Zahed N., Esmaeilzadeh Kenari R., Farahmandfar R. (2023). Effect of different extraction methods on antioxidant properties and encapsulation efficiency of anthocyanin of pomegranate peel. Food Sci. Nutr..

[33] Mehran M., Masoum S., Memarzadeh M. (2020). Improvement of thermal stability and antioxidant activity of anthocyanins of Echium amoenum petal using maltodextrin/modified starch combination as wall material. Int. J. Biol. Macromol..

[34] da Silva Júnior M.E., Araújo M.V.R.L., Martins A.C.S., dos Santos Lima M., da Silva F.L.H., Converti A., Maciel M.I.S. (2023). Microencapsulation by spray-drying and freeze-drying of extract of phenolic compounds obtained from ciriguela peel. Sci. Rep..

[35] Pauletto F.B., Hentz R., Oro C.E.D., Borgmann C., Camargo S., Dallago R.M., Cansian R.L., Tres M.V., Valduga E., Paroul N. (2025). Extraction and Spray Drying-based Encapsulation of Anthocyanin Pigments from Jabuticaba Sabará Peel (Myrciaria jaboticaba (Vell.). O. Berg), Processes.

[36] Idham Z., Muhamad I.I., Sarmidi M.R. (2012). DEGRADATION KINETICS AND COLOR STABILITY OF SPRAY‐DRIED ENCAPSULATED ANTHOCYANINS FROM *HIBISCUS SABDARIFFA* l. J. Food Process Eng.

[37] Silva-Espinoza M.A., García-Martínez E., Martínez-Navarrete N. (2021). Protective capacity of gum Arabic, maltodextrin, different starches, and fibers on the bioactive compounds and antioxidant activity of an orange puree (Citrus sinensis (L.) Osbeck) against freeze-drying and in vitro digestion. Food Chem 357.

[38] Zanoni F., Primiterra M., Angeli N., Zoccatelli G. (2020). Microencapsulation by spray-drying of polyphenols extracted from red chicory and red cabbage: Effects on stability and color properties. Food Chem..

[39] Machado M.H., A. da R. Almeida, M.V. de O.B. Maciel, V.B. Vitorino, G.C. Bazzo, C.G. da Rosa, W.G. Sganzerla, C. Mendes, P.L.M. Barreto, (2022). Microencapsulation by spray drying of red cabbage anthocyanin-rich extract for the production of a natural food colorant, Biocatal Agric. Biotechnol.

[40] Sendri N., Singh S., Bhatt V., Bhatt P., Bhandari P. (2022). Valorization of red cabbage pomace for stabilization of anthocyanins in Rhododendron arboreum. Ind. Crops Prod..

[41] Pereira V.A., de Arruda I.N.Q., Stefani R. (2015). Active chitosan/PVA films with anthocyanins from Brassica oleraceae (Red Cabbage) as Time–Temperature Indicators for application in intelligent food packaging. Food Hydrocoll..

[42] Priya S.B., Preetha R. (2016). Study on Color Stability and Microencapsulation of Anthrocyanin Pigment using Spray Drying, Biosci Biotechnol Res. Asia.

[43] Kar A., Kumar Mahato D., Singh Patel A., Bal L.M. (2019). The Encapsulation Efficiency and Physicochemical Characteristics of Anthocyanin from Black Carrot (Daucus Carota Ssp. Sativus) as Affected by Encapsulating Materials, Current Agriculture Research Journal 7.

[44] Feitosa B.F., Decker B.L.A., de Brito E.S., Marques M.C., Rodrigues S., Mariutti L.R.B. (2025). Anthocyanins stability theory – evidence summary on the effects of microencapsulation. Food Bioprod. Process..

[45] Waterhouse G.I.N., Sun-Waterhouse D., Su G., Zhao H., Zhao M. (2017). Spray-Drying of Antioxidant-Rich Blueberry Waste Extracts. Interplay between Waste Pretreatments and Spray-Drying Process, Food Bioproc Tech.

[46] Suhag Y., Nanda V. (2016). Optimization for spray drying process parameters of nutritionally rich honey powder using response surface methodology. Cogent Food Agric..

[47] Suhag Y., Nanda V. (2015). Optimisation of process parameters to develop nutritionally rich spray‐dried honey powder with vitamin C content and antioxidant properties. Int. J. Food Sci. Technol..

[48] Righi da Rosa J., Nunes G.L., Motta M.H., Fortes J.P., Cezimbra Weis G.C., Rychecki Hecktheuer L.H., Muller E.I., Ragagnin de Menezes C., Severo da Rosa C. (2019). Microencapsulation of anthocyanin compounds extracted from blueberry (Vaccinium spp.) by spray drying: Characterization, stability and simulated gastrointestinal conditions. Food Hydrocoll..

[49] Wu J., Guan Y., Zhong Q. (2015). Yeast mannoproteins improve thermal stability of anthocyanins at pH 7.0. Food Chem 172.

[50] Fernandes A., Raposo F., Evtuguin D.V., Fonseca F., Ferreira-da-Silva F., Mateus N., Coimbra M.A., de Freitas V. (2021). Grape pectic polysaccharides stabilization of anthocyanins red colour: Mechanistic insights. Carbohydr. Polym..

[51] Sendri N., Singh S., Bhatt S., Gupta M., Bhandari P. (2023). Insight into the influence of oxygen, sunlight and temperature on the stability and color attributes of red cabbage anthocyanins and in vitro gastrointestinal behaviour. Food Chem. Adv..

[52] Ávila S., Zalamanski S., Tanikawa L.M., Kruger C.C.H., Ferreira S.M.R. (2023). Influence of Cooking Methods on In Vitro Bioaccessibility of Phenolics, Flavonoids, and Antioxidant activity of Red Cabbage. Plant Foods Hum. Nutr..

[53] Stafussa A.P., Maciel G.M., Bortolini D.G., Maroldi W.V., Ribeiro V.R., Fachi M.M., Pontarolo R., Bach F., Pedro A.C., Haminiuk C.W.I. (2021). Bioactivity and bioaccessibility of phenolic compounds from Brazilian fruit purees. Future Foods.

[54] Pan L.-H., Chen L.-P., Wu C.-L., Wang J.-F., Luo S.-Z., Luo J.-P., Zheng Z. (2022). Microencapsulation of blueberry anthocyanins by spray drying with soy protein isolates/high methyl pectin combination: Physicochemical properties, release behavior in vitro and storage stability. Food Chem..

[55] Zhang J., Sun L., Dong Y., Fang Z., Nisar T., Zhao T., Wang Z.-C., Guo Y. (2019). Chemical compositions and α-glucosidase inhibitory effects of anthocyanidins from blueberry, blackcurrant and blue honeysuckle fruits. Food Chem..

[56] Cásedas G., Les F., González-Burgos E., Gómez-Serranillos M.P., Smith C., López V. (2019). Cyanidin-3-O-glucoside inhibits different enzymes involved in central nervous system pathologies and type-2 diabetes. S. Afr. J. Bot..

[57] Ghosh S., More P., Derle A., Patil A.B., Markad P., Asok A., Kumbhar N., Shaikh M.L., Ramanamurthy B., Shinde V.S., Dhavale D.D., Chopade B.A. (2014). Diosgenin from Dioscorea bulbifera: Novel hit for Treatment of Type II Diabetes Mellitus with Inhibitory activity against α-Amylase and α-Glucosidase. PLoS One.

[58] Chung C., Rojanasasithara T., Mutilangi W., McClements D.J. (2015). Enhanced stability of anthocyanin-based color in model beverage systems through whey protein isolate complexation. Food Res. Int..

