# Supplementary material for: Red cabbage anthocyanin-rich natural colorant: Optimization of ultrasound-assisted extraction, microencapsulation, bioaccessibility, and functional yogurt development
Source: Ultrason Sonochem. 2025 Dec 4;124:107702. doi: 10.1016/j.ultsonch.2025.107702 (PMC12732316; doi:10.1016/j.ultsonch.2025.107702)
Supplement: Supplementary Data 1 [file mmc1.docx]

**Supplementary file S3**

**Red cabbage anthocyanin-rich natural colorant: Optimization of ultrasound-assisted extraction, microencapsulation, bioaccessibility, and functional yogurt development**

Sumandeep Kaur^1#^, Amisha Rani^1#^, Reshma Thakur^1^, Abhilasha Sharma^1^, Kritika Kuksal^1^, Aman Sharma^1^, Arti Shivraj Nile^2^, Shivraj Hariram Nile^1^*

*^1^Division of Food and Nutritional Biotechnology, BRIC-National Agri-Food and Biomanufacturing Institute (Formerly National Agri-Food Biotechnology Institute), Sector-81, Mohali 140306, Punjab, India*

*^2^Department of Food Science and Technology, School of Health Sciences, Amity University,* *Sector-82A, Sahibzada Ajit Singh Nagar- 140306, Punjab, India.*

^#^These authors contributed equally

*^*^Corresponding author:*

Dr. Shivraj Hariram Nile

*E-mail address:* [*shivraj.nile@nabi.res.in*](mailto:shivraj.nile@nabi.res.in)

1. **Results**

**Table S1.** One-way ANOVA and Wilcoxon rank-sum test results comparing Non-Encapsulated (RC-NE) and Encapsulated (RC-E) red cabbage powders for phytochemical and antioxidant parameters. Values indicate normality and homogeneity test p-values, test type used, F or W statistic, significance level, post-hoc comparisons, and biological interpretation.

| **Parameter** | **Normality (Shapiro p)**  **RC-NE / RC-E** | **Homogeneity (Bartlett p)** | **Test Used** | **F / W Statistic** | **p-value** | **Significance** | **Post-hoc (Tukey HSD / Wilcoxon)** | **Interpretation** |
| --- | --- | --- | --- | --- | --- | --- | --- | --- |
| **Total Anthocyanin (CGE mg L⁻¹)** | 0.788 / 0.253 | 0.133 | ANOVA + Tukey | F = 601.6 | **1.64 × 10⁻⁵** | *** | RC-NE > RC-E (Δ = 42 mg L⁻¹, p = 2.1 × 10⁻⁵) | Highly significant reduction in anthocyanins after encapsulation |
| **Total Phenolic (mg GAE eq g⁻¹)** | 0.719 / 0.872 | 0.285 | ANOVA + Tukey | F = 100.3 | **5.6 × 10⁻⁴** | *** | RC-NE > RC-E (Δ = 224 mg g⁻¹, p = 5.6 × 10⁻⁴) | Highly significant reduction in phenolics after encapsulation |
| **Total Flavonoid (mg Rutin eq g⁻¹)** | 0.298 / 0.000 | 0.066 | Wilcoxon | W = NA | 0.0765 | ns | — | No significant difference; mild decline after encapsulation |
| **Total Flavonols (mg Rutin eq g⁻¹)** | 0.726 / 0.811 | 0.145 | ANOVA + Tukey | F = 6.94 | 0.0579 | • | RC-NE > RC-E (Δ = 2.3 mg g⁻¹, p = 0.058) | Marginally lower in RC-E; borderline significance |
| **Antioxidant (DPPH %)** | 0.942 / 0.165 | 0.141 | ANOVA + Tukey | F = 67.8 | **0.00119** | ** | RC-NE > RC-E (Δ = 17.1 %, p = 0.0012) | Significant decrease in DPPH activity after encapsulation |
| **FRAP (µM Fe²⁺ eq L⁻¹)** | 0.490 / 0.537 | 0.237 | ANOVA + Tukey | F = 0.027 | 0.877 | ns | RC-NE ≈ RC-E (p = 0.877) | No significant difference; FRAP unaffected by encapsulation |
| **ABTS (% Inhibition)** | 0.843 / 0.878 | 0.753 | ANOVA + Tukey | F = 5144 | **2.26 × 10⁻⁷** | *** | RC-NE > RC-E (Δ = 23.7 %, p = 2.3 × 10⁻⁷) | Extremely significant reduction in ABTS scavenging capacity |

**Significance codes:** *** p < 0.001; ** p < 0.01; * p < 0.05; • p < 0.10; ns = not significant.

**Table S2.** Sensory evaluation of control and anthocyanin-fortified yoghurt samples, showing mean ± standard deviation (n = 20) for color, taste, aroma, texture, and overall acceptability. Statistical analysis was performed using one-way ANOVA followed by Tukey’s HSD test; values with *p* < 0.05 were considered significant.

| **Parameter** | **F value** | **p value** | **Significance** | **Interpretation** |
| --- | --- | --- | --- | --- |
| **Color** | 0.504 | 0.4822 | ns | No significant difference in color perception between control and anthocyanin yogurt. |
| **Taste** | 15.414 | 0.0004 | *** | Anthocyanin yogurt was significantly preferred in taste over control. |
| **Aroma** | 13.420 | 0.0008 | *** | Anthocyanin yogurt exhibited a significantly enhanced aroma compared to control. |
| **Texture** | 0.000 | 1.0000 | ns | No significant difference in texture between the two samples. |
| **Overall Acceptability** | 7.273 | 0.0104 | * | Anthocyanin yogurt showed significantly higher overall acceptability. |

Significance codes: ***p* < 0.001; *p < 0.01; p < 0.05; ns = not significant. Values derived from one-way ANOVA (n = 20 per group) followed by Tukey’s post-hoc test.*


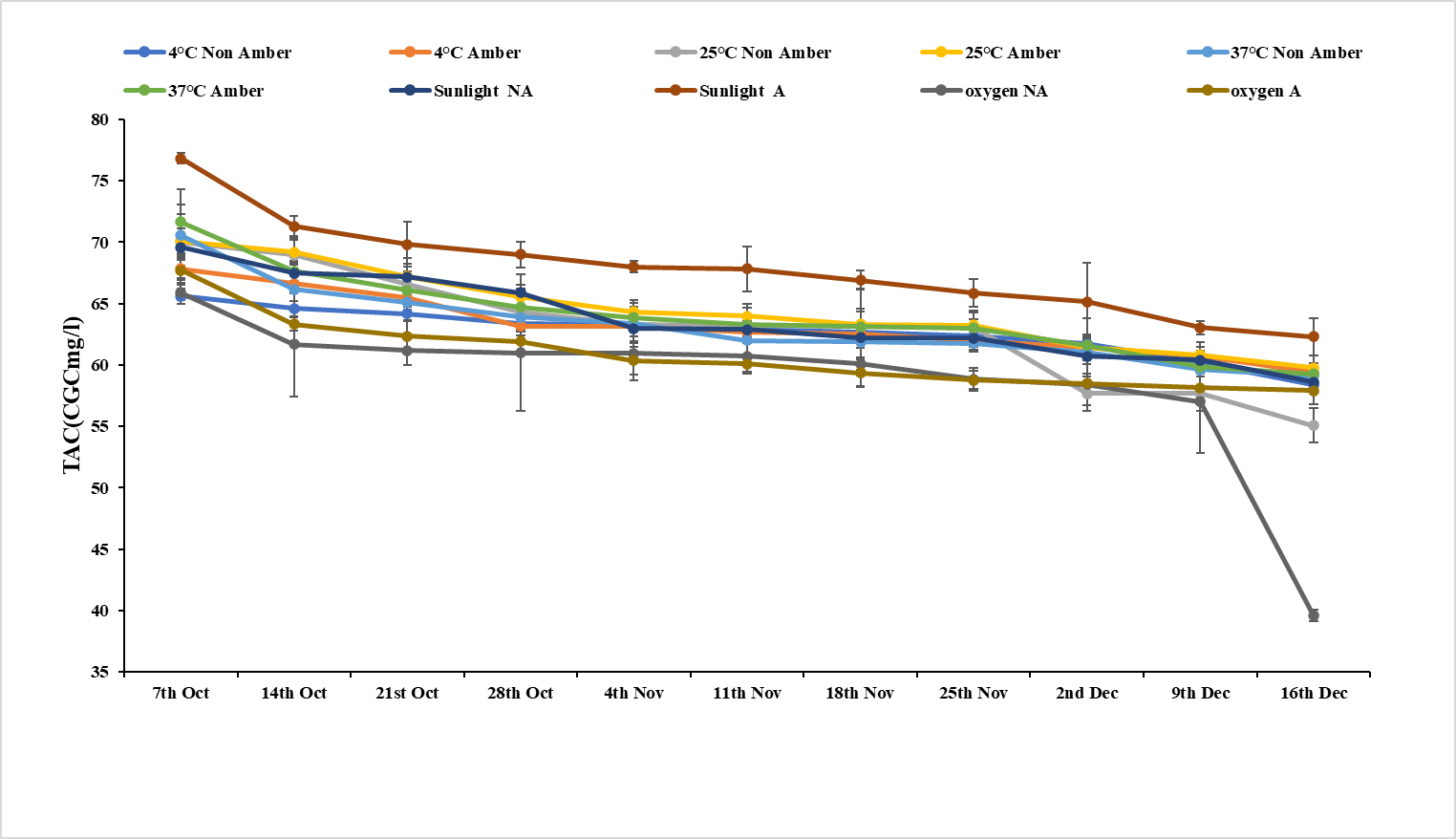


**Figure S1.** Shelf-life stability of encapsulated red cabbage anthocyanin powder under different storage conditions over eight weeks. Total anthocyanin content (TAC, expressed as CGE mg·L⁻¹) was monitored at 4 °C, 25 °C, and 37 °C under amber and non-amber light exposure, as well as under sunlight and oxygen environments. Error bars represent standard deviation (n = 3). Encapsulation effectively preserved anthocyanin stability, particularly under amber and low-temperature conditions.
