# Supplementary material for: Red cabbage anthocyanin-rich natural colorant: Optimization of ultrasound-assisted extraction, microencapsulation, bioaccessibility, and functional yogurt development
Source: Ultrason Sonochem. 2025 Dec 4;124:107702. doi: 10.1016/j.ultsonch.2025.107702 (PMC12732316; doi:10.1016/j.ultsonch.2025.107702)

Openlynx Report SAIF,PANJAB UNIVERSITY CHANDIGARH

Sample: 64 | Vial:1:F,2 | ID:

File:AMISHA_S_1_ | Date:29-Oct-2025 | Time:20:32:07

Description:

Printed: Thu Oct 30 16:32:16 2025

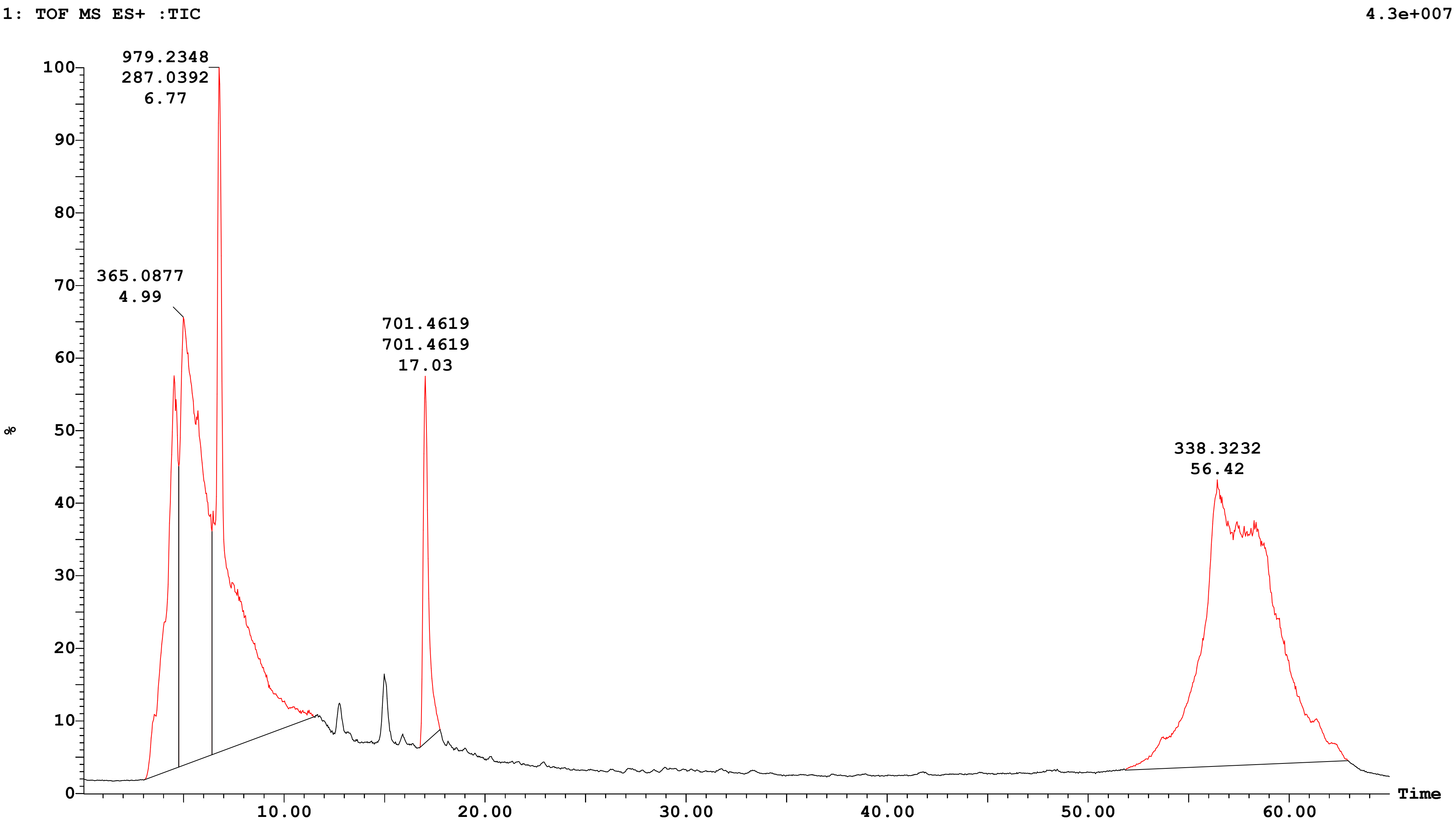

Peak Number	Compound	Time	AreaAbs	Area %Total	Width	Height	Mass Found
1		4.53	2e+007	10.06	2	2e+007	
4		4.99	3e+007	21.28	2	3e+007	
7		6.77	3e+007	20.62	5	4e+007	
10		17.03	7e+006	4.27	1	2e+007	
12		56.42	7e+007	43.78	11	2e+007	

Openlynx Report SAIF,PANJAB UNIVERSITY CHANDIGARH

Sample: 64 | Vial:1:F,2 | ID:
File:AMISHA_S_1_ | Date:29-Oct-2025 | Time:20:32:07
Description:

Printed: Thu Oct 30 16:32:16 2025

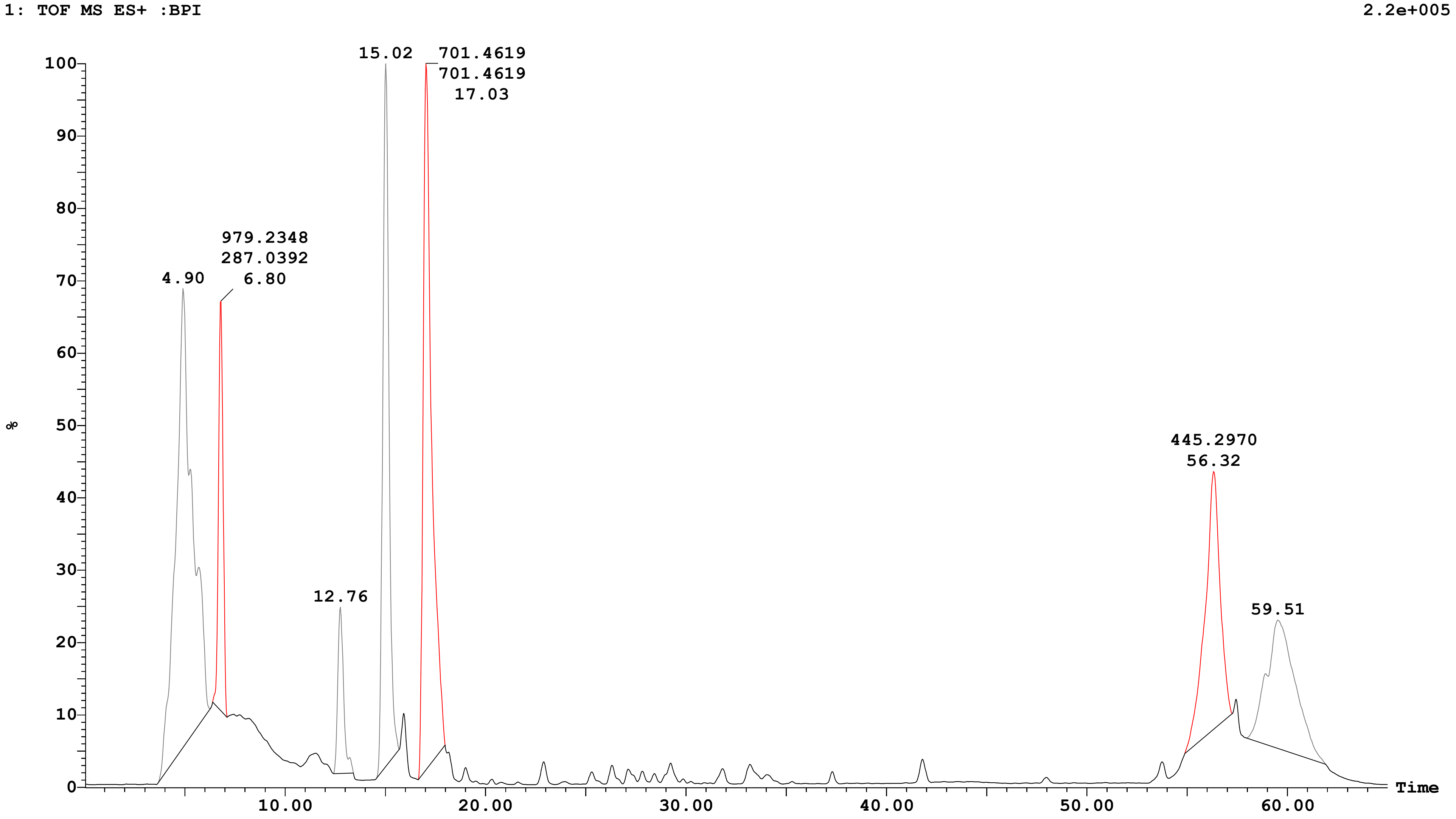

Sample: 64 | Vial:1:F,2 | ID:
File:AMISHA_S_1_ | Date:29-Oct-2025 | Time:20:32:07
Description:

Printed: Thu Oct 30 16:32:16 2025

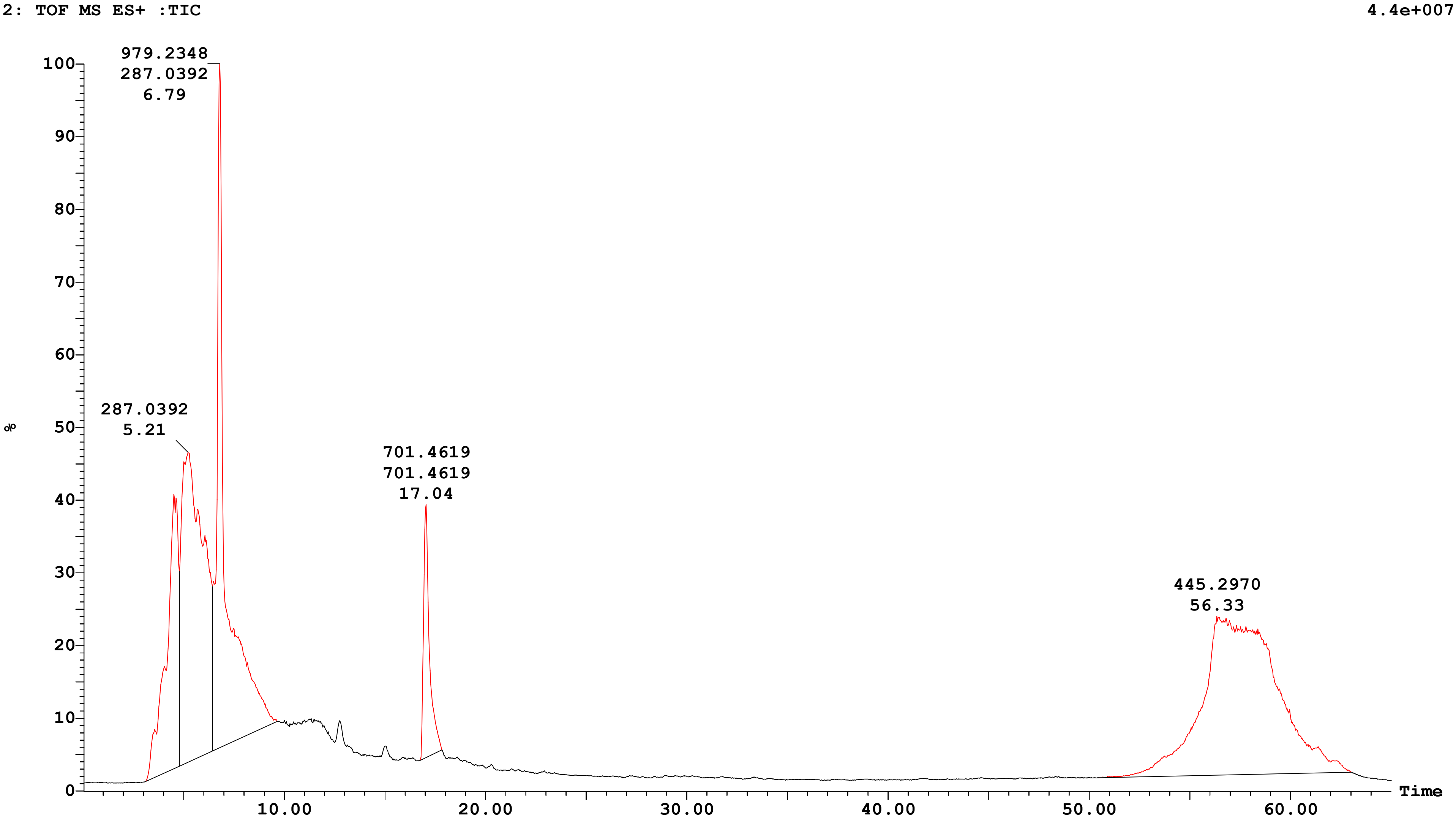

Peak Number	Compound	Time	AreaAbs	Area %Total	Width	Height	Mass Found
1		4.51	1e+007	10.97	2	2e+007	
5		5.21	2e+007	23.12	2	2e+007	
7		6.79	2e+007	21.07	3	4e+007	
10		17.04	5e+006	4.97	1	2e+007	
11		56.33	4e+007	39.87	13	1e+007	

Openlynx Report SAIF,PANJAB UNIVERSITY CHANDIGARH

Sample: 64 | Vial:1:F,2 | ID:
File:AMISHA_S_1_ | Date:29-Oct-2025 | Time:20:32:07
Description:

Printed: Thu Oct 30 16:32:16 2025

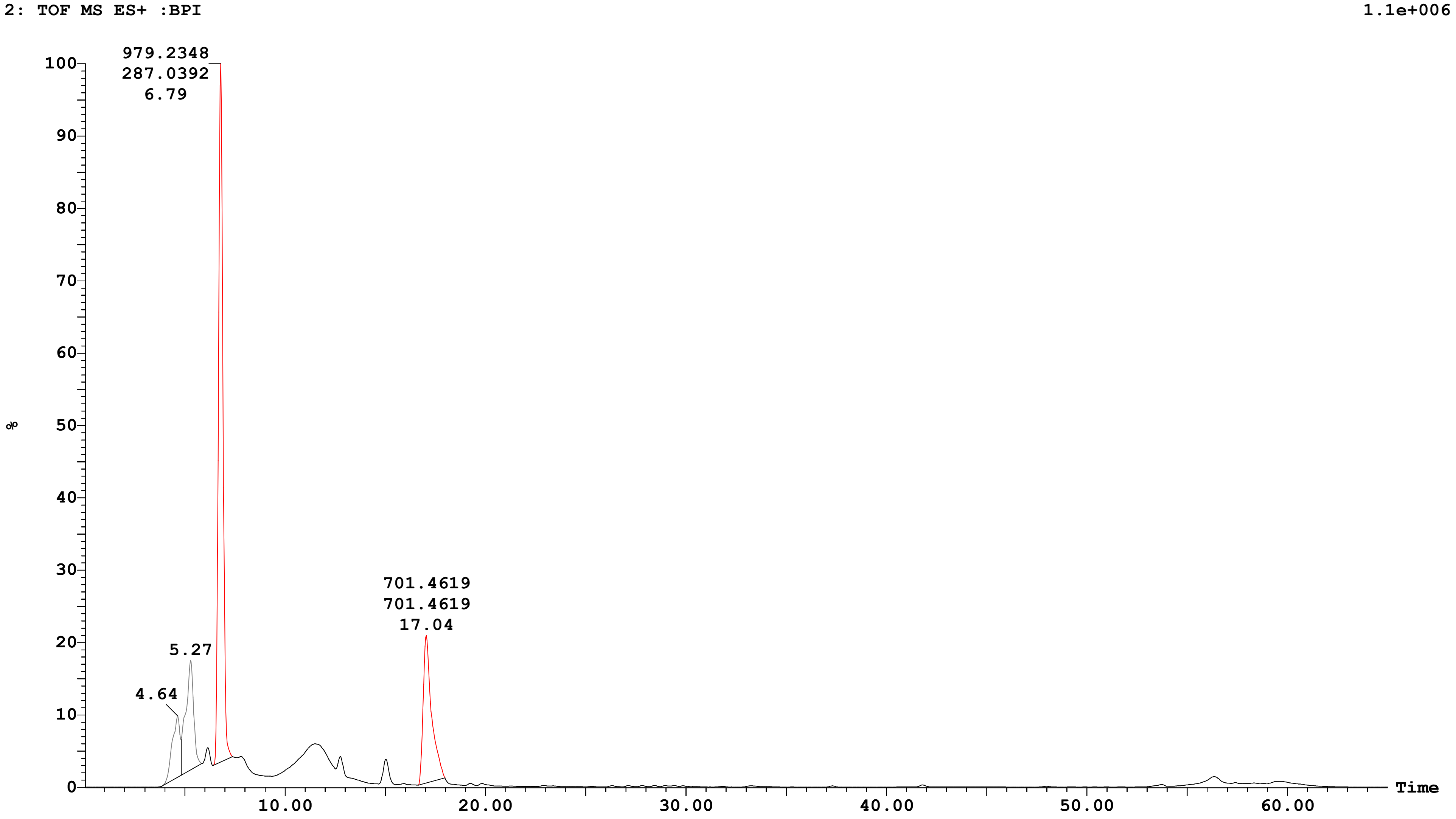

Sample: 64 Vial:1:F,2 ID:
File:AMISHA_S_1_ Date:29-Oct-2025 Time:20:32:07
Description:

Printed: Thu Oct 30 16:32:16 2025

Peak ID	Time
1	4.53

(Time: 4.53)

1:TOF MS ES+
8.6e+005

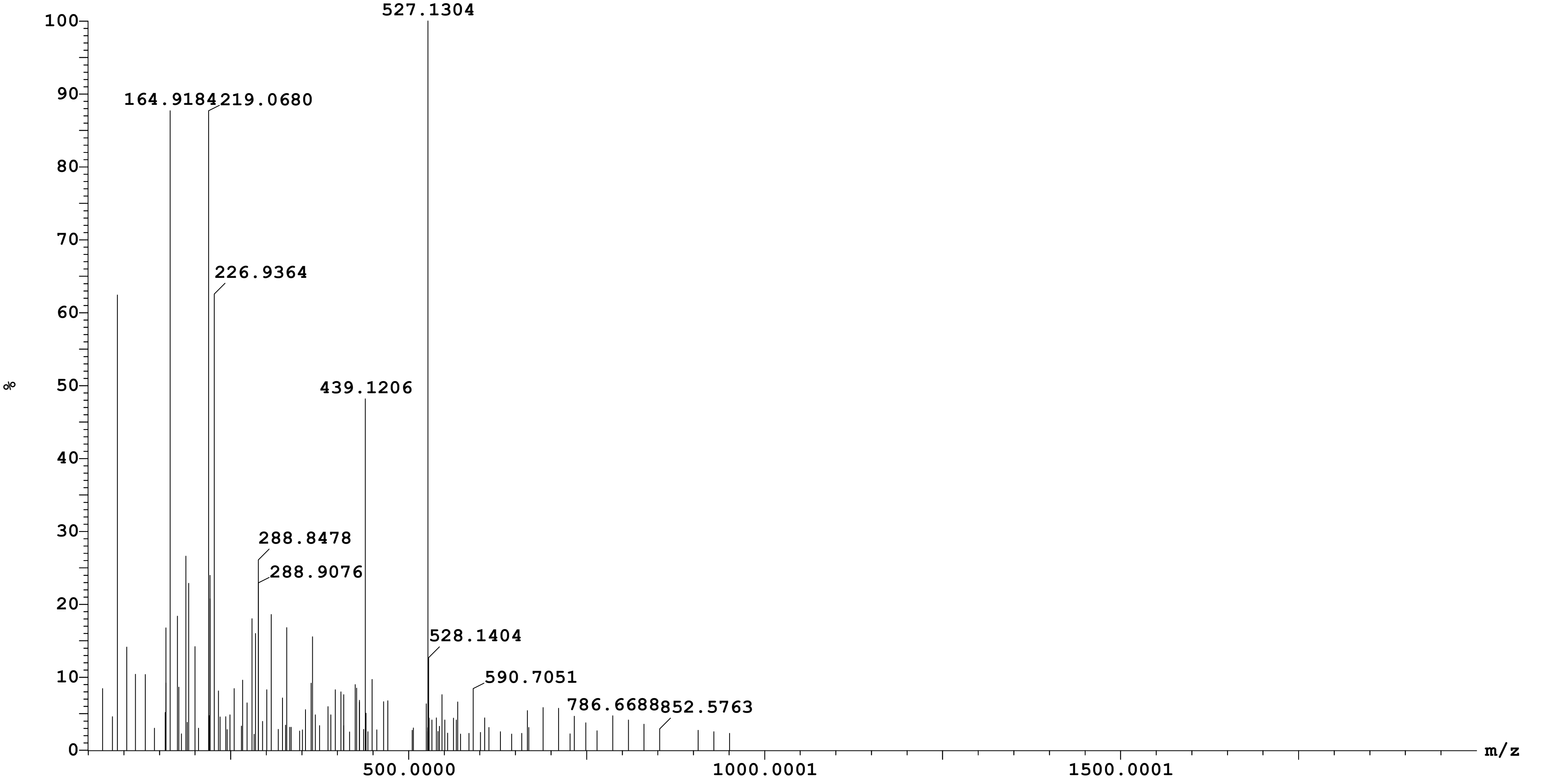

Sample: 64 Vial:1:F,2 ID:
File:AMISHA_S_1_ Date:29-Oct-2025 Time:20:32:07
Description:

Printed: Thu Oct 30 16:32:16 2025

Peak ID	Time
4	4.99

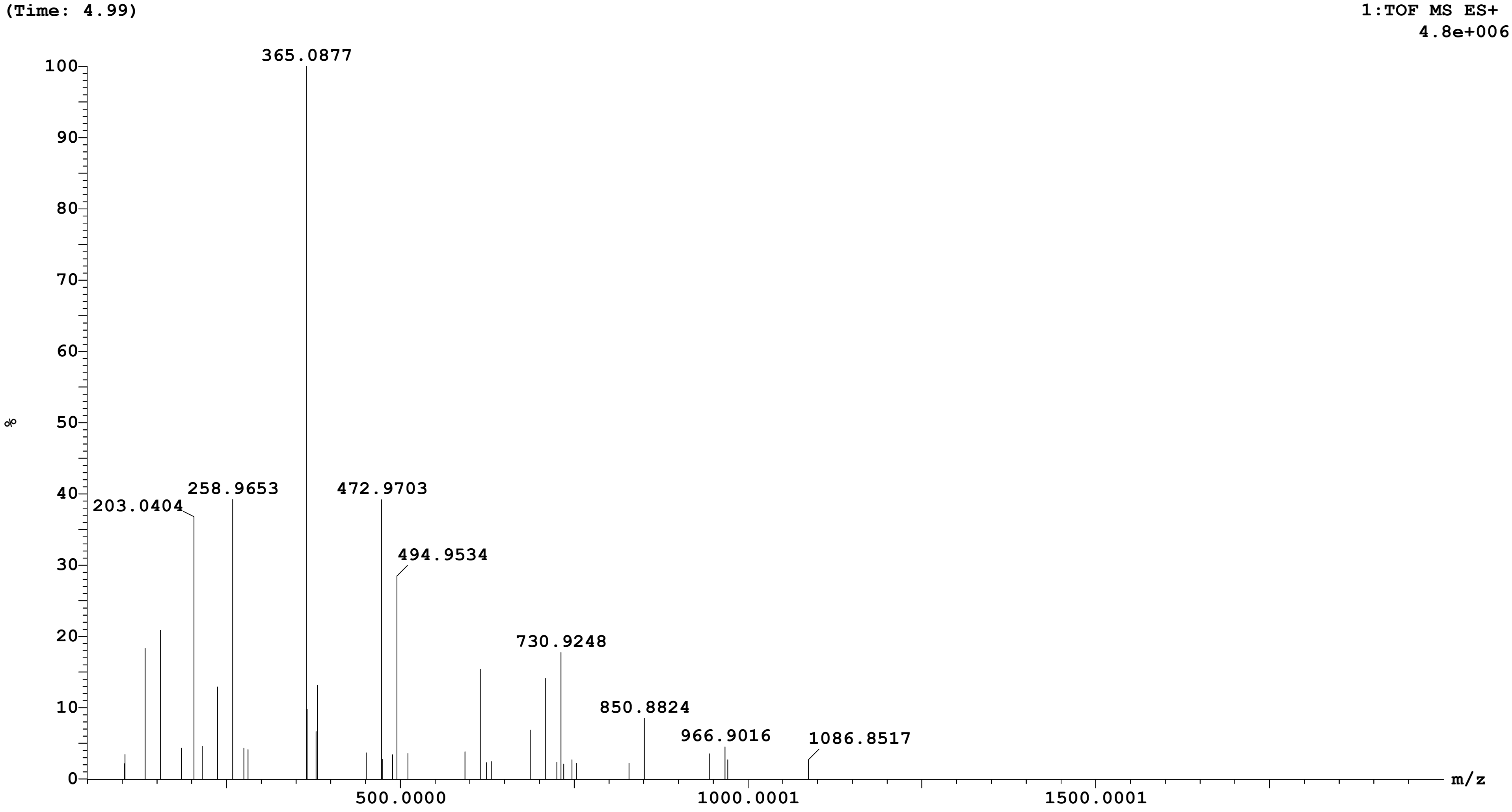

Sample: 64 Vial:1:F,2 ID:
File:AMISHA_S_1_ Date:29-Oct-2025 Time:20:32:07
Description:

Printed: Thu Oct 30 16:32:16 2025

Peak ID	Time
7	6.77

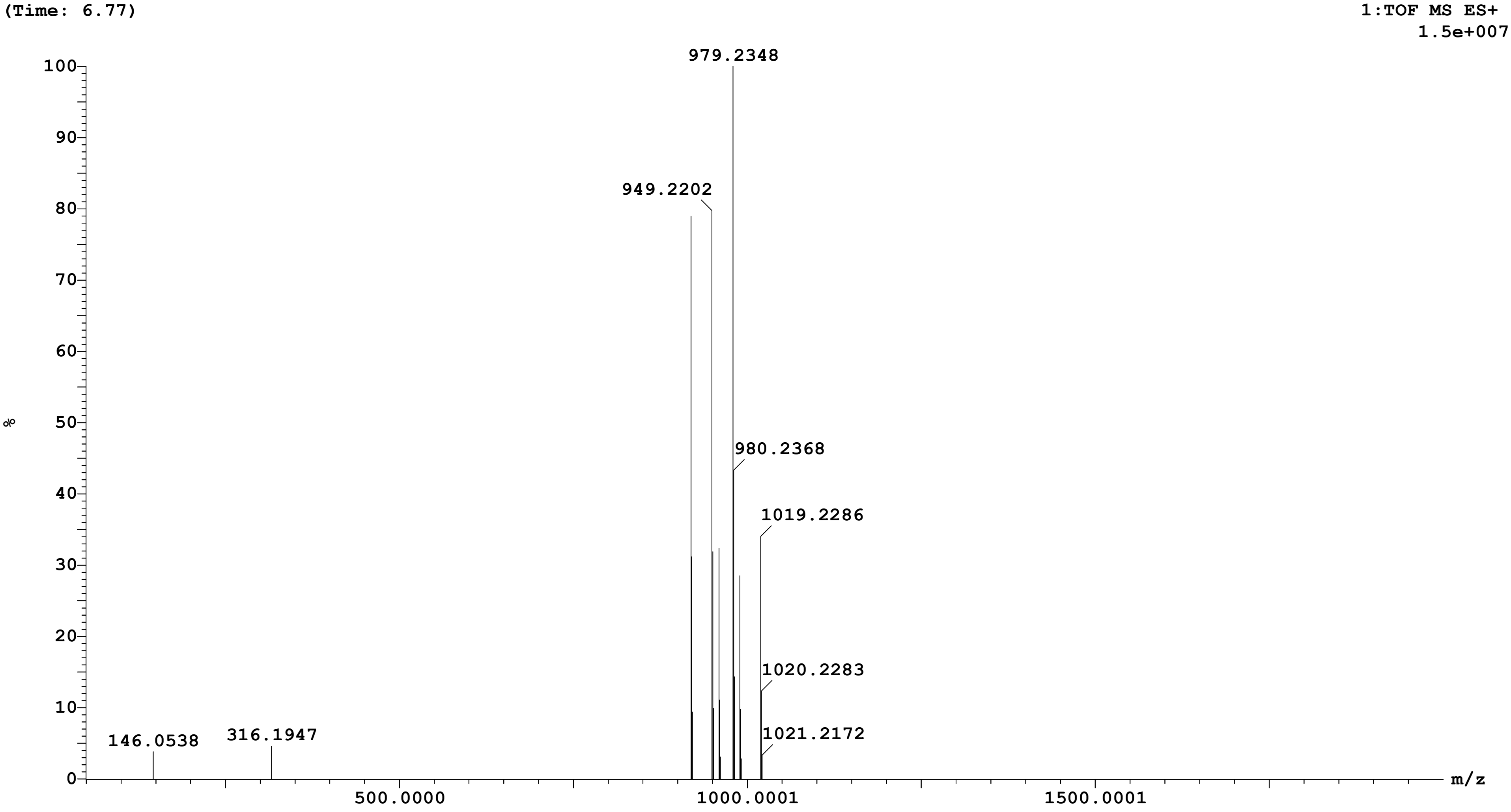

Sample: 64 Vial:1:F,2 ID:
File:AMISHA_S_1_ Date:29-Oct-2025 Time:20:32:07
Description:

Printed: Thu Oct 30 16:32:16 2025

**Peak ID**	**Time**
10	17.03

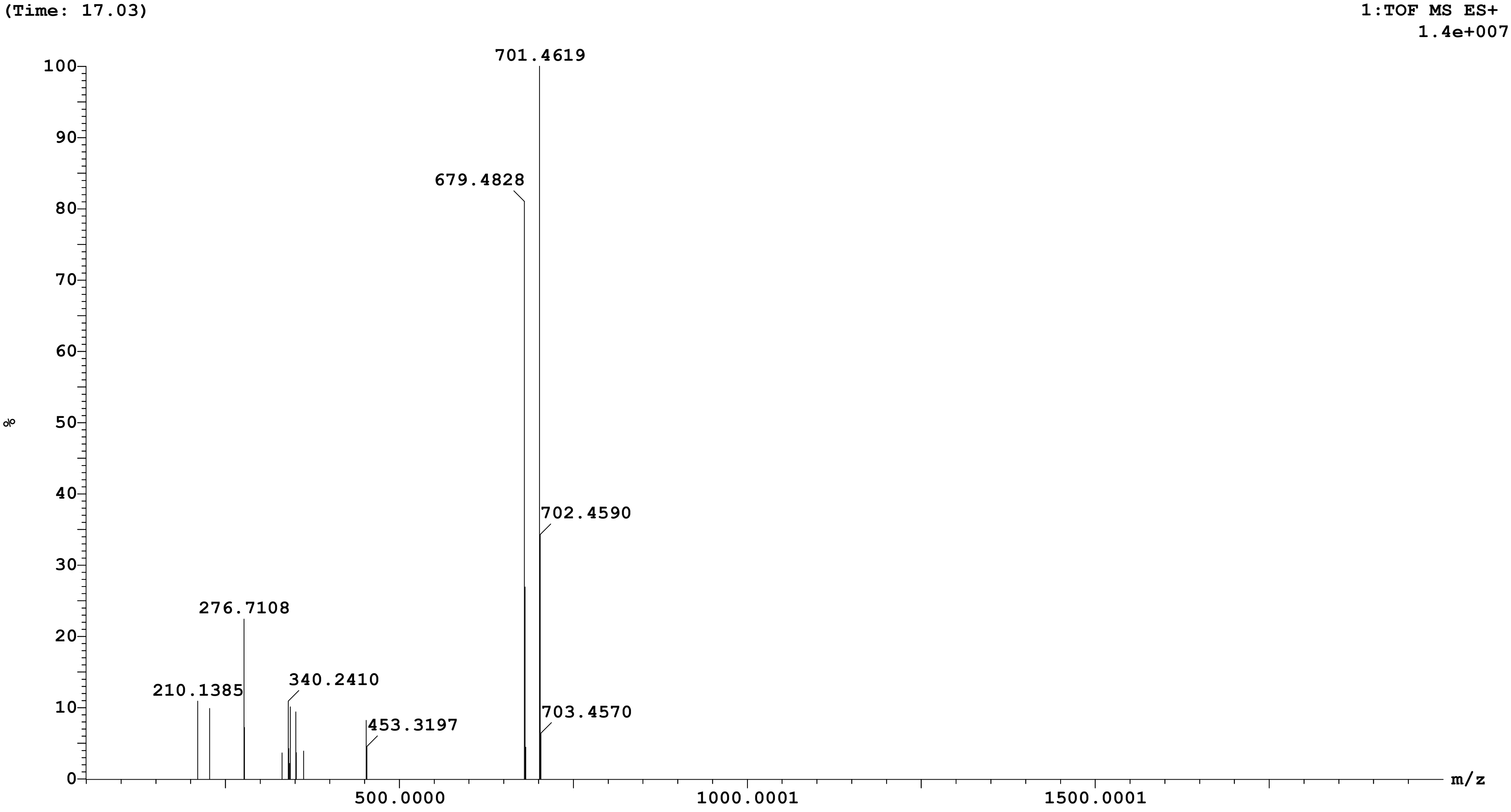

Sample: 64 Vial:1:F,2 ID:
File:AMISHA_S_1_ Date:29-Oct-2025 Time:20:32:07
Description:

Printed: Thu Oct 30 16:32:16 2025

**Peak ID**	**Time**
12	56.42

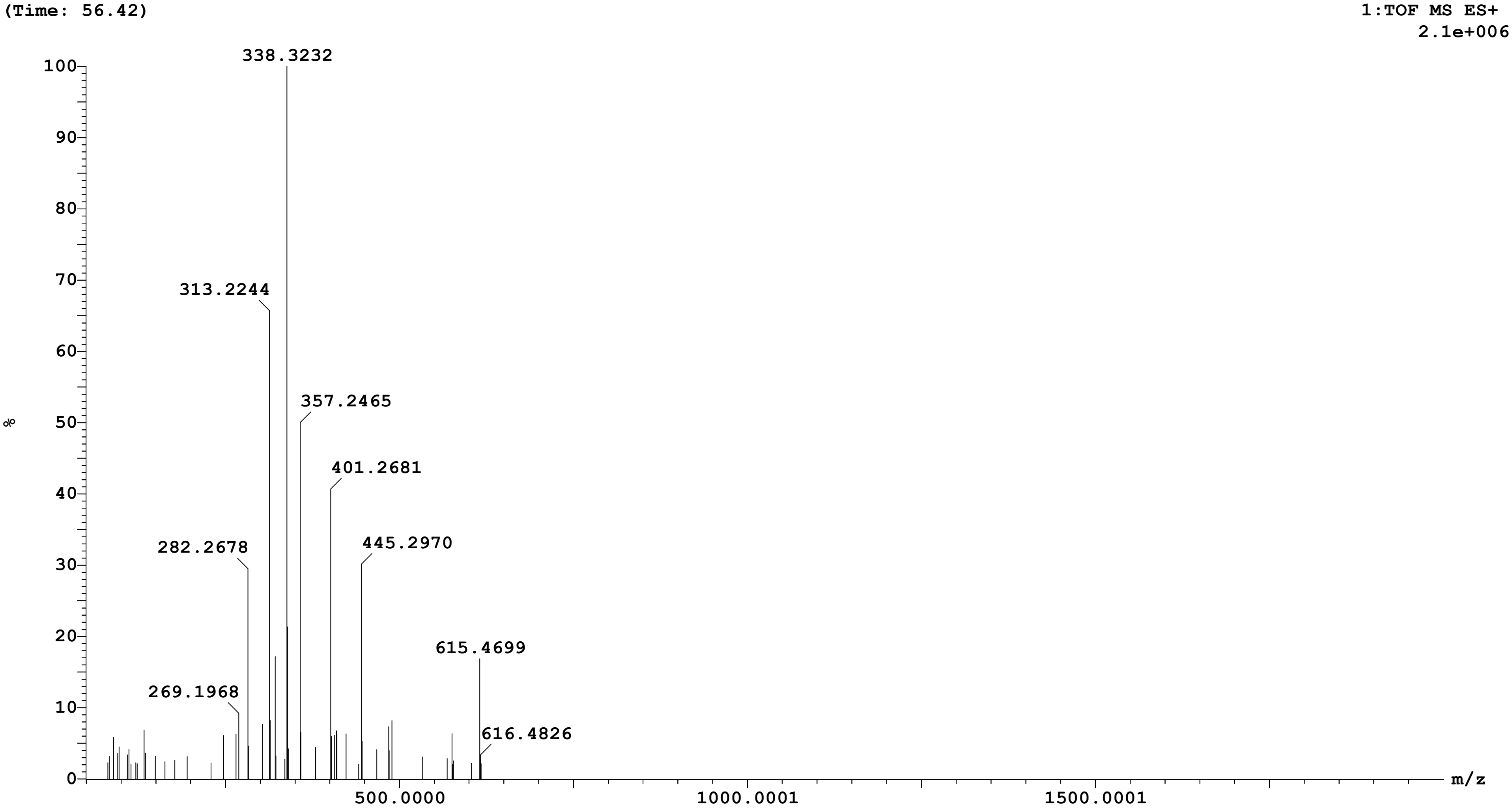

Sample: 64 Vial:1:F,2 ID:
File:AMISHA_S_1_ Date:29-Oct-2025 Time:20:32:07
Description:

Printed: Thu Oct 30 16:32:16 2025

Peak ID	Time
1	4.53

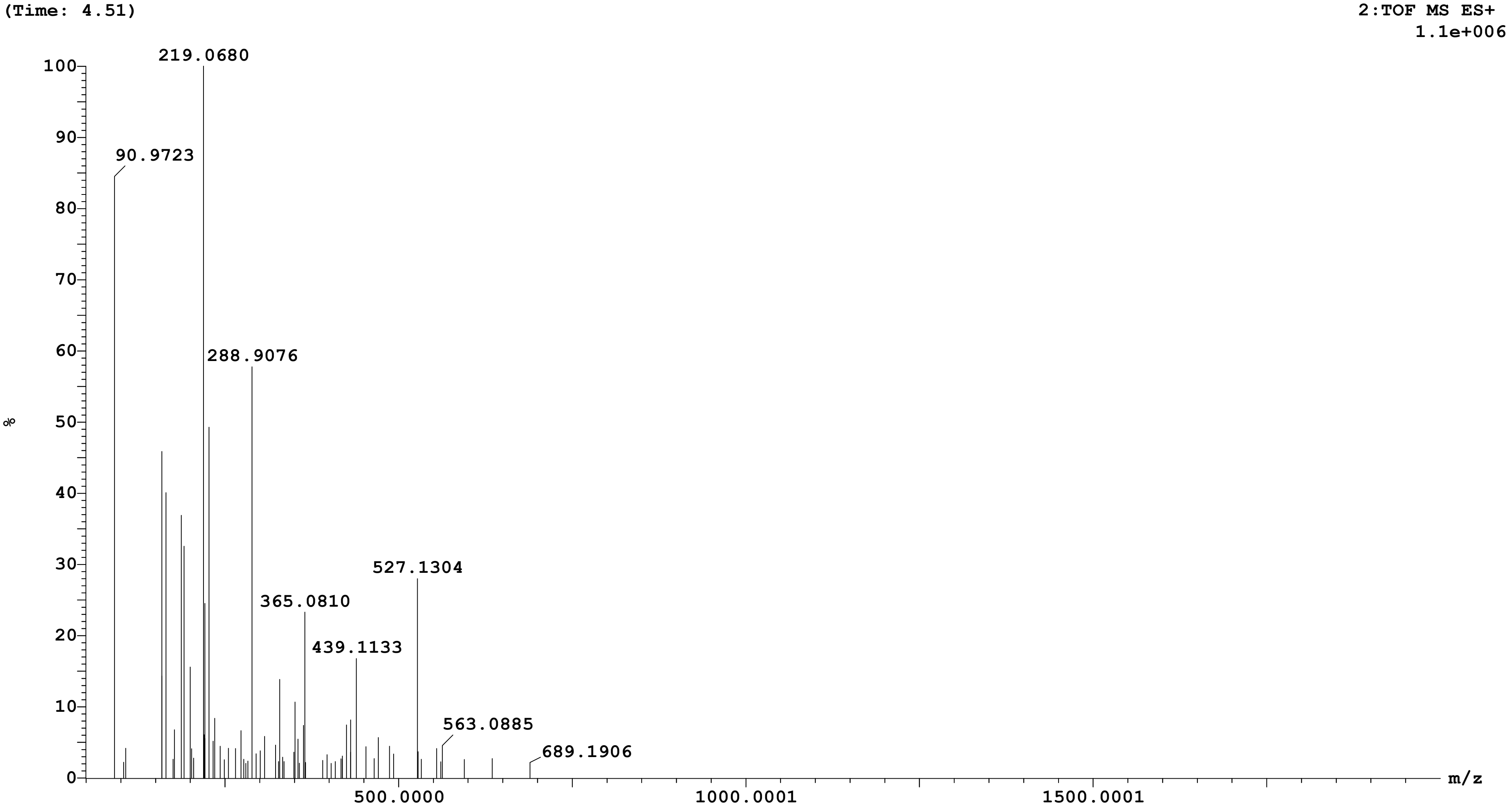

Sample: 64 Vial:1:F,2 ID:
File:AMISHA_S_1_ Date:29-Oct-2025 Time:20:32:07
Description:

Printed: Thu Oct 30 16:32:16 2025

Peak ID	Time
5	5.21

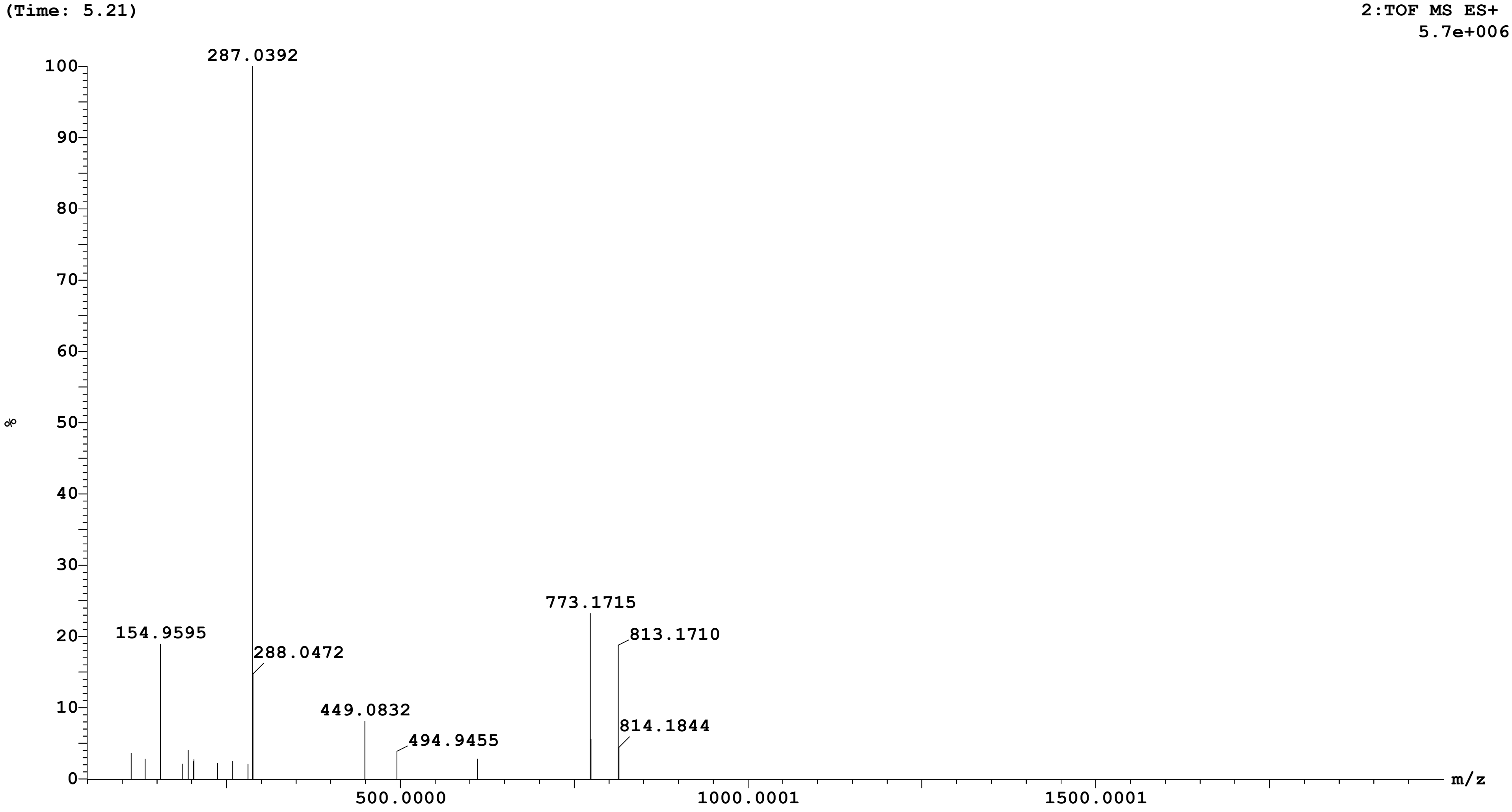

Sample: 64 Vial:1:F,2 ID:
File:AMISHA_S_1_ Date:29-Oct-2025 Time:20:32:07
Description:

Printed: Thu Oct 30 16:32:16 2025

Peak ID	Time
7	6.77

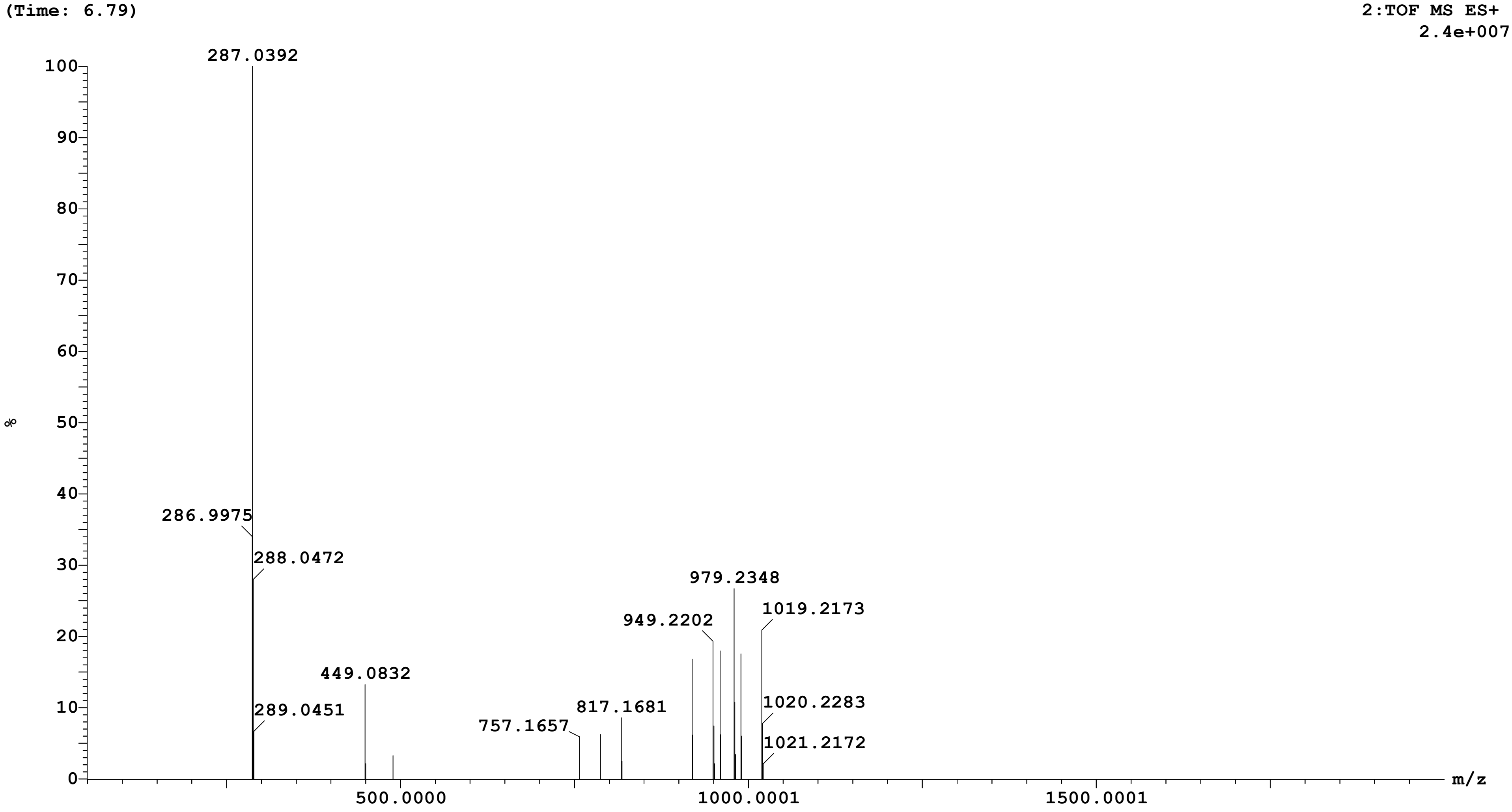

Sample: 64 Vial:1:F,2 ID:
File:AMISHA_S_1_ Date:29-Oct-2025 Time:20:32:07
Description:

Printed: Thu Oct 30 16:32:16 2025

**Peak ID**	**Time**
10	17.03

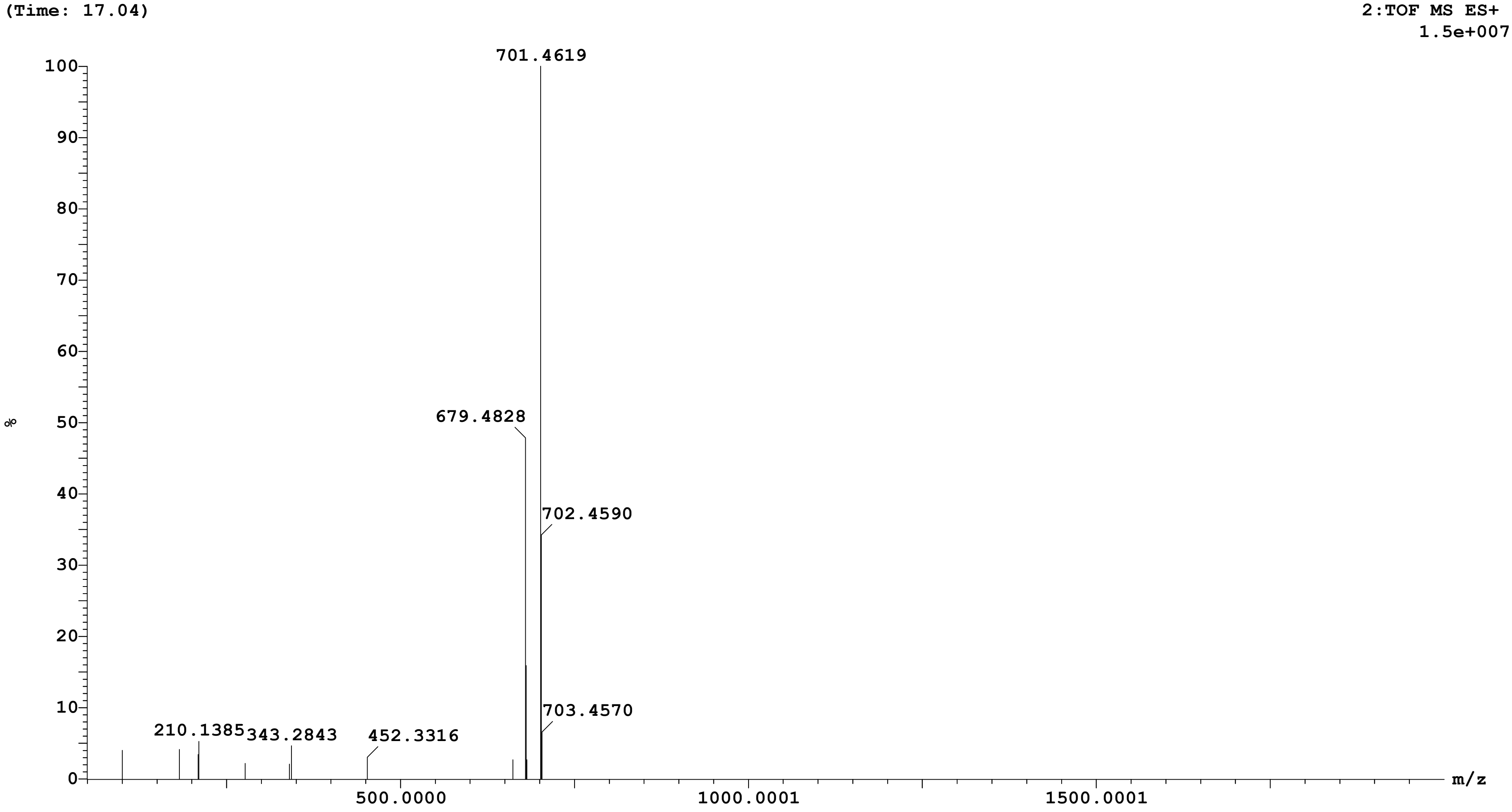

Sample: 64 | Vial:1:F,2 | ID:
File:AMISHA_S_1_ | Date:29-Oct-2025 | Time:20:32:07
Description:

Printed: Thu Oct 30 16:32:16 2025

Peak ID	Time
11	56.32

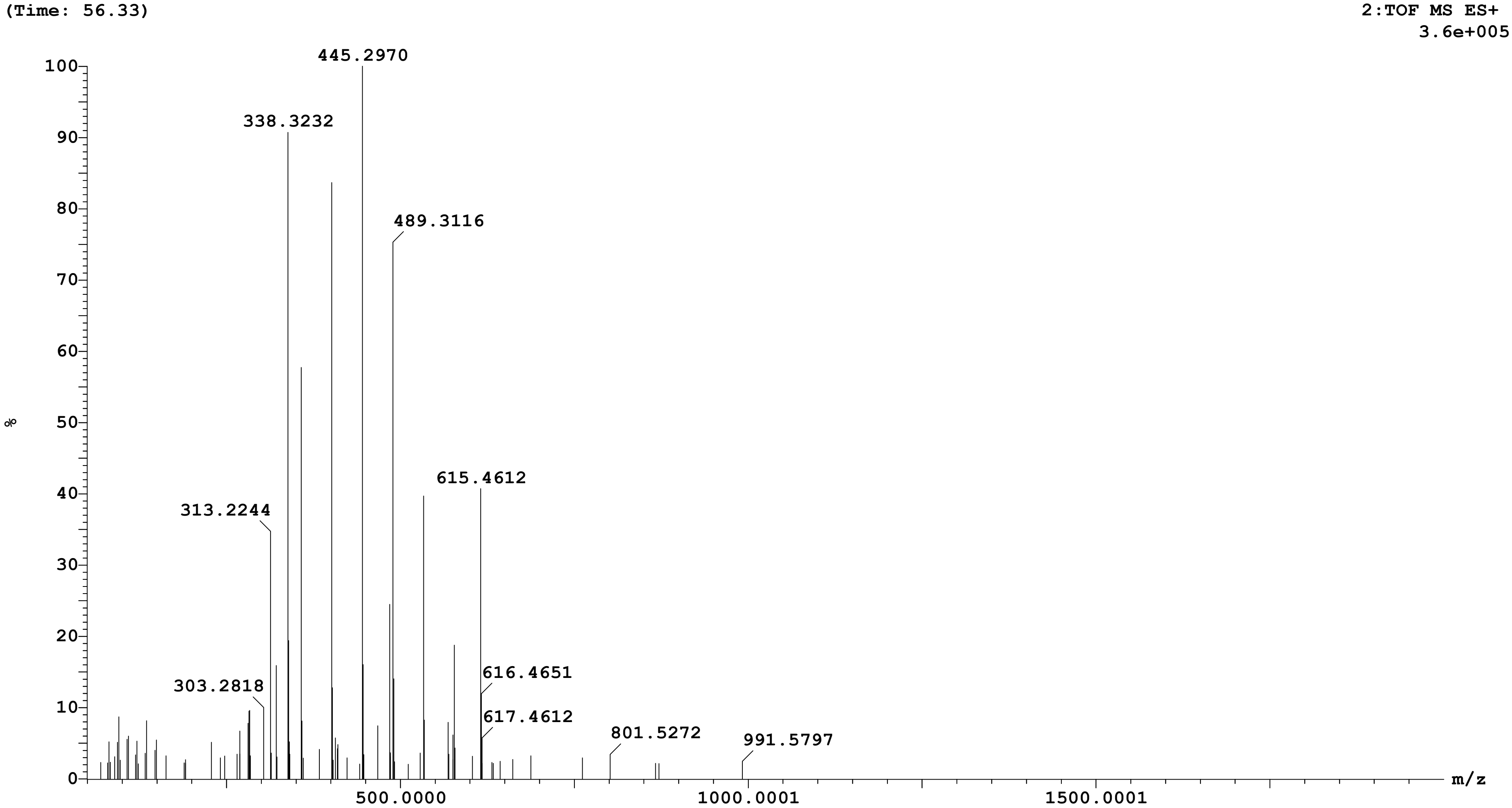

Openlynx Report SAIF,PANJAB UNIVERSITY CHANDIGARH

Sample: 66 | Vial:1:F,3 | ID:
File:AMISHA_S_2 | Date:29-Oct-2025 | Time:18:19:36
Description:

Printed: Thu Oct 30 16:32:41 2025

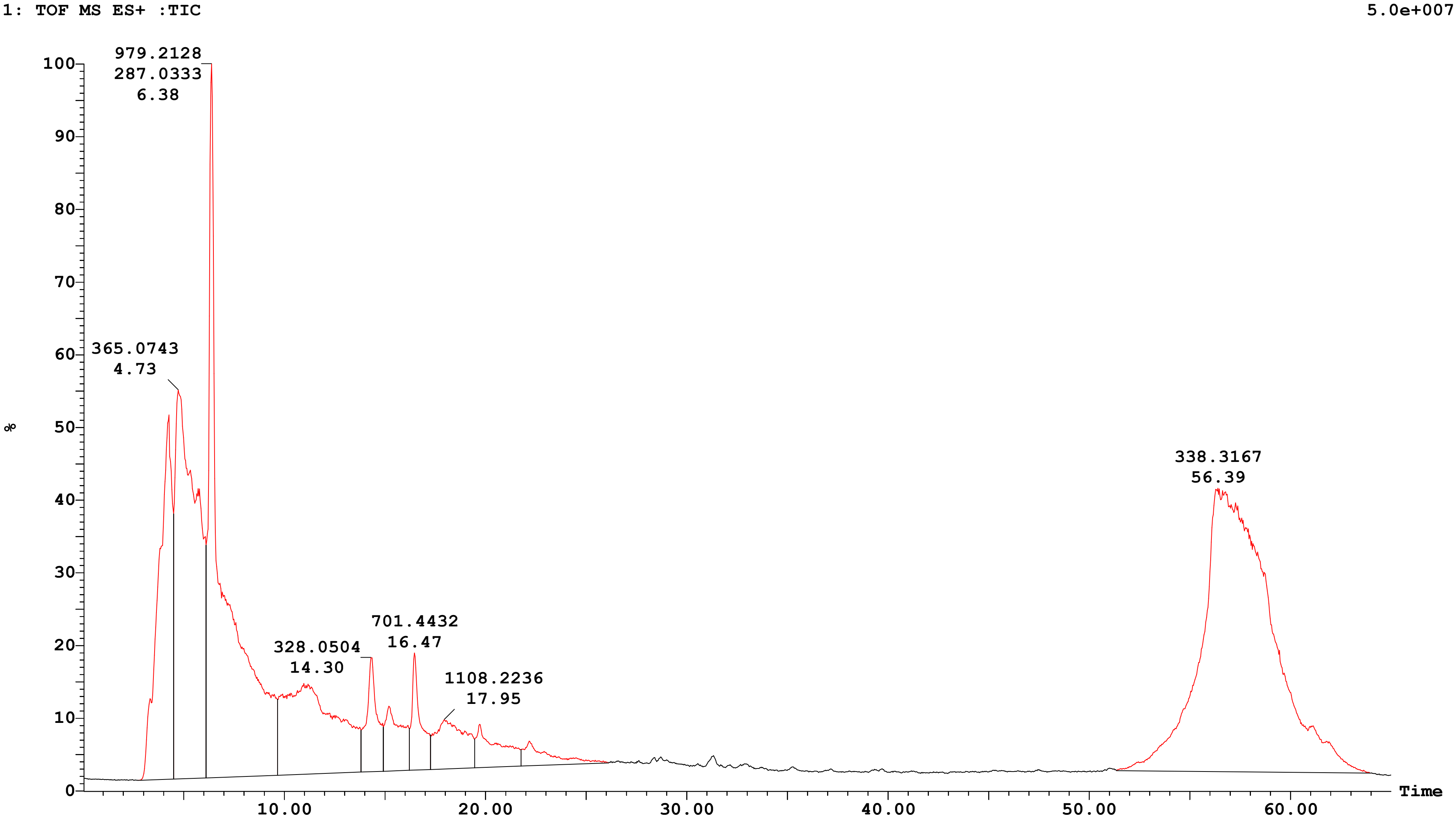

Peak Number	Compound	Time	AreaAbs	Area %Total	Width	Height	Mass Found
3		4.27	2e+007	8.88	2	2e+007	
5		4.73	3e+007	15.54	2	3e+007	
10		6.38	4e+007	18.88	4	5e+007	
11		10.97	2e+007	8.80	4	6e+006	
13		14.30	5e+006	2.24	1	8e+006	

Sample: 66 Vial:1:F,3 ID:
File:AMISHA_S_2 Date:29-Oct-2025 Time:18:19:36
Description:

Printed: Thu Oct 30 16:32:41 2025

14	15.19	4e+006	1.98	1	4e+006
15	16.47	4e+006	1.87	1	8e+006
16	17.95	6e+006	2.69	2	3e+006
17	19.70	4e+006	1.74	2	3e+006
18	22.16	2e+006	1.12	4	2e+006
19	56.39	8e+007	36.26	13	2e+007

Sample: 66 Vial:1:F,3 ID:
File:AMISHA_S_2 Date:29-Oct-2025 Time:18:19:36
Description:

Printed: Thu Oct 30 16:32:41 2025

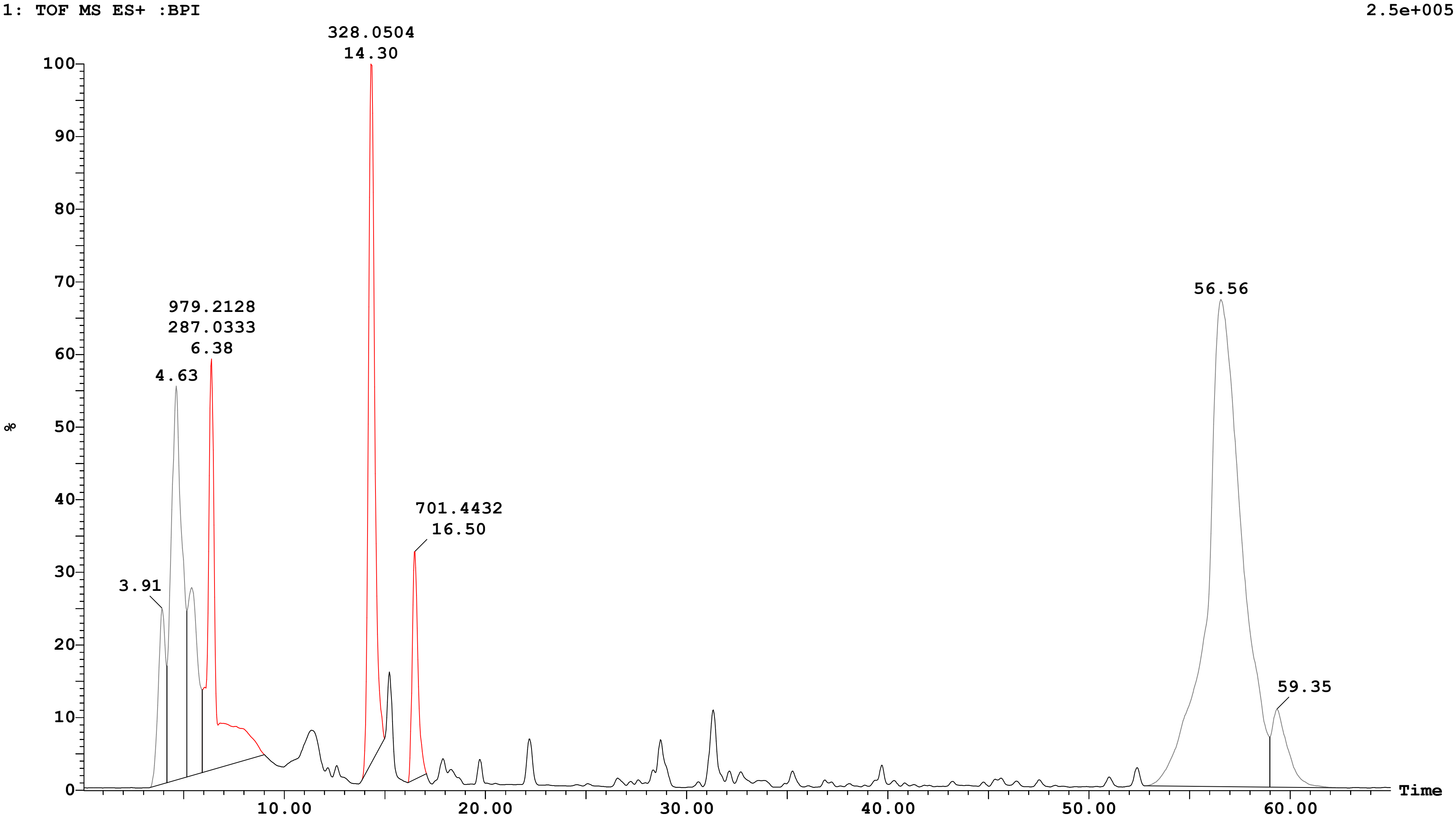

Sample: 66 Vial:1:F,3 ID:
File:AMISHA_S_2 Date:29-Oct-2025 Time:18:19:36
Description:

Printed: Thu Oct 30 16:32:41 2025

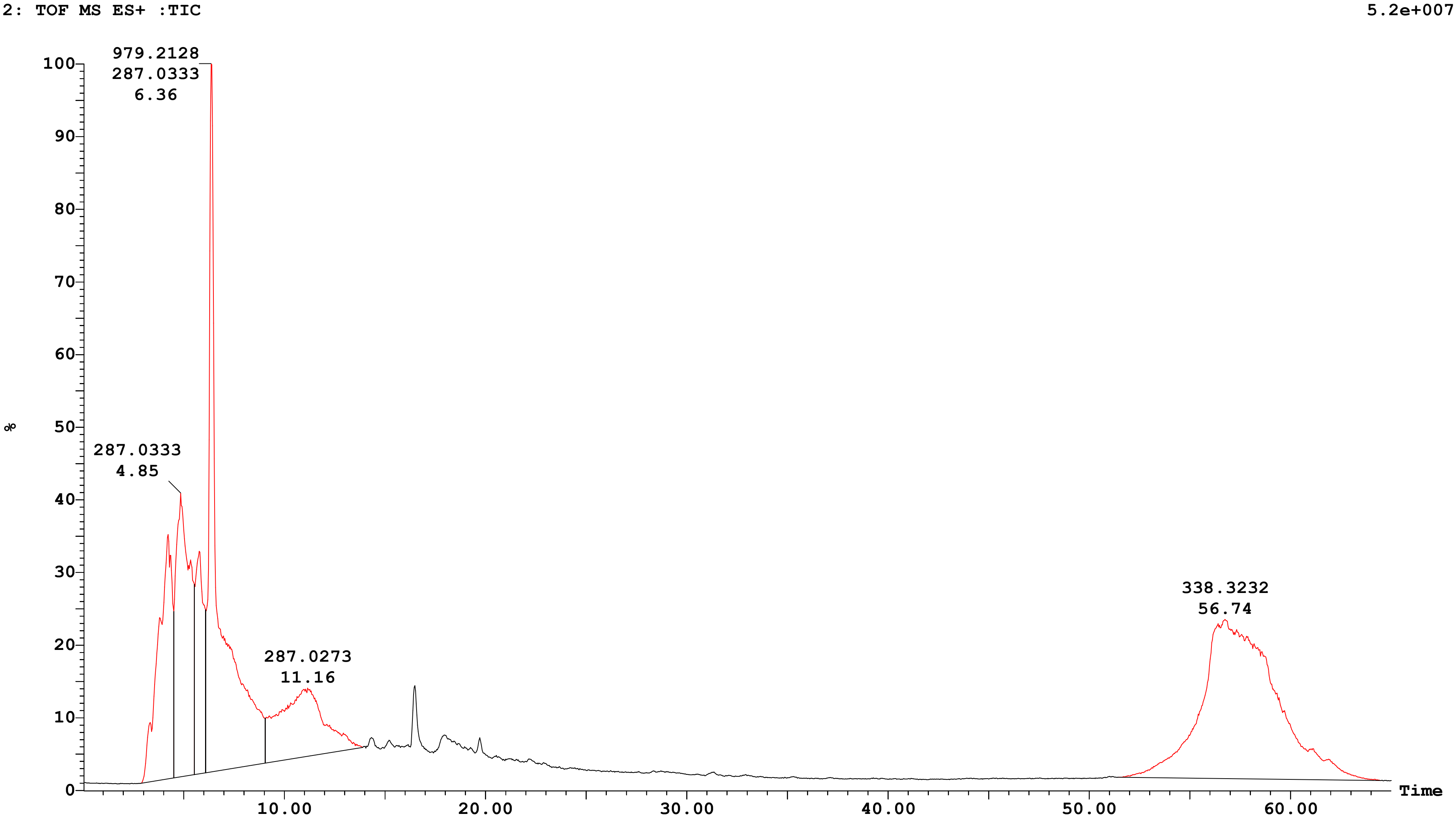

Peak Number	Compound	Time	AreaAbs	Area %Total	Width	Height	Mass Found
2		4.22	1e+007	10.75	2	2e+007	
6		4.85	2e+007	12.65	1	2e+007	
9		5.77	8e+006	5.91	1	2e+007	
10		6.36	3e+007	23.22	3	5e+007	
12		11.16	1e+007	9.96	5	5e+006	

Sample: 66 Vial:1:F,3 ID:
File:AMISHA_S_2 Date:29-Oct-2025 Time:18:19:36
Description:

Printed: Thu Oct 30 16:32:41 2025

21	56.74	5e+007	37.51	13	1e+007

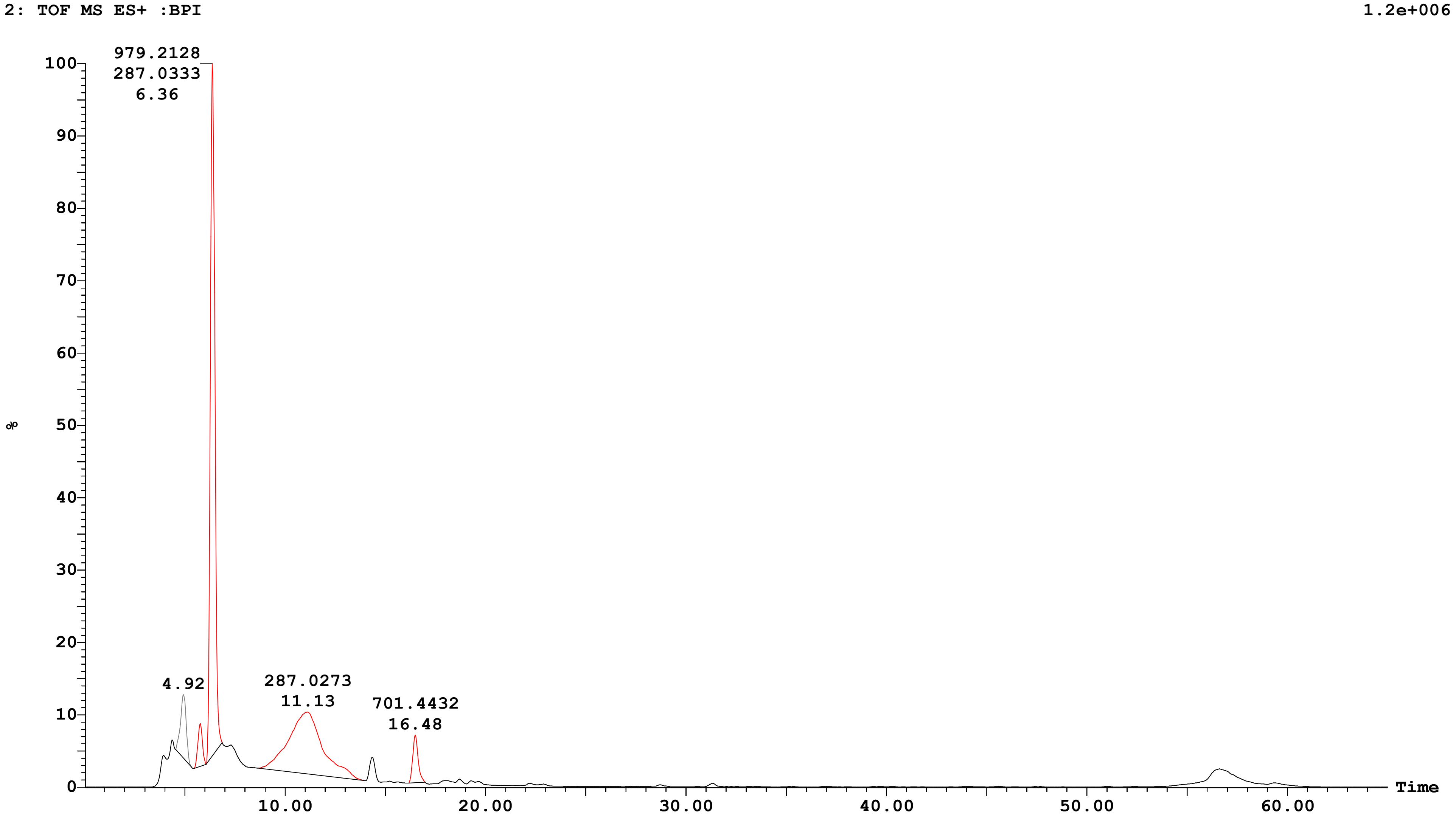

Sample: 66 Vial:1:F,3 ID:
File:AMISHA_S_2 Date:29-Oct-2025 Time:18:19:36
Description:

Printed: Thu Oct 30 16:32:41 2025

**Peak ID**	**Time**
3	4.27

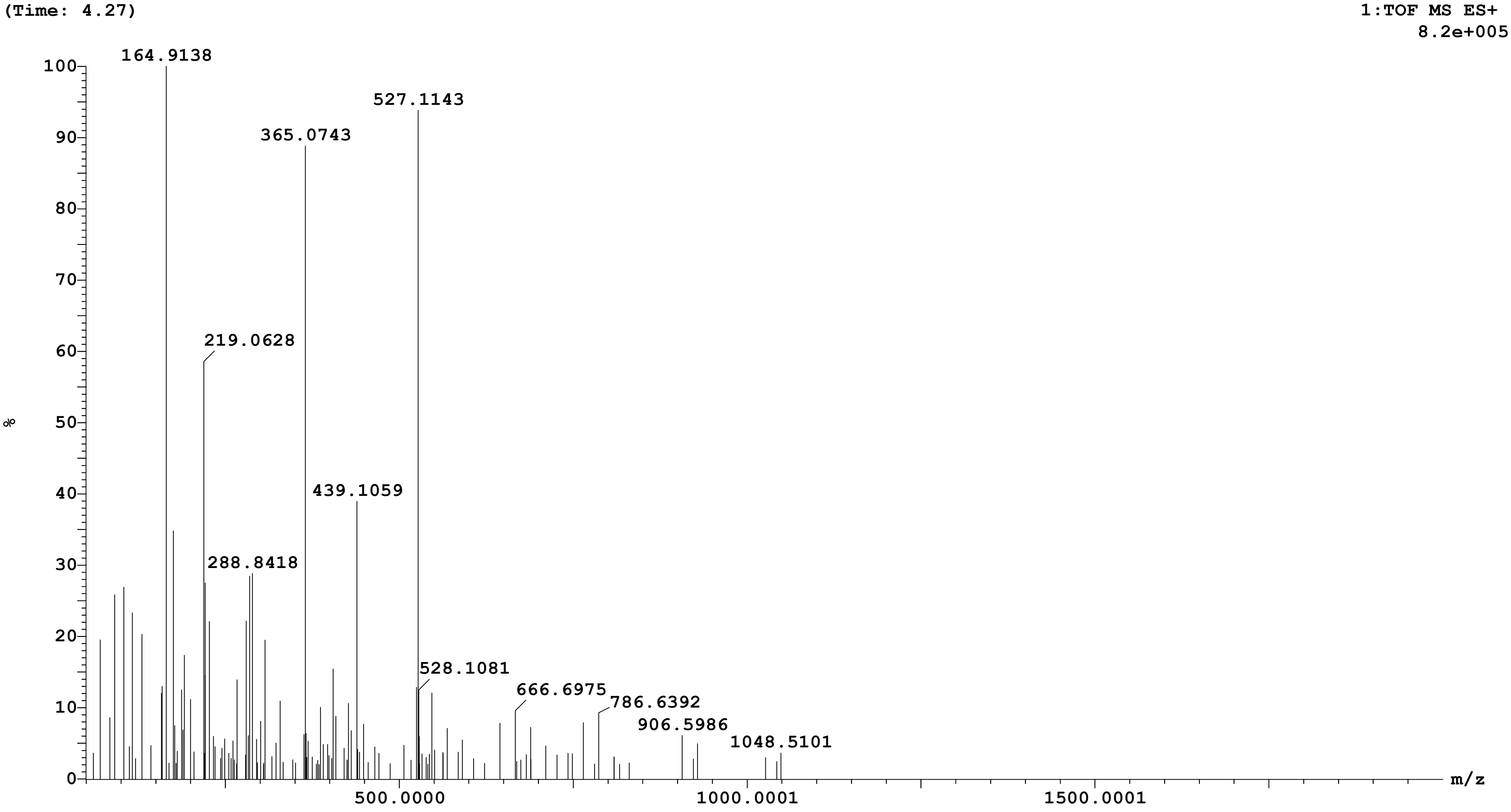

Sample: 66 Vial:1:F,3 ID:
File:AMISHA_S_2 Date:29-Oct-2025 Time:18:19:36
Description:

Printed: Thu Oct 30 16:32:41 2025

Peak ID	Time
5	4.73

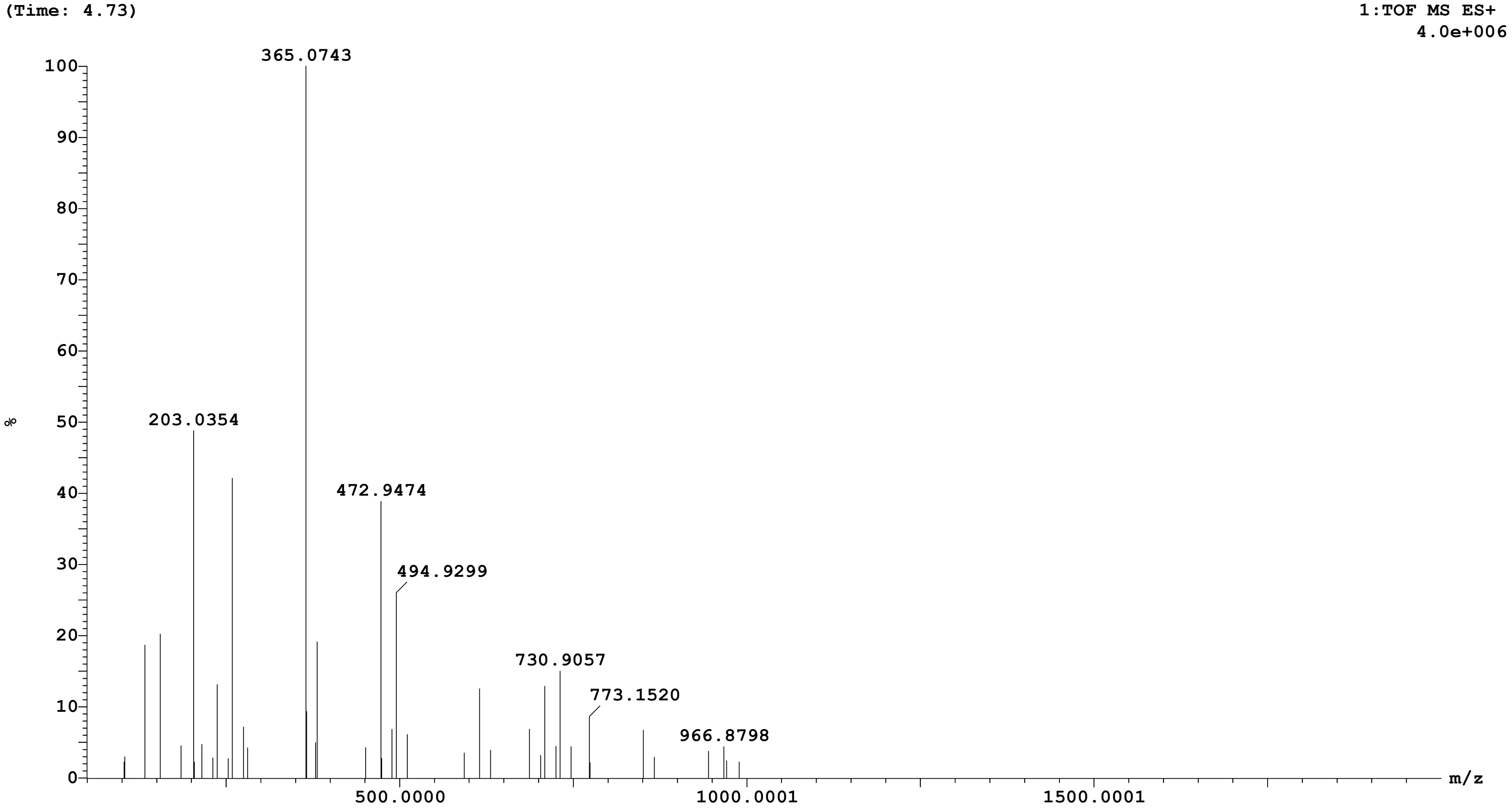

Sample: 66 Vial:1:F,3 ID:
File:AMISHA_S_2 Date:29-Oct-2025 Time:18:19:36
Description:

Printed: Thu Oct 30 16:32:41 2025

Peak ID	Time
10	6.38

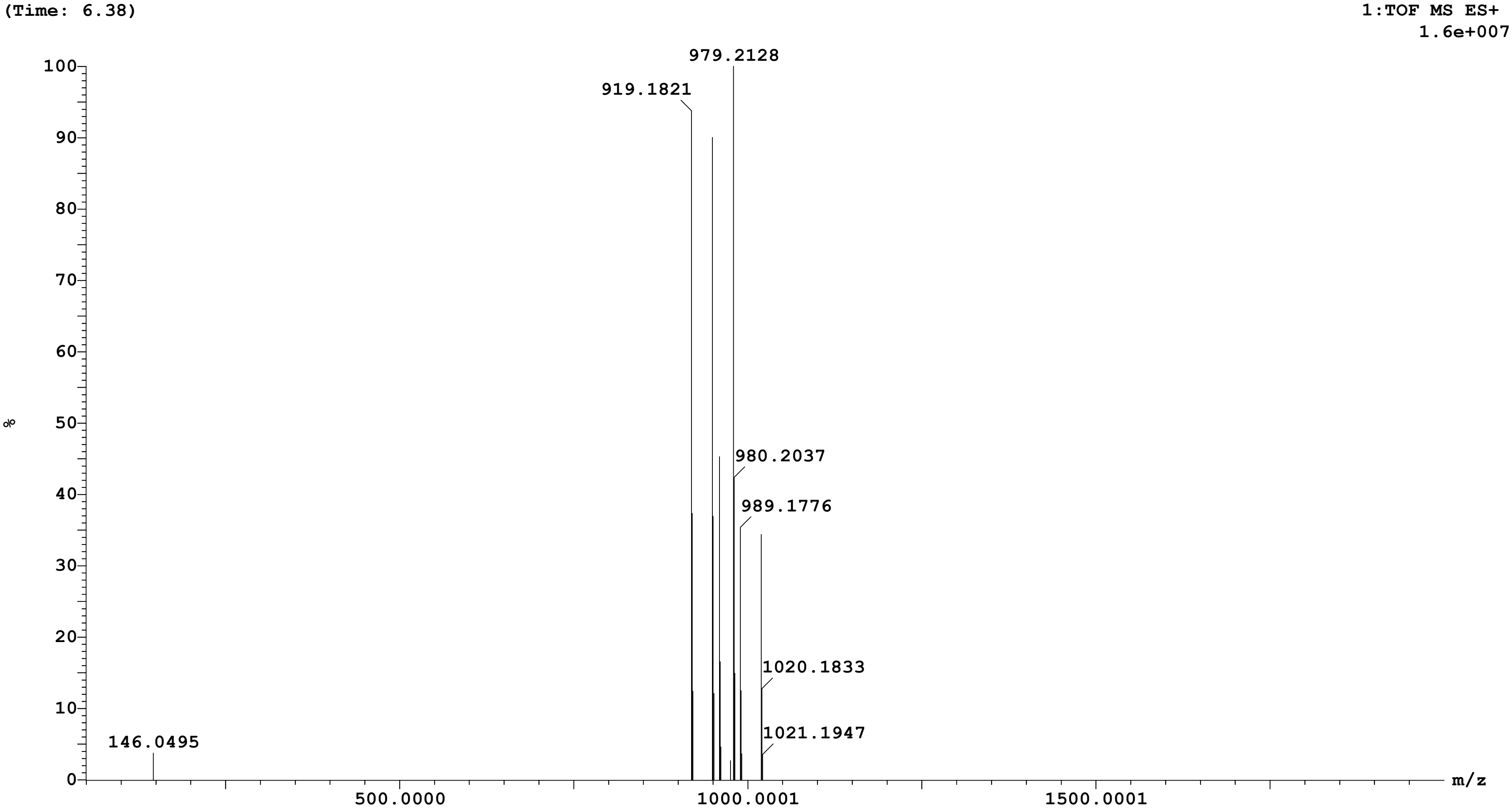

Sample: 66 Vial:1:F,3 ID:
File:AMISHA_S_2 Date:29-Oct-2025 Time:18:19:36
Description:

Printed: Thu Oct 30 16:32:41 2025

**Peak ID**	**Time**
11	10.97

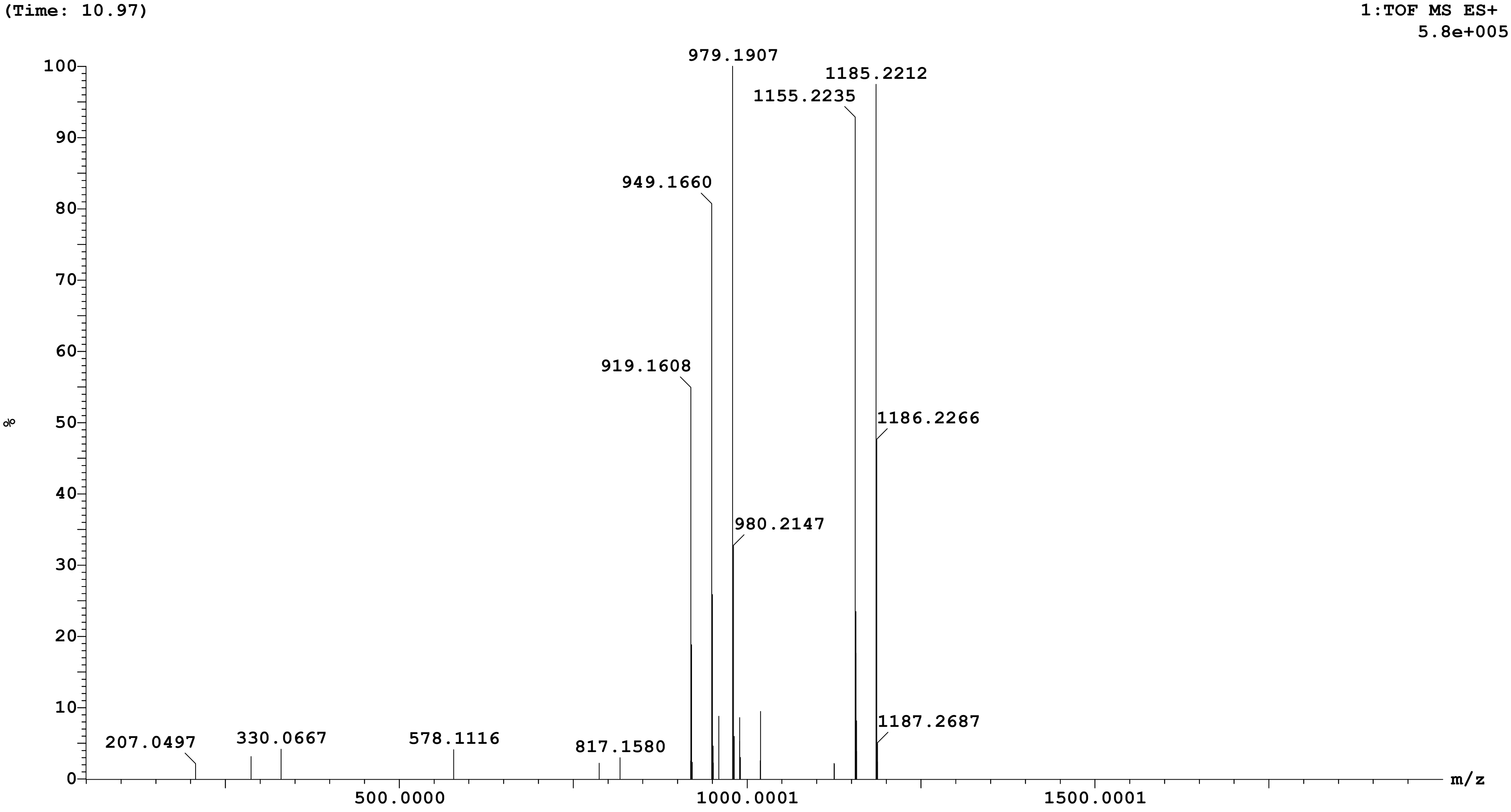

Sample: 66 Vial:1:F,3 ID:
File:AMISHA_S_2 Date:29-Oct-2025 Time:18:19:36
Description:

Printed: Thu Oct 30 16:32:41 2025

**Peak ID**	**Time**
13	14.30

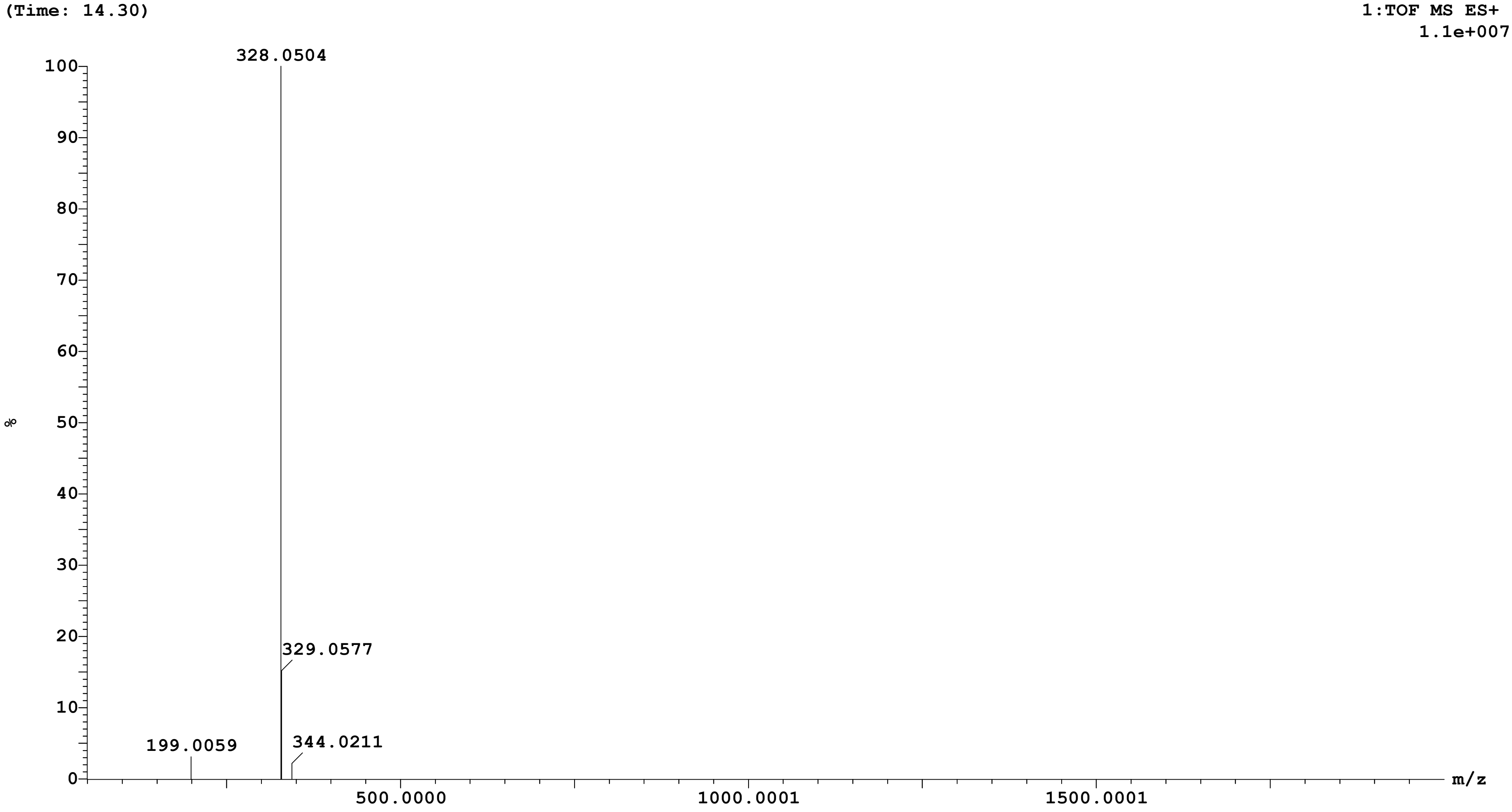

Sample: 66 Vial:1:F,3 ID:
File:AMISHA_S_2 Date:29-Oct-2025 Time:18:19:36
Description:

Printed: Thu Oct 30 16:32:41 2025

Peak ID	Time
14	15.19

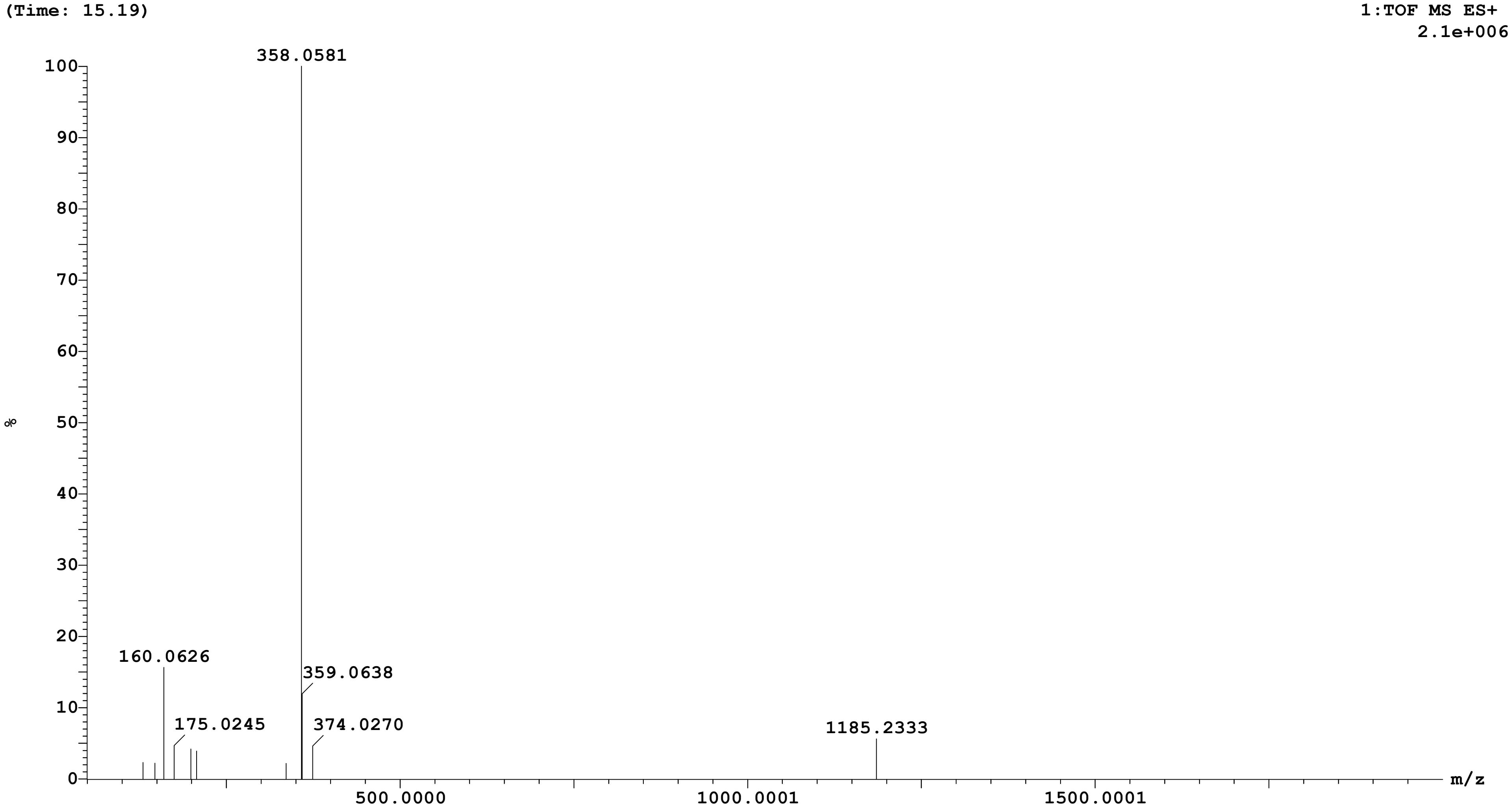

Sample: 66 Vial:1:F,3 ID:
File:AMISHA_S_2 Date:29-Oct-2025 Time:18:19:36
Description:

Printed: Thu Oct 30 16:32:41 2025

**Peak ID**	**Time**
15	16.47

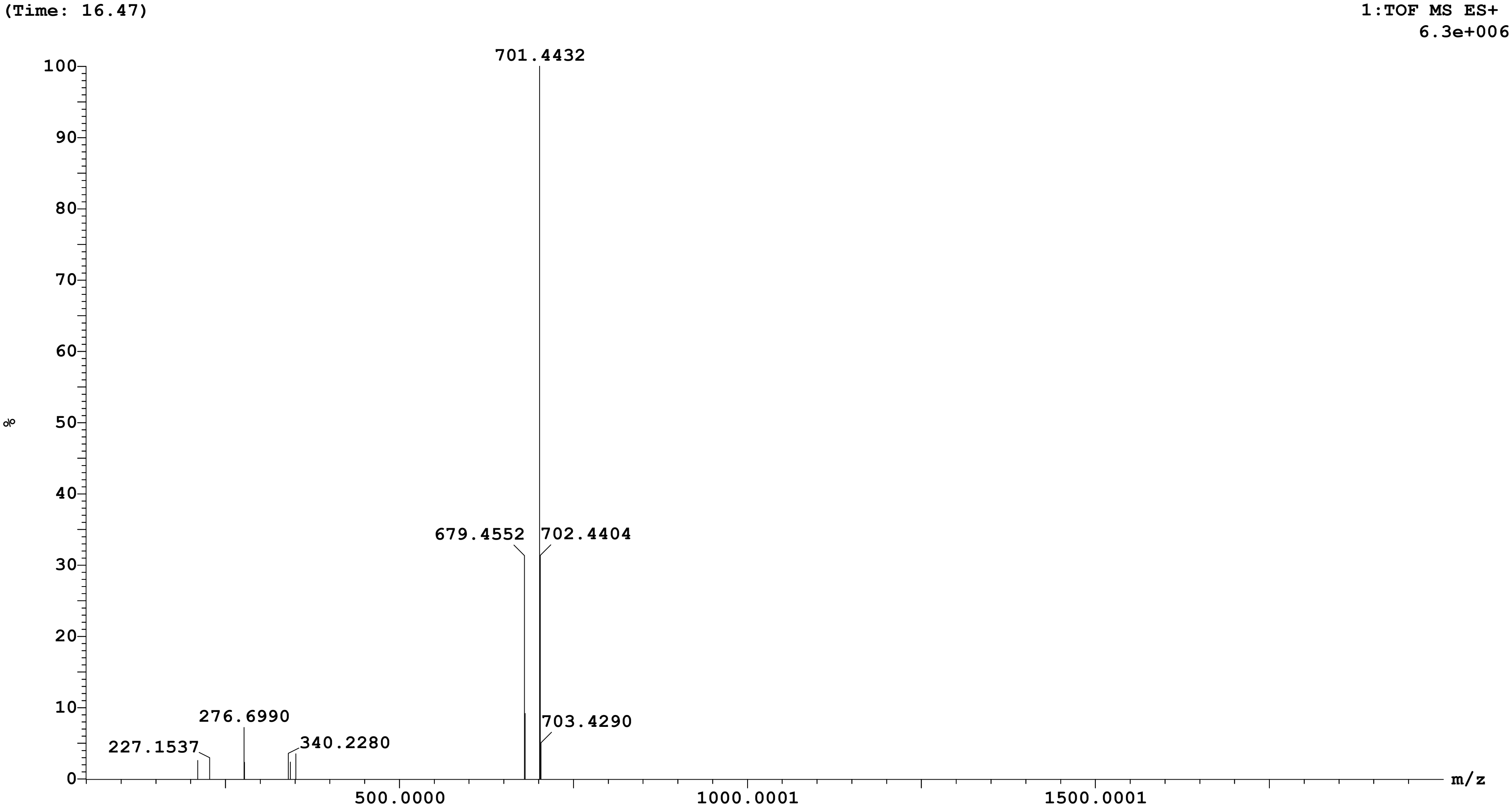

Sample: 66 Vial:1:F,3 ID:
File:AMISHA_S_2 Date:29-Oct-2025 Time:18:19:36
Description:

Printed: Thu Oct 30 16:32:41 2025

Peak ID	Time
16	17.95

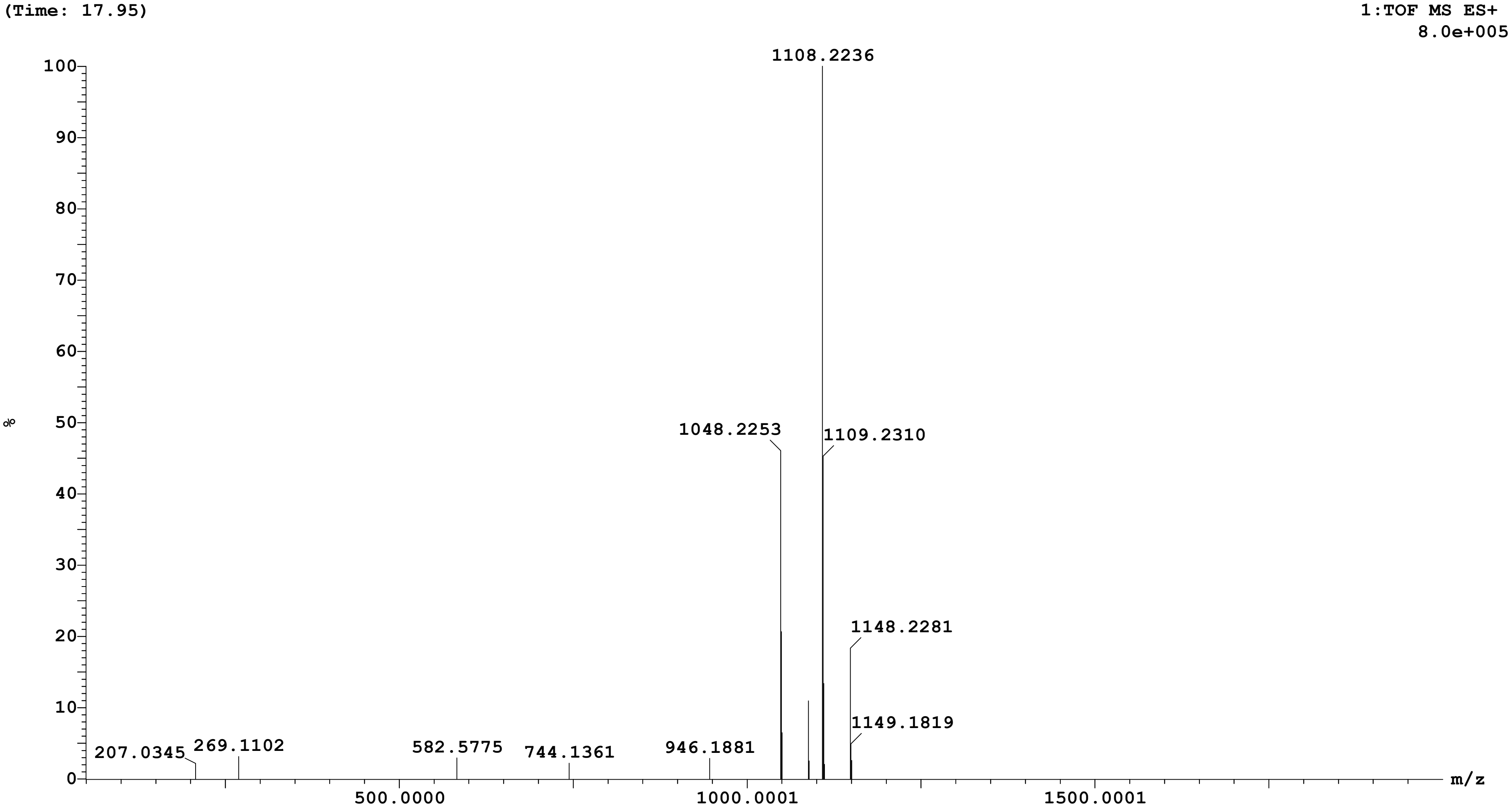

Sample: 66 Vial:1:F,3 ID:
File:AMISHA_S_2 Date:29-Oct-2025 Time:18:19:36
Description:

Printed: Thu Oct 30 16:32:41 2025

Peak ID	Time
17	19.70

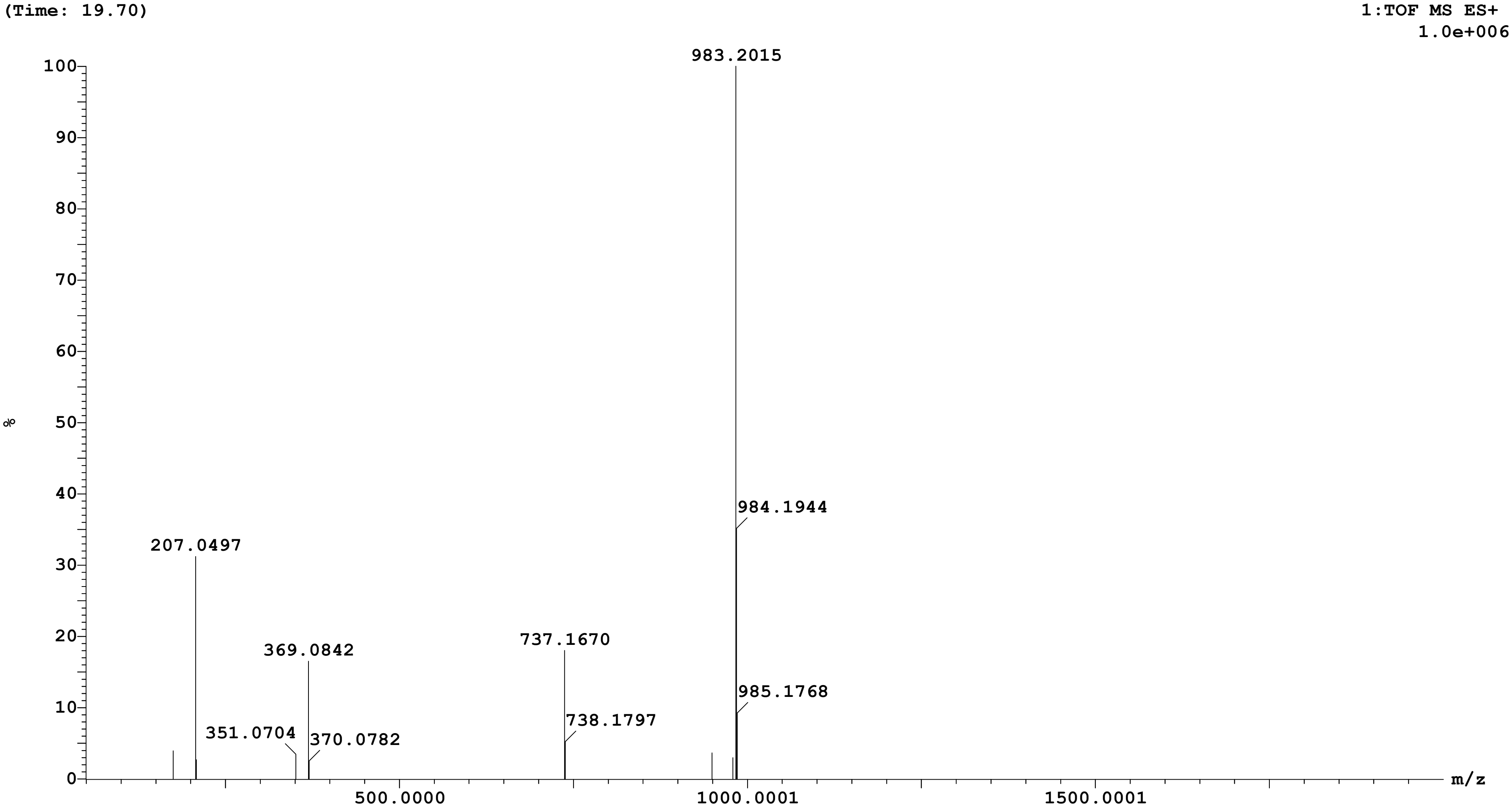

Sample: 66 Vial:1:F,3 ID:
File:AMISHA_S_2 Date:29-Oct-2025 Time:18:19:36
Description:

Printed: Thu Oct 30 16:32:41 2025

Peak ID	Time
18	22.16

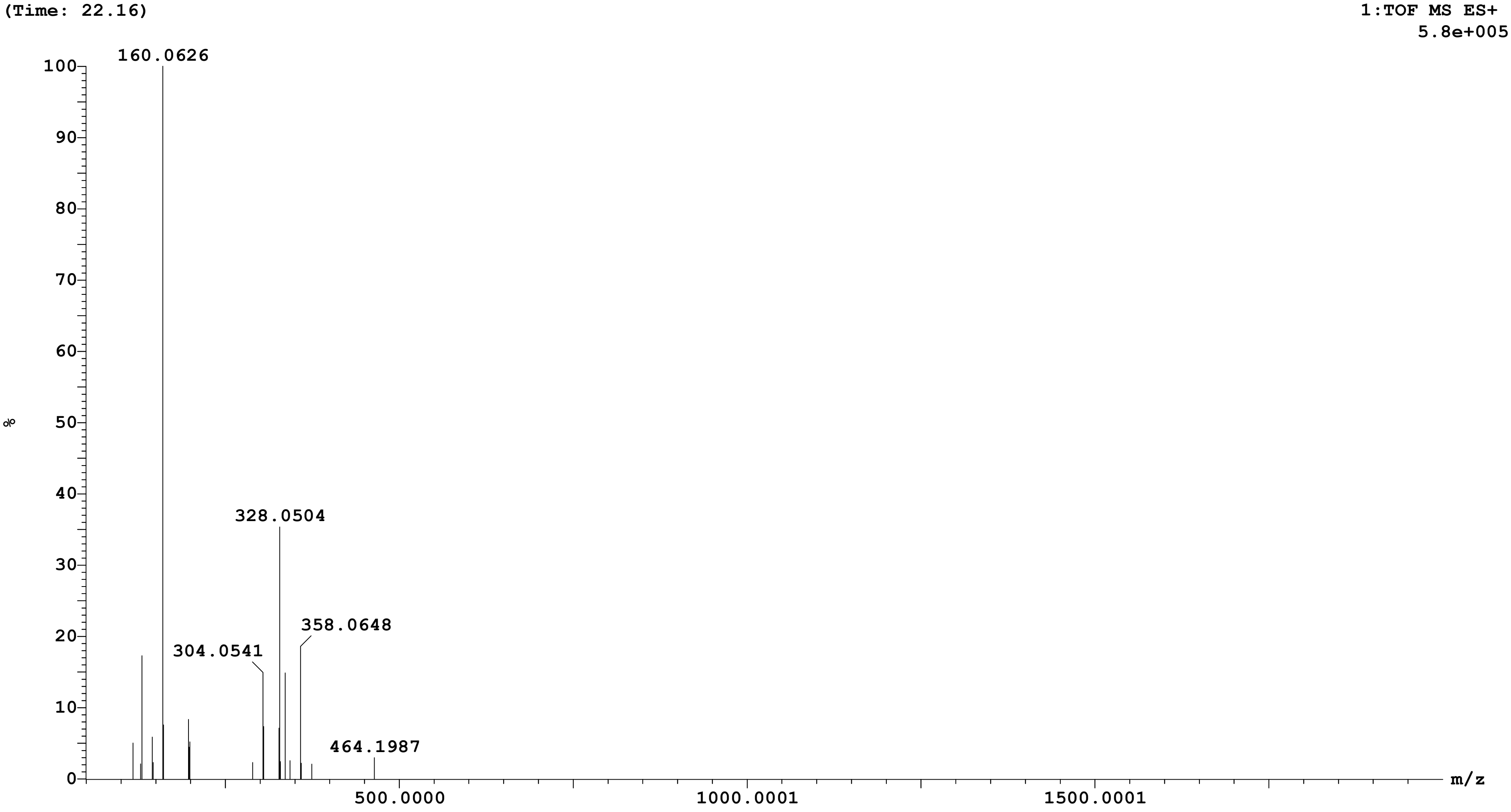

Sample: 66 Vial:1:F,3 ID:
File:AMISHA_S_2 Date:29-Oct-2025 Time:18:19:36
Description:

Printed: Thu Oct 30 16:32:41 2025

**Peak ID**	**Time**
19	56.39

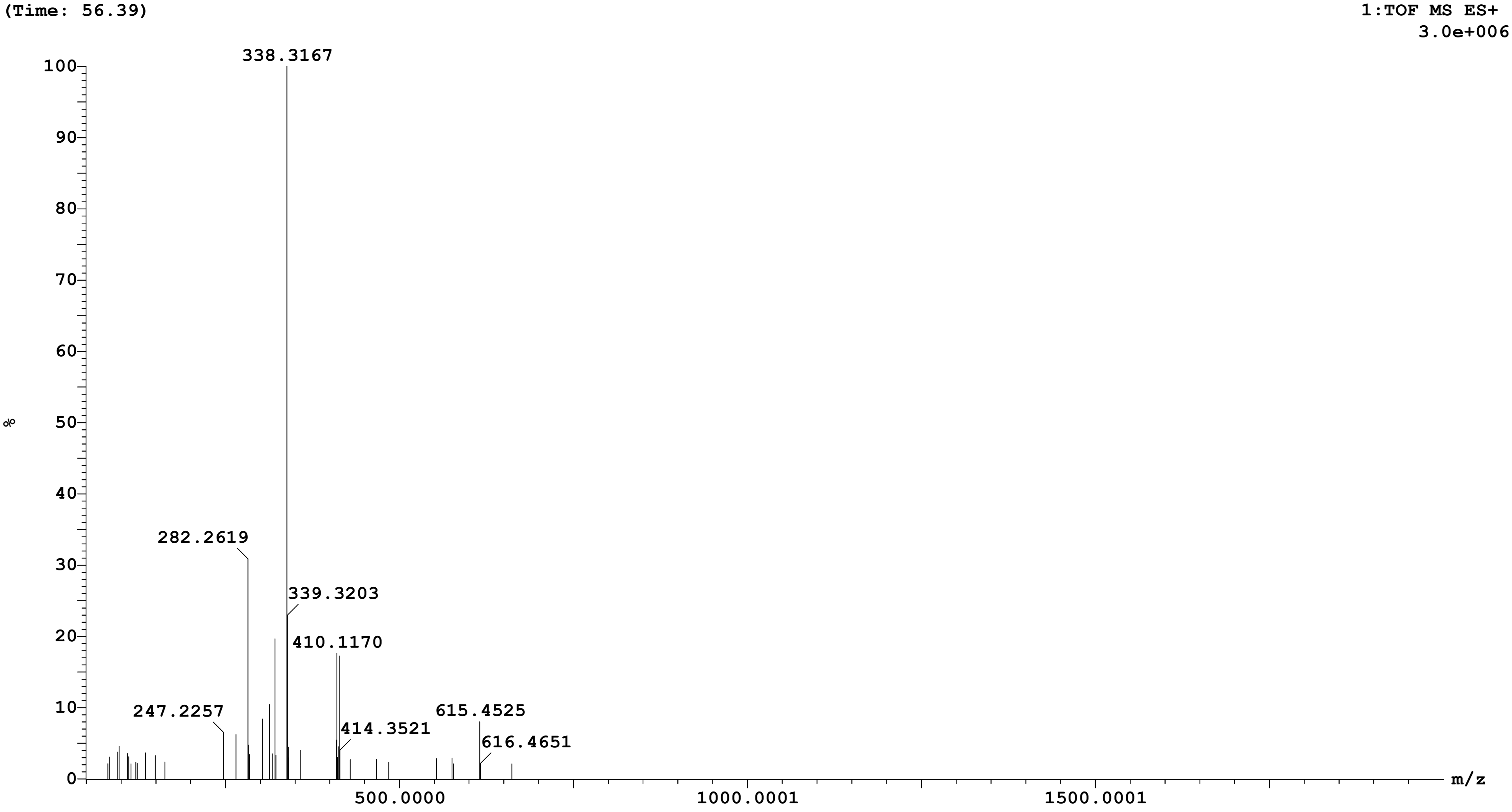

Sample: 66 Vial:1:F,3 ID:
File:AMISHA_S_2 Date:29-Oct-2025 Time:18:19:36
Description:

Printed: Thu Oct 30 16:32:41 2025

Peak ID	Time
2	4.22

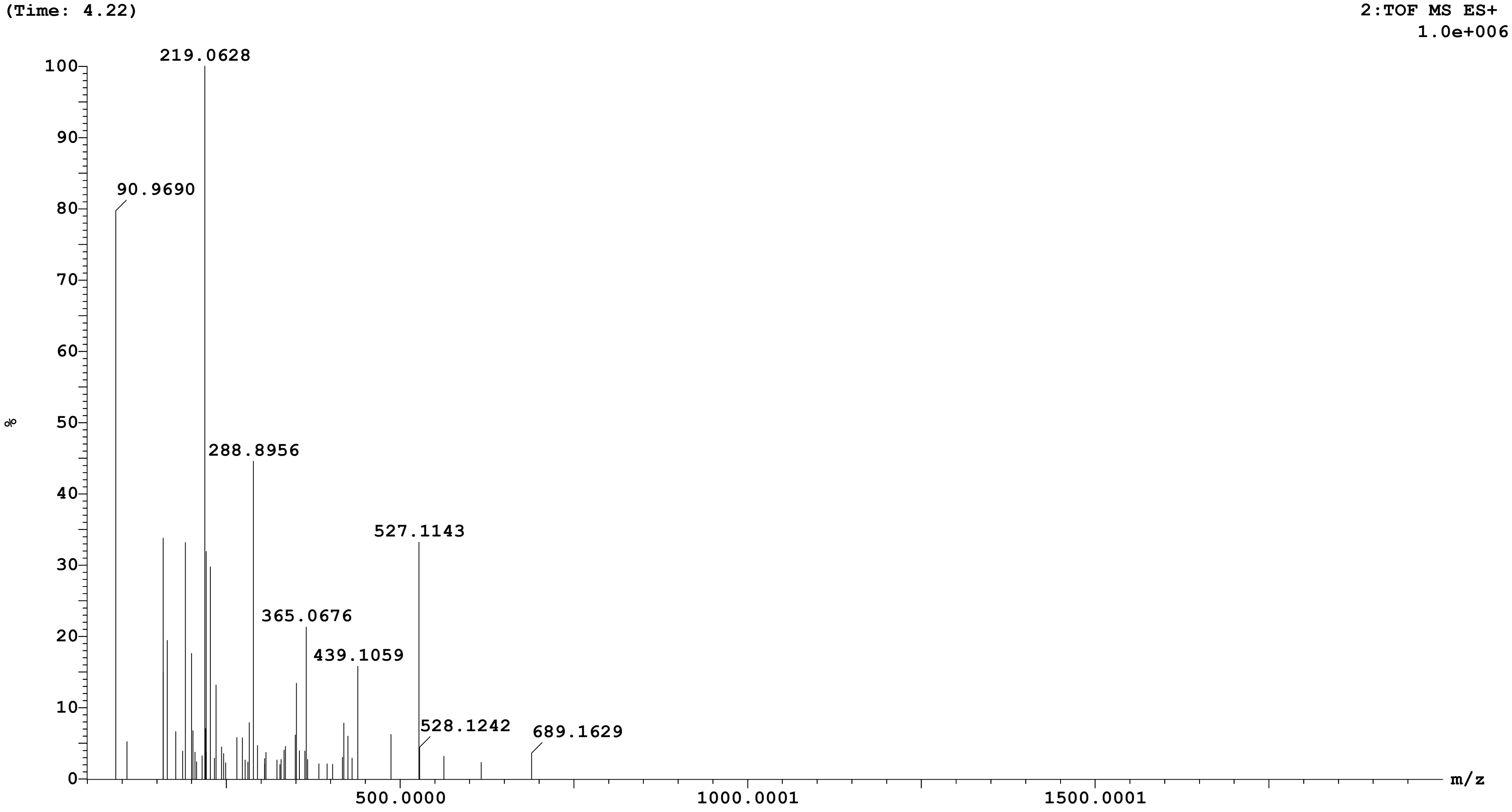

Sample: 66 Vial:1:F,3 ID:
File:AMISHA_S_2 Date:29-Oct-2025 Time:18:19:36
Description:

Printed: Thu Oct 30 16:32:41 2025

**Peak ID**	**Time**
6	4.85

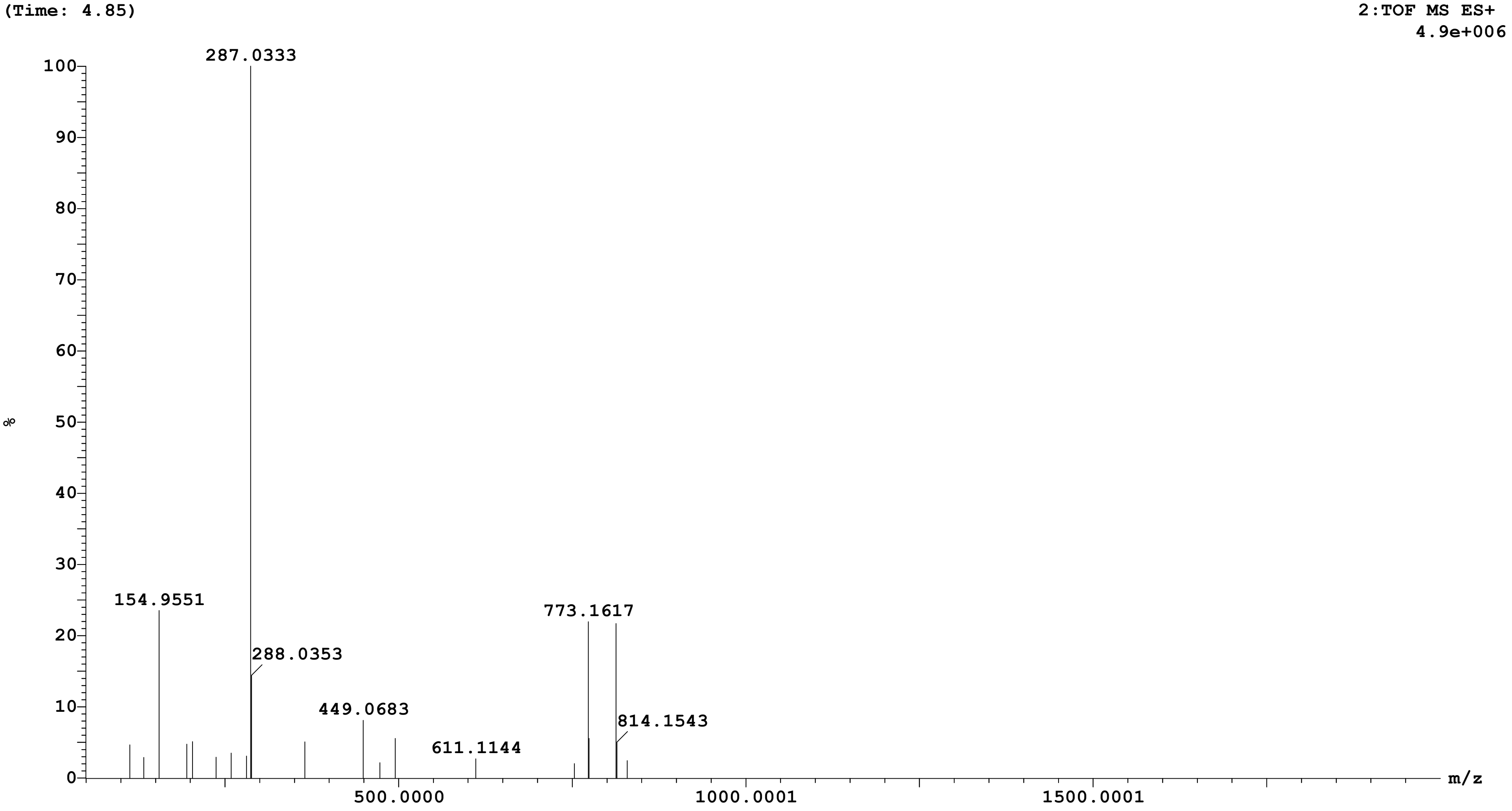

Sample: 66 Vial:1:F,3 ID:
File:AMISHA_S_2 Date:29-Oct-2025 Time:18:19:36
Description:

Printed: Thu Oct 30 16:32:41 2025

**Peak ID**	**Time**
9	5.77

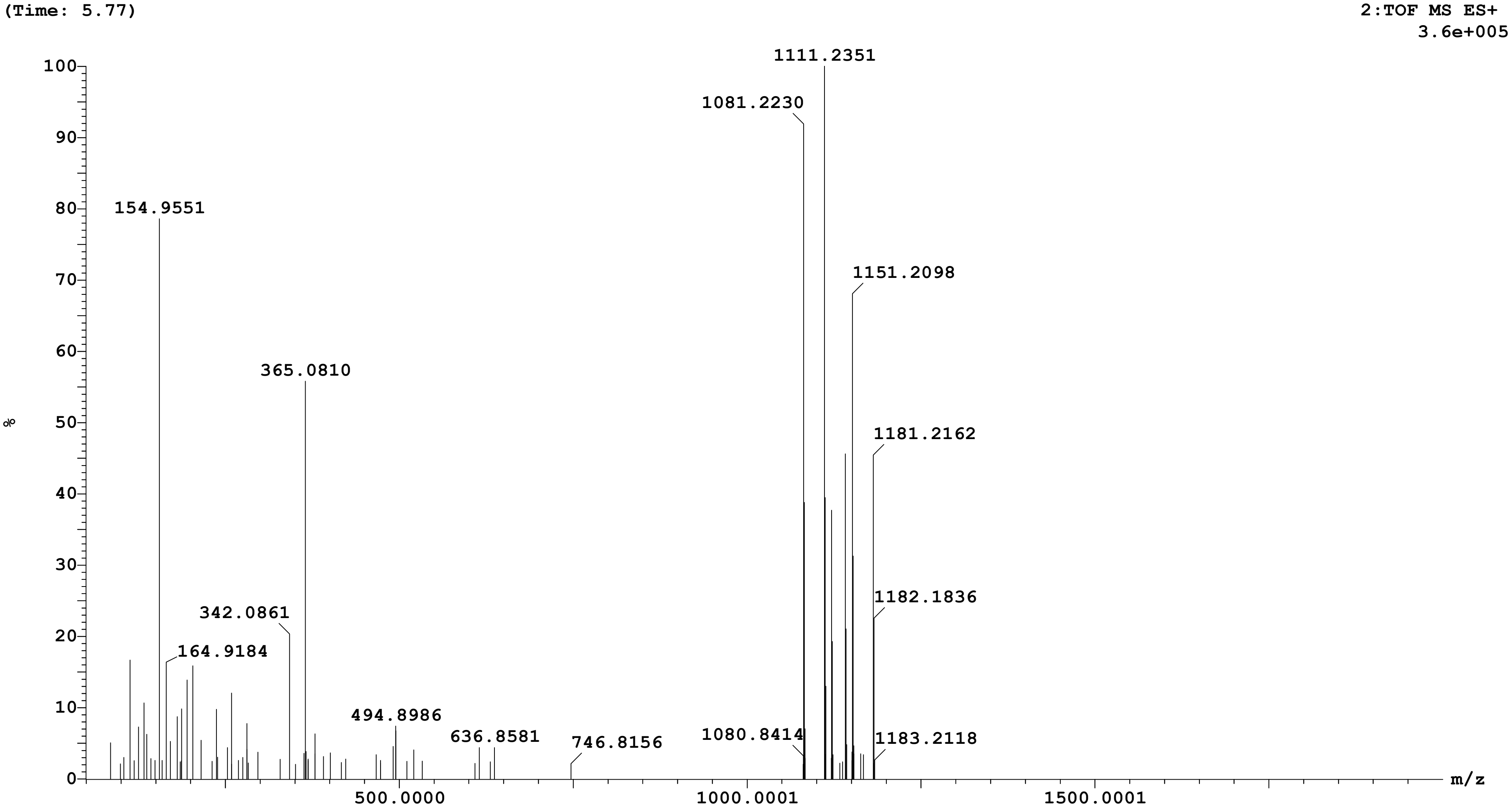

Sample: 66	Vial:1:F,3	ID:
File:AMISHA_S_2	Date:29-Oct-2025	Time:18:19:36
Description:		

Printed: Thu Oct 30 16:32:41 2025

**Peak ID**	**Time**
10	6.38

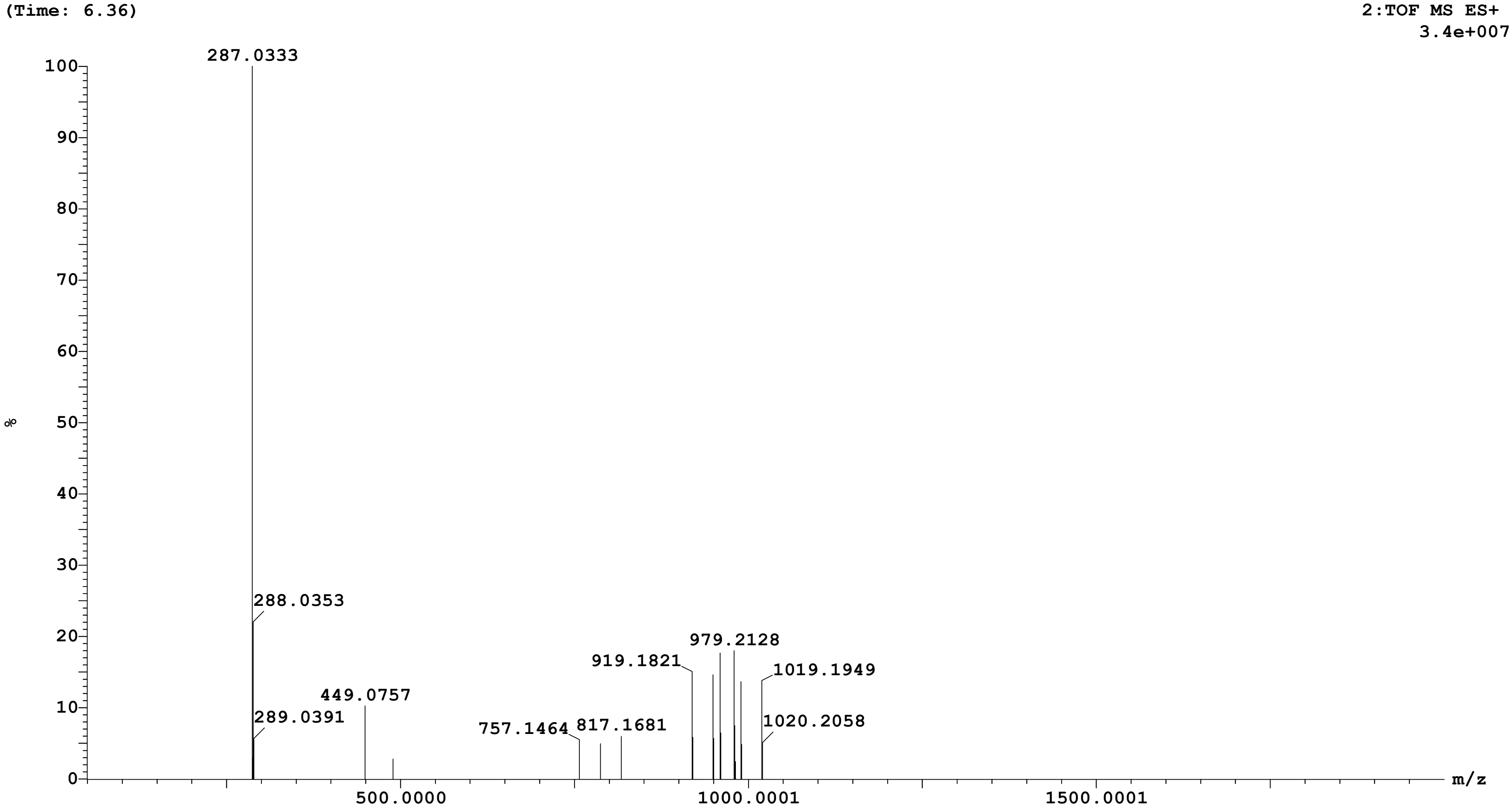

Sample: 66 Vial:1:F,3 ID:
File:AMISHA_S_2 Date:29-Oct-2025 Time:18:19:36
Description:

Printed: Thu Oct 30 16:32:41 2025

**Peak ID**	**Time**
12	11.16

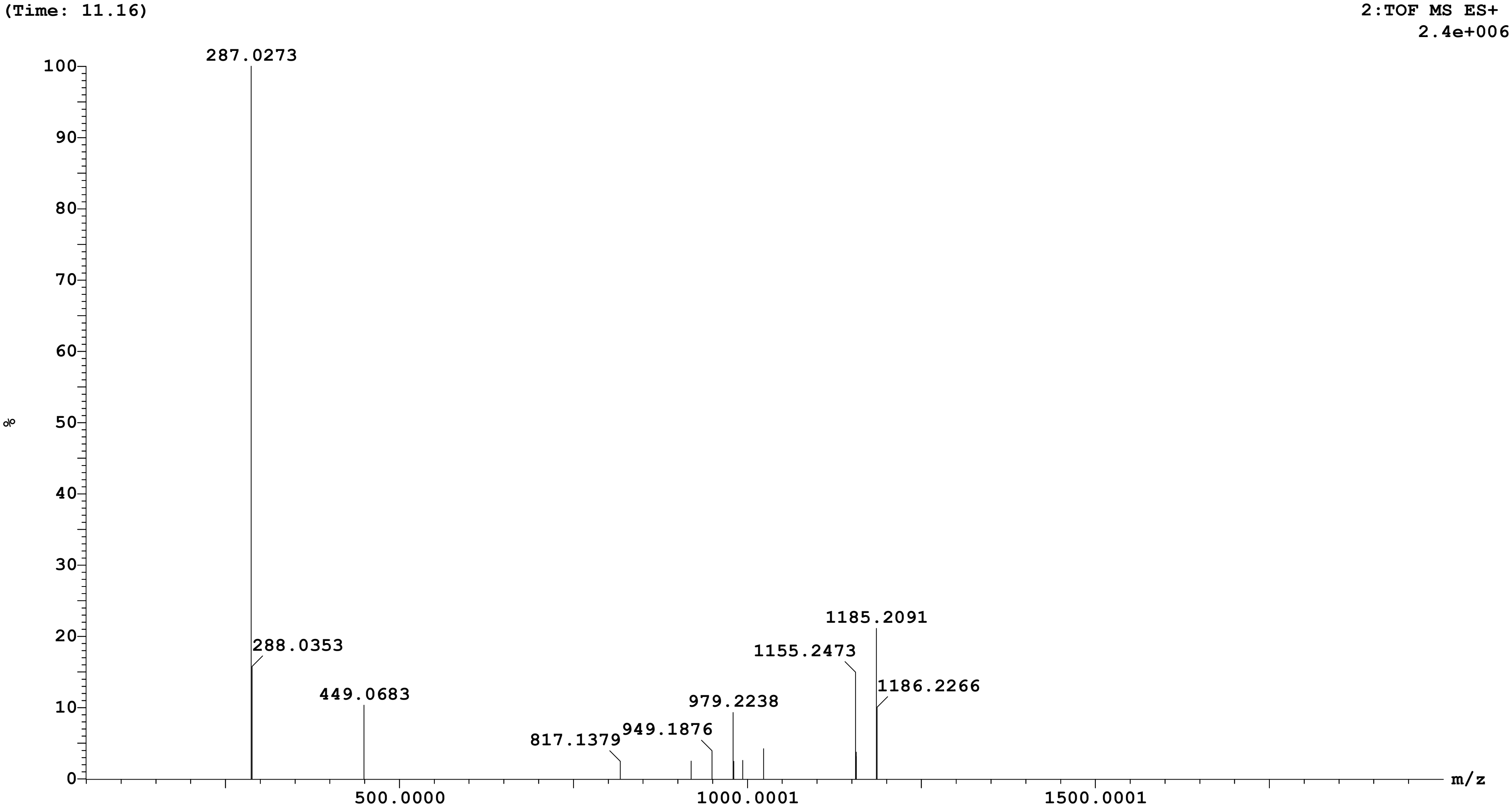

Sample: 66 Vial:1:F,3 ID:
File:AMISHA_S_2 Date:29-Oct-2025 Time:18:19:36
Description:

Printed: Thu Oct 30 16:32:41 2025

Peak ID	Time
21	56.74

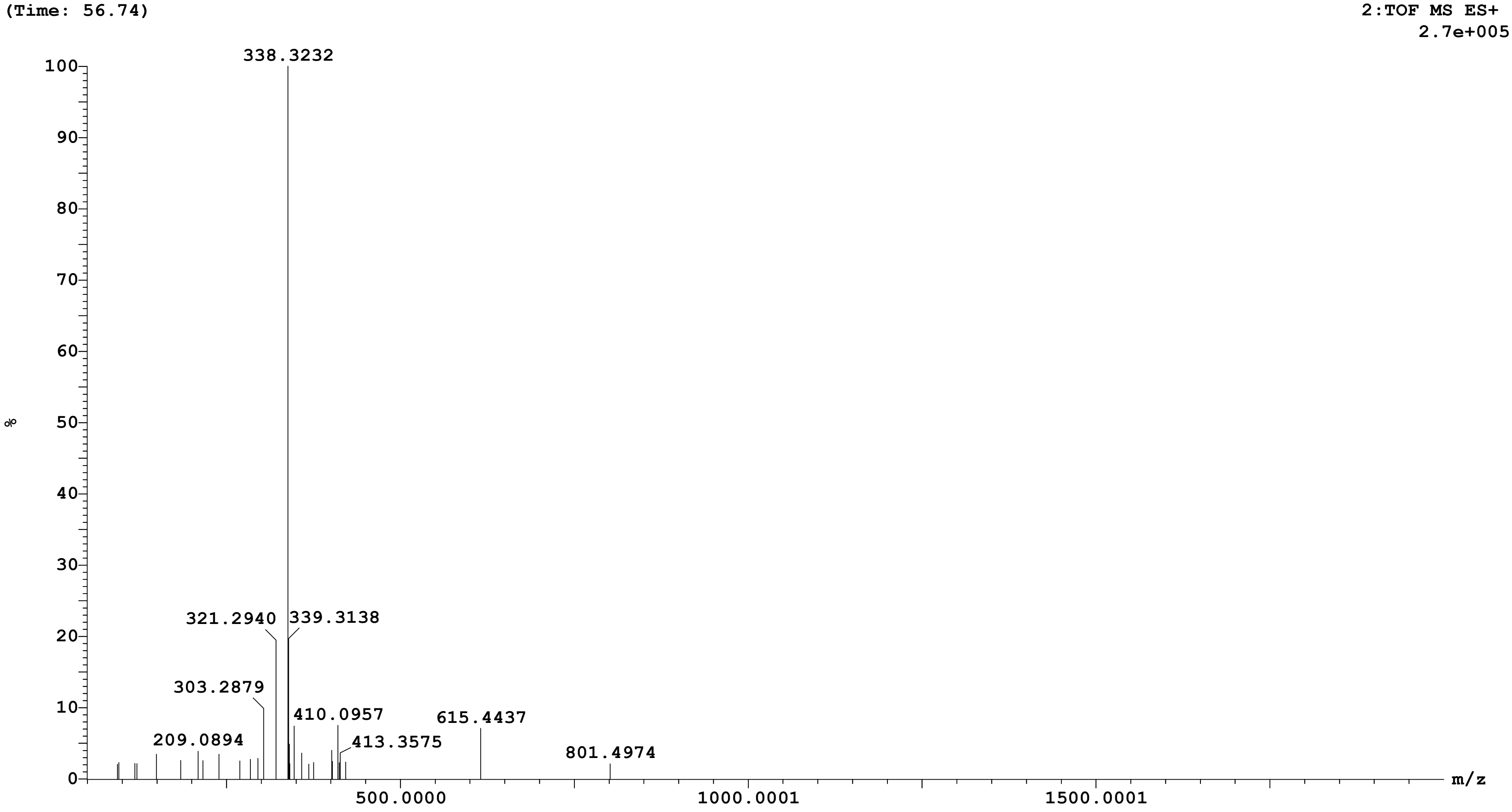

Supplement: Supplementary Data 2 [file mmc2.pdf]
